# Predicting Mood Based on the Social Context Measured Through the Experience Sampling Method, Digital Phenotyping, and Social Networks

**DOI:** 10.1007/s10488-023-01328-0

**Published:** 2024-01-10

**Authors:** Anna M. Langener, Laura F. Bringmann, Martien J. Kas, Gert Stulp

**Affiliations:** 1 Groningen Institute for Evolutionary Life Sciences, University of Groningen, Groningen, The Netherlands; 2 Department of Psychometrics and Statistics, Faculty of Behavioural and Social Sciences, University of Groningen, Groningen, The Netherlands; 3 Interdisciplinary Center Psychopathology and Emotion Regulation, (ICPE), University Medical Center Groningen, University of Groningen, Groningen, The Netherlands; 4 Department of Sociology & Inter-University Center for Social Science Theory and Methodology, Grote Rozenstraat 31, 9712 TS Groningen, The Netherlands; 5 Faculty of Science and Engineering, Nijenborgh 7, 9747 AG Groningen, The Netherlands

**Keywords:** Social behavior, Social interactions, Random forest, Time scales, Individualized machine learning models, Shapley values

## Abstract

Social interactions are essential for well-being. Therefore, researchers increasingly attempt to capture an individual's social context to predict well-being, including mood. Different tools are used to measure various aspects of the social context. Digital phenotyping is a commonly used technology to assess a person's social behavior objectively. The experience sampling method (ESM) can capture the subjective perception of specific interactions. Lastly, egocentric networks are often used to measure specific relationship characteristics. These different methods capture different aspects of the social context over different time scales that are related to well-being, and combining them may be necessary to improve the prediction of well-being. Yet, they have rarely been combined in previous research. To address this gap, our study investigates the predictive accuracy of mood based on the social context. We collected intensive within-person data from multiple passive and self-report sources over a 28–day period in a student sample (Participants: N = 11, ESM measures: N = 1313). We trained individualized random forest machine learning models, using different predictors included in each model summarized over different time scales. Our findings revealed that even when combining social interactions data using different methods, predictive accuracy of mood remained low. The average coefficient of determination over all participants was 0.06 for positive and negative affect and ranged from − 0.08 to 0.3, indicating a large amount of variance across people. Furthermore, the optimal set of predictors varied across participants; however, predicting mood using all predictors generally yielded the best predictions. While combining different predictors improved predictive accuracy of mood for most participants, our study highlights the need for further work using larger and more diverse samples to enhance the clinical utility of these predictive modeling approaches.

**Supplementary Information:**

The online version contains supplementary material available at 10.1007/s10488-023-01328-0.

The social context plays a crucial role in mental health (Blanco et al., 2008; Teo et al., 2018). Whereas positive social interactions can enhance mood, a lack of social interactions is a risk factor for low mental well-being (Blanco et al., 2008; Teo et al., 2018). Moreover, the onset of psychological disorders is often characterized by lower engagement in social interaction (Krach et al., 2010; Perry & Pescosolido, 2012). Thus, understanding features of social interactions that predict mood and well-being is important to the assessment and treatment of psychopathology.

A person’s social context is made up of complex interactions between various components of social experience. The first component of social experience includes (daily) social situations and interactions (Phongsavan et al., 2006). These interactions form social relationships that develop over time and make up a person’s social environment (Asendorpf & Rauthmann, 2020), sometimes referred to as a personal network. A second component of social context includes the perceptions of these interactions and relationships, for example whether an interaction was perceived as pleasant or unpleasant (Asendorpf & Rauthmann, 2020). These perceptions are sometimes referred to as the psychological component or cognitive representation (Harpham, 2002; Phongsavan et al., 2006). Research has shown that interactions between these components are closely linked to an individual’s well-being (Brown et al., 2011; Krach et al., 2010; Perry & Pescosolido, 2012). To measure components of the social context, several technologies from various disciplines have been developed (Langener et al., 2023). Three commonly used methods are (a) digital phenotyping; which passively captures a person’s social behavior, (b) the experience sampling method (ESM); which measures perceptions of social situations, and (c) egocentric networks; which assess an individual's personal social network that form a person’s social environment.

Digital phenotyping refers to the collection of passive data via smartphones and wearable devices, such as data on phone calls, text messages, app usage, GPS, Wi-Fi, and movement, measured continuously throughout the day (Torous et al., 2016). These data are sometimes considered more objective than self-reported data and are collected with lower respondent burden. Previous studies have suggested that passive smartphone measures can provide insights into an individual’s social behavior (Burns et al., 2016; Eskes et al., 2016; Fulford et al., 2020; Stanislaus et al., 2020). However, results from previous work have been inconsistent, and the specificity of passive smartphone measures for capturing aspects of an individual’s social behavior remains unclear (Langener et al., 2023). As an example, the association between the distance traveled (measured per GPS) and number of interactions differed between people with schizophrenia (r = 0.07) and healthy controls (r = 0.6; Fulford et al., 2021) complicating the interpretation of ‘distance traveled’. Despite this limitation, we consider passive smartphone measures as potential indicators of an individual's social behavior, with the important constraint that we do not know which specific aspect they capture.

ESM can measure multiple components of social situations, including one’s daily social interactions, as well as their perception of these specific interactions (e.g., Čolić et al., 2020; Mills et al., 2014). Within the ESM, participants fill out brief questionnaires using their mobile phone several times a day after a push notification is sent (Kubey et al., 1996). Long ESM questionnaires are burdensome for the participants (Eisele et al., 2020) but can cover social situations more broadly than digital phenotyping.

To assess information on long-term social relationships, egocentric networks are often constructed. Researchers typically start by asking the participant to list the names of important social contacts with whom they are close with or have interacted with during a period of time. After those contacts are identified, further details about the characteristics of these contacts and the relationship with these contacts are assessed (Perry et al., 2018), including social role of the contact (e.g., partner, family, friend) or level of respondent closeness to members in the network. This approach can measure facets of support that are more difficult to assess through ESM or digital phenotyping.

Although these methods capture different aspects of the social context over different time scales that relate to well-being, they have rarely been combined in previous research (Langener et al., 2023). Combining data from multiple sources may help to increase the precision with which we measure the impact of social context on health. Previous research studies have often focused solely on using digital phenotyping to measure social situations and predict mood, but the predictive accuracy is often low to moderate (e.g., Abdullah et al., 2016; Jacobson & Chung, 2020; Jacobson et al., 2020). Complementing digital phenotyping with data from other self-reported aspects of the social context may be needed to improve predictive accuracy and thus increase clinical utility (Currey & Torous, 2022).

## The Current Study

In this study, we integrated data from three methods: digital phenotyping, ESM, and egocentric networks to construct individualized random forest machine learning models to investigate how aspects of social context predict positive and negative affect. We used individualized random forest machine learning models, as they are shown to outperform non-individualized prediction models yet are rarely used in research on well-being (Abdullah et al., 2016; Benoit et al., 2020; Cai et al., 2018; Hart et al., 2012). Results from individualized prediction models also offer the potential to deliver just-in-time interventions, which are personalized interventions delivered directly to individuals when needed most (Nahum-Shani et al., 2017).

We measured social context over various time scales using digital phenotyping, ESM, and egocentric networks. Digital phenotyping measures social behavior near continuously throughout the day, while ESM measures social situations at specific time points (i.e., in reference to a short period of time before the questionnaire was completed). Egocentric networks focus on the constant characteristics of the social environment and are often assessed only once or twice within a period of time. To integrate data across different temporal resolutions, we enhanced ESM with data obtained from egocentric networks (Stadel et al., 2023). Additionally, we investigated the impact of a given time scale on prediction performance. We further examined the extent to which these different methods uniquely predicted mood to identify the most important measurements in predicting health outcomes.

## Methods

### Participants and Procedure

We used data from a student sample collected in 2022 (N = 15, female = 14, male = 1). Participants were on average 22 years old (SD = 4.10, min = 18, max = 35). A detailed description of the study procedure can be found in the respective Open Science Framework (OSF) repository (https://osf.io/jqdr9/). The study was approved by the ethics board of the University of Groningen (research code: PSY-2223-S-0018). The analyses were preregistered before being conducted (https://osf.io/738pr).

The participants filled out ESM questionnaires for 28 days. During those 28 days, participants received five semi-random questionnaires measuring positive and negative affect. Participants were also instructed to record any social interactions that lasted longer than 5 min. Social interactions were defined according to Hall's (2018a) definition of a focused social interaction, which includes “(1) mutual acknowledgment by both partners of a shared relationship, (2) conversational exchange, and (3) focused attention by both partners on that exchange”. Thus, mutual text messaging was also included in the assessment. We used two different assessment formats to capture social interactions—which we refer to as signal- and event-based reporting (Myin-Germeys & Kuppens, 2022). Each format was used for 2 weeks, with the order balanced between participants. In the signal-based reporting format, participants were asked to retrospectively report all their social interactions since the last questionnaire during the daily scheduled semi-random questionnaires. In contrast, during the event-based format, participants were required to report social interactions immediately after they occurred. In both conditions, respondents filled out the same scheduled questionnaires to evaluate their mood and daily activities. An overview of all questions asked can be found here: https://osf.io/5hmdz.

Alongside the ESM, participants installed an app (Behapp) that collected data passively for 28 days (Jagesar et al., 2021). On Android devices data on location, calls, texts, Wi-Fi, screen states, and app usage was collected. At the time of this data collection, only location data was collected on IOS devices (although Behapp now collects more than location data on IOS devices).

Participants completed an egocentric network questionnaire to assess their social relationships before and after the ESM period. We asked the participants to list names from their social network with whom they have contact with in their daily lives. Subsequently, further questions about the relationship characteristics were asked, for example, how close they are to a specific person. An overview of all the questions asked in the egocentric network can be found at https://osf.io/8zg3x. In addition, the nicknames of interaction partners were recorded during the ESM period, which allowed us to link names from the egocentric network with those from the ESM period to gain insight into characteristics of interaction partners (for more information see Stadel et al., 2023).

As stated in the preregistration three participants were excluded because they did not own an Android phone and/or had less than 75% of passively collected data via Behapp. Additionally, one participant was excluded because they filled out less than 75% of the scheduled ESM data. This results in a total sample of 11 participants (female = 10, male = 1, total included ESM measures, N = 1313).

### Measures

#### Outcome Variable

We aimed to predict positive affect and negative affect as measured during the scheduled questionnaires. Positive affect was assessed by taking the mean of the answers to three items (“I feel happy”, “I feel energetic”, “ I feel relaxed”), and negative affect was assessed by taking the mean of the answers to four items (“I feel sad”, “I feel anxious”, “I feel stressed”, I feel irritated”). The questions were measured using a slider that was labeled with *Strongly disagree* (left side) and *Strongly agree* (right side) on an 11-point Likert scale, which we rescaled to one to 11 for ease of calculation.

#### Predictors

**Fig. 1:**
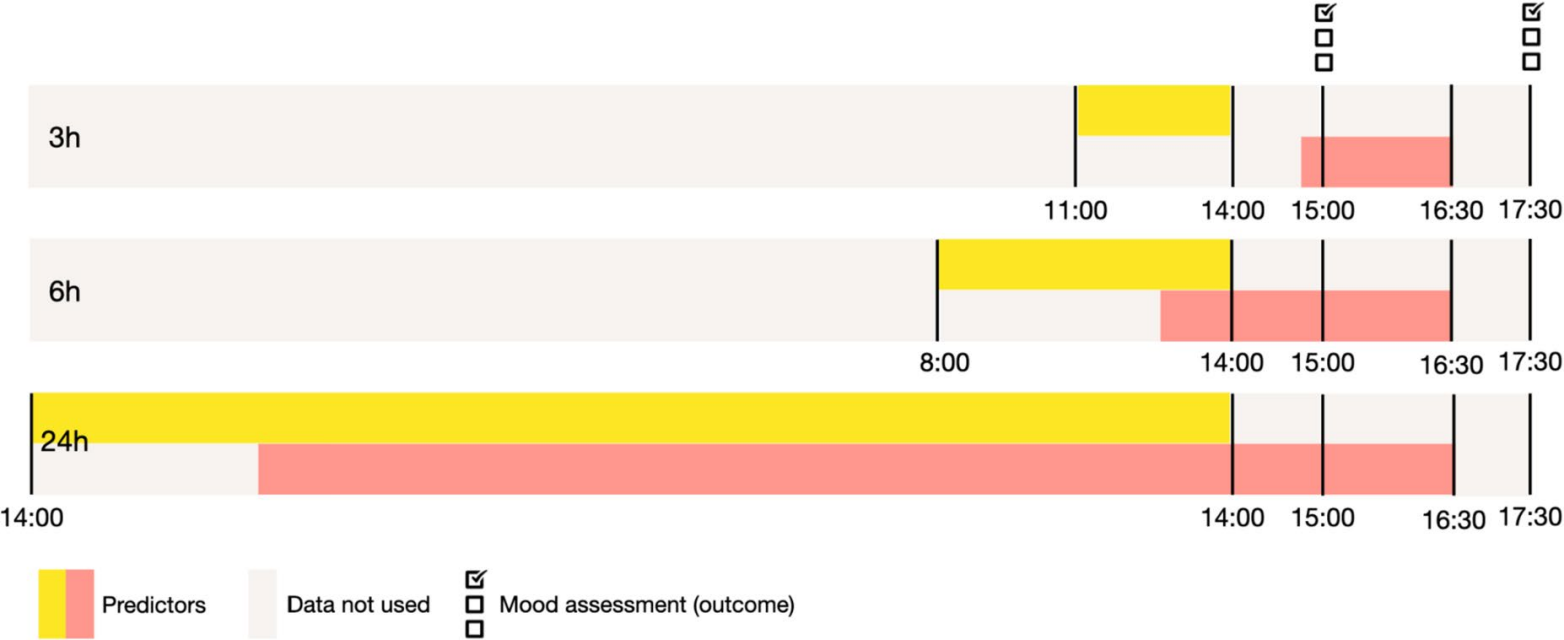
Illustration of an example for summarizing the predictors in a 3, 6, and 24 h time window with a time lag of 60 minutes for two selected ESM measurement

In Table 1, we summarize the predictors used to assess an individual’s social behavior. These behaviors were aggregated in 3, 6, and 24 h time windows before the scheduled ESM questionnaire was filled out. For example, data collected between 11:00 and 14:00 (i.e., a 3-h time window) could be used to measure social behavior that occurred before the ESM questionnaire was filled out at 15:00 (see also Fig. 1). We excluded data collected during the hour prior to ESM measurement (between 14:00 and 15:00 in the above example) to predict mood in advance (illustrated in Fig. 1). As a robustness check, we also completed analyses excluding data collected during the 30 min prior to ESM measurement.

**Table 1:** Overview of all predictors

Source/sensor	Created variable
App	∙ Minutes spent on all apps ∙ Minutes spent on communication apps ∙ Minutes spent on social media apps ∙ Minutes spent on WhatsApp ∙ Number of apps opened
Location	∙ The number of clustered staypoints ∙ Minutes being at home ∙ Minutes spent stationary ∙ The average distance traveled from home
Call	∙ Total duration of calls (in minutes) ∙ Total duration of incoming calls (in minutes) ∙ Total duration of outgoing calls (in minutes)
Wi-Fi	∙ Unique number of Wi-Fi hotspots ∙ Total number of Wi-Fi hotspots
Screen	∙ Times screen is locked ∙ Times screen is unlocked
ESM (social interaction questionnaire)	∙ Total minutes spent in face-to-face interactions ∙ Total minutes spent calling (phone call or video call) ∙ Total minutes spent texting ∙ Total minutes spent in conversations with the content “striving behavior” (i.e., expressing love or affection, joking around, meaningful, catching up; face-to-face, call, text) ∙ Total minutes spent in conversations with the content “mundane maintenance behavior” (i.e., gossip, task talk, small talk, making plans, face-to-face, call, text) ∙ Total minutes spent in conversations with the content “work or school talk” ∙ Total minutes spent in conversations with negative content (i.e., complaining or venting, conflict or disagreement; face-to-face, call, text) ∙ Last interaction: I enjoyed the interaction ∙ Last interaction: my interaction partner enjoyed the interaction ∙ Last interaction: meaningful ∙ Last interaction: could be myself ∙ Last interaction: cost me energy ∙ Last interaction: gave energy ∙ Last interaction: during the interaction I felt happy ∙ Last interaction: how long ago
Egocentric network	∙ Duration of interactions with partner ∙ Duration of interactions with a friend ∙ Duration of interactions with a family (parent, sibling, relative) ∙ Duration of interactions with a fellow student/colleague ∙ Duration of interactions with a flatmate ∙ Duration of interactions with superior/teacher ∙ Duration of interactions with close friends/ family (yes/no) ∙ Duration of interactions with a partner that someone can discuss personal issues with (yes/no) ∙ Duration of interactions with a partner that provides emotional support (yes/no) ∙ Duration of interactions with a partner that provides practical/ material support (yes/no) ∙ Last interaction partner: closeness (five-point Likert scale) ∙ Last interaction partner: gives energy (five-point Likert scale) ∙ Last interaction partner: costs energy (five-point Likert scale) ∙ Last interaction partner: be myself (five-point Likert scale) ∙ Last interaction partner: face-to-face contact frequency (five-point Likert scale) ∙ Last interaction partner: call contact frequency (five-point Likert scale) ∙ Last interaction partner: text contact frequency (five-point Likert scale)

We used time windows of 3 and 6 h to measure social behavior that might more immediately impact mood, such as a specific social interaction that occurred recently before the mood assessment. We used the 24 h time window to measure impactful social interactions that occur less frequently (e.g., meeting up with friends at night) or patterns of behavior that occur over extended periods (e.g., being at work for several hours). Longer time scales also can be used to measure irregularities in an individual’s behavior that may indicate changes in mood, for example, if a person suddenly does not leave the house for a whole day (Cai et al., 2018).

We derived several variables from raw passive smartphone measures to measure aspects of social interaction. Some measures, such as total minutes of calling, were directly summarized into a variable in the aggregated dataset at a specific temporal resolution. Other measures, such as GPS coordinates, required preprocessing steps before they can be aggregated. To derive meaningful location variables, we clustered GPS coordinates into staypoints, where a staypoint is any location within a radius of 350 m where participants stayed for at least 30 min (Zheng et al., 2009). The staypoint where the participant spent most nights is labeled as their home location. App usage variables were added by classifying apps based on their category in the Google Play Store (Google Play Store Team, 2020).

We used data from the ESM questionnaires to create social interaction variables. We created variables that indicate the duration that a participant spent in a specific social interaction. For example, the total minutes that someone spent in face-to-face interactions or in online interactions. Furthermore, participants could select the purpose or topic of their interactions, such as “catching up” or “joking around”. To define the content of an interaction we used Hall's (2018b) definitions of “communication episodes”, which describes four different types of content and behavior of an interaction (i.e., striving behavior, mundane and maintenance behavior, work or school talk, and negative content). Additionally, we created variables about the last interaction a person had before the scheduled ESM questionnaire and how long ago this interaction was. This means that the most recent interaction may have taken place before the chosen level of aggregation. For example, if the level of aggregation was 3 h, the last interaction could have occurred 4 h before the ESM questionnaire was filled out. We exclude interactions that occurred during the lag of 30/60 min to be able to predict mood in advance.

We created variables to indicate various interaction partners by matching the nicknames from the egocentric network with data from the ESM period. This allowed us to examine the impact of the characteristics of interaction partners on mood (Stadel et al., 2022). For example, we created a variable that indicated the length of time (in minutes) that someone spent in an interaction with a friend or a family member, as well as a variable that reflected the level of closeness with an interaction partner during a given period. During periods where multiple interaction partners were present, we calculated the majority or mean value for the given variable to provide an overall measure of relationship characteristics. For example, if participants had an interaction with three friends and one stranger, the interaction would count as having an interaction with friends and not with strangers. Unless indicated otherwise, all questions were measured on an 11-point Likert scale (see Table 1).

To prepare social interaction predictors for analyses, we employed several preprocessing steps using the *caret* package. First, we used the centering and scaling function to normalize continuous variables (Kuhn, 2008). Next, we transformed categorical variables into dummy variables. We excluded any predictor with zero or near-zero variance. Passive smartphone measures were considered missing if no data was recorded for 24 h. We imputed missing data based on the k-nearest neighbors in the training set.

Participants had the option to exclude individuals from their egocentric network if they did not have frequent contact with them or if they were not considered relevant. Additionally, “stranger(s)” were not included in the egocentric network. We assumed that those people were not close to the participant, the participant did not discuss personal issues with them, and they did not provide emotional and practical support. Therefore, scores on those variables were given a value of zero. We imputed selected social interaction variables (e.g., “Spending time with this person costs energy”, “Spending time with this person gives energy”, “I can be myself with this person”) as we had no clear hypothesis of how the participant would have rated these variables.

### Prediction Model

#### Prediction Algorithm

We used random forest models to predict positive and negative affect. Random forest models are computationally efficient models that can capture nonlinear relationships and are commonly used in digital phenotyping studies to predict mental health outcomes (e.g., for a review see Benoit et al., 2020). Random forest models combine several decision trees to make predictions (Breiman, 2001). A decision tree splits the outcome variable into different subsets based on the predictor variables. The final nodes of the tree correspond to the predicted outcome value. A disadvantage of decision trees is that they are not very robust, as small changes in the data can cause large changes in the final estimated tree (Hastie et al., 2009). To address this limitation, a random forest generates several random decision trees. More specifically, a number of bootstrapped observations and randomly selected variables are used to build these random decision trees. The final prediction is based on the average over all trees (Hastie et al., 2009). Random forest requires the tuning of hyperparameters, such as the number of randomly selected predictors and the number of random decision trees, those hyperparameters are chosen by applying cross-validation. The optimal number of randomly selected predictors was chosen by excluding the last observation in the training set. We chose between three to 18 randomly selected predictors in each tree. For the other hyperparameters (e.g., number of trees), we used the default values from the *caret* package (Kuhn, 2008).

We used the *caret* package to run the machine learning models. Analyses were conducted using R version 4.2.1 (2022-06-23). The code used to clean and analyze the data can be found in the following GitHub repository: https://github.com/AnnaLangener/CombiningMethods_MoodPredictions.

#### Model Validation

**Fig. 2:**
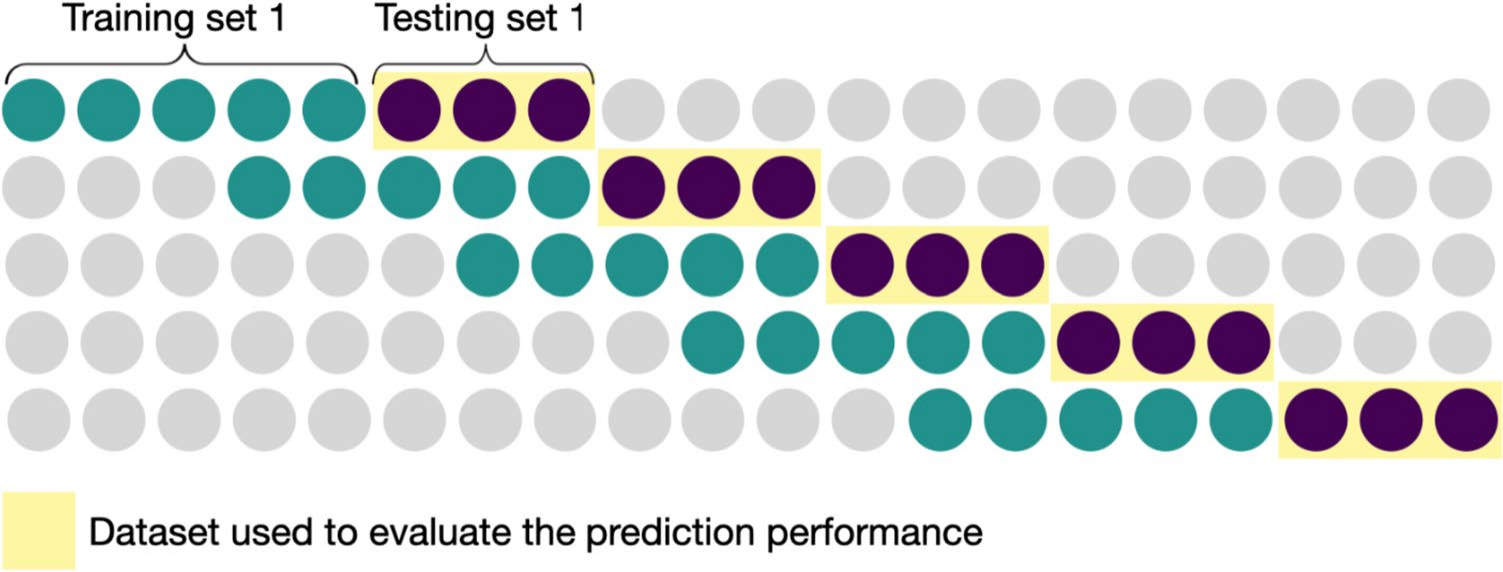
Example for moving window cross-validation set-up (window size: 5, horizon: 3). *Note* The Figure illustrates the setup for the moving window cross-validation. We depict a moving window size of five ESM measurements. This means that a model is constructed using only the most recent five observations to train the model (represented by the green circles in each row). The test set contains the next three observations (represented by purple circles). Thus, each of the three points gets predicted by utilizing the model that was trained in the training set. This process is repeated until the end of the dataset is reached. To estimate overall prediction performance, we combine all predictions made on the different test sets (as highlighted in yellow) (Color figure online)

Two main strategies exist for evaluating time-series prediction models, namely, fixed origin versus rolling origin evaluation (Hewamalage et al., 2022; Tashman, 2000). In the fixed origin the dataset gets split into two sets where, for example, the first 70% of observations is used for building a model and the last 30% for evaluation. In contrast, the rolling origin approach uses multiple training and evaluation sets to predict the next set of observations, repeating the process until the end of the dataset is reached (see Fig. 2 for an illustration).

A rolling origin evaluation has several advantages over a fixed origin approach. Rolling origin evaluation is considered more robust, less dependent on specific patterns, and it can recalibrate the model with new data (Tashman, 2000), and thus, is usually recommended (Hewamalage et al., 2022). The rolling origin evaluation setup is similar to another approach referred to as blocked cross-validation (Bulteel et al., 2018).

We expected that the window size would have an impact on the prediction performance (Gama et al., 2014). Therefore, we tested three rolling window sizes ranging from around 3 days of data to 6 days of data (15, 20, and 30 ESM measurements). We opted for relatively short moving window sizes in order to capture changes in the association between social behavior and mood. This decision was also influenced by our relatively small number of ESM measurements. Accordingly, we wanted to ensure that an adequate number of data points remained for inclusion in the test set. Each test set contains three data points (also called prediction horizon), which is roughly half a day of data. We combined all predictions made into a single test set to evaluate the prediction performance. Figure 2 illustrates the rolling origin approach with a window size of five data points and a test set of three data points.

To evaluate the prediction performance, we calculated the coefficient of determination (*R*^2^; which was defined as one minus the fraction between the sum of squared differences between the outcome and the model predictions and the sum of squared differences between the outcome and an intercept-only model), the mean absolute error (MAE), and the mean absolute percentage error (MAPE) in the test set. We decided to use multiple measures to evaluate the prediction performance because *R*^2^ is a relative measure of fit (as it depends on the total variance) and does not provide any information about the overall accuracy of the prediction model. In contrast, measures such as the MAE can be used to evaluate the prediction error and thus the accuracy of the model (Rocca & Yarkoni, 2021). We additionally report the MAPE since it makes the evaluation of prediction models comparable with each other. Both MAE and MAPE have been reported in previous papers that used passive smartphone measures for prediction (e.g., Jacobson & Chung, 2020; Shah et al., 2021). Lastly, we investigated whether our model performs better than a baseline model, which uses the mean in each training set as the primary predictor. Our model must perform better than this baseline model to be considered acceptable.

### Variable Importance

We assessed the variable importance for the best-performing model for each participant, by calculating the (mean) Shapley value of each predictor in the train set^1^ (similar to Jacobson & Bhattacharya, 2022; Shah et al., 2021). Shapley values are based on coalitional game theory and assume that each variable is a “player” in the prediction task and the prediction made is the “payout” of a game (Molnar, 2018; Štrumbelj & Kononenko, 2014). In other words, the “game” refers to predicting a single data point, and the “gain” is the difference between the actual and mean prediction. The variables, or “players”, work together to receive this gain by forming coalitions. Importantly, Shapley values measure a variable's contribution relative to the mean prediction, without directly assessing the actual prediction performance.

To determine the Shapley values we used the *iml* package to calculate the marginal contribution of a variable across all possible coalitions of variables (Molnar, 2018). Variables that are not in a coalition are replaced with random values from the dataset, which means that they should not be related to the outcome variable anymore. The prediction is then calculated both with and without the variable of interest, resulting in the marginal contribution of a variable (Molnar, 2018). To get an overall variable importance score we computed the mean absolute Shapley value across all data points as in previous research (e.g., Jacobson & Bhattacharya, 2022; Shah et al., 2021).

### Deviations from Preregistration and Robustness Checks

Based on the collected ESM data we derived several variables (see Table 1). We used Hall's (2018b) definitions of so-called communication episodes to describe the content of an interaction (i.e., striving behavior, mundane and maintenance behavior, work or school talk, and negative content). In our preregistration, we included the content “catching up” in both the “mundane and maintenance behavior” and “striving behavior” communication episodes. However, “catching up” is only applicable to the “striving behavior” episode (Hall, 2018b). We have corrected this error in our data analysis.

During the study, participants recorded the start and end times of their social interactions, while the timestamp of when they completed the ESM questionnaire was recorded automatically. For instance, a participant may have reported an interaction starting at 11:00 and ending at 13:00, with the ESM questionnaire completed at 13:10 on 21.11.22. We manually added the date to the start and end times of each interaction to create variables. This led to some inconsistencies, for example, participants reported having an interaction from 20:15 to 00:00 and handing in the survey at 21:24. We dealt with inconsistencies between automatic timestamp and participant self-report by manually adjusting the end time of an interaction if we believed the participant had made an error in logging the time (N = 7 out of 1313 observations). For example, we replaced the self-reported end time (e.g., 00:00) with the survey completion time (e.g., 21:24).

To ensure the robustness of our data analysis, we conducted analyses using the original data (except for one interaction that lasted for 0 min). The results from this analysis are reported in the Supplementary Materials and discussed in the section *Robustness Checks*. Notably, the overall results exhibited minimal changes when comparing the different analyses. All changes made to the data can be found in the following R script: https://github.com/AnnaLangener/CombiningMethods_MoodPredictions/blob/master/data_matching.R.

Using different moving window sizes in the cross-validation set-up led to different test set lengths. For instance, let us consider a scenario where a participant fills out 100 ESM questionnaires. If we select a training size of 15 data points, the test set across all moving windows will include a total of 85 data points. This is because the first 15 data points are necessary to train the first model, while the other data points can be used to test the model. In contrast, when a moving window size of 30 data points is used, the test set will only include 70 data points, as more data points are utilized for training the model. Hence, the test sets will have different sizes. To investigate whether different test set lengths affect the prediction performance, we recalculated the performance measures using the same test set length, meaning that we only used data after the 30th ESM measurement to calculate the prediction performance. We completed multiple robustness checks for our prediction models.

### Overview of All Models

Different models were compared to evaluate the additive predictive value of ESM, digital phenotyping, and egocentric networks in predicting positive and negative affect (similar to Sano et al., 2018; see Table 2). The predictors for each model varied. In the first model, data from all measures were used (i.e., digital phenotyping, ESM, egocentric networks). In the second model, only digital phenotyping data was used, and in the third model only ESM data was used. In the last model, only data from the egocentric network, combined with the names of interaction partners that were assessed during the ESM period, were used. We will refer to this model from now on as using only egocentric network variables. We further varied the level of aggregation for the predictors and the cross-validation rolling window size.

**Table 2:** Overview of different models

Description	Overview
Prediction outcome	∙ Negative affect ∙ Positive affect
Level of aggregation for predictors	∙ 3 h before the scheduled questionnaire ∙ 6 h before the scheduled questionnaire ∙ 24 h before the scheduled questionnaire
Included predictors	∙ All predictors ∙ Only digital phenotyping ∙ Only ESM ∙ Only egocentric network (combined with [nick]names assessed via ESM)
Prediction lag	∙ 60 min ∙ 30 min *(robustness check)*
Cross validation rolling window size	∙ 15 ∙ 20 ∙ 30
Data cleaning	∙ Manually adjusting the end time of seven interactions ∙ Manually adjusting the end time of one interaction *(robustness check)*
Test set	∙ Test set length equal (across different moving window sizes) ∙ Test length unequal *(robustness check)*

We completed three robustness checks. First, we varied the time window used prior to the mood assessment in which predictors were aggregated (see Fig. 1). We used a 30 min lag instead of a 60 min lag. Second, we tested the impact of our preprocessing steps to clean the timestamp data (i.e., manually adjusting the end time of one interaction vs. seven interactions). Lastly, we checked the impact of using an equal test set length for different moving window sizes.

## Results

### Descriptive

Participants completed on average n = 119.4 (SD = 8.44, min = 108, max = 135) scheduled ESM questionnaires. These assessments were used to measure participants’ positive and negative affect throughout the study. Participants had an average positive affect of M = 6.42 (SD = 1.89), and an average negative affect of M = 2.58 (SD = 2.12). Additionally, participants logged on average 77.73 (SD = 42.91, min = 8, max = 162) social interactions throughout the study (see Table 3). We created line plots to illustrate how each predictor aggregated on different scales changes over time for each participant (see Supplementary Material).

**Table 3:** Total ESM measures per participant, mean positive affect, mean negative affect, and the total number of logged interactions

Participant	N	Positive affect M (SD)	Negative affect M (SD)	Total interactions N
1	119	5.09 (2.45)	3.07 (1.97)	35
2	114	7.82 (1.67)	0.66 (0.99)	54
3	108	6.13 (2.14)	5.46 (2.15)	8
4	117	6.12 (1.8)	2.38 (1.42)	98
5	128	7.23 (0.96)	1.74 (1.2)	100
6	135	7.03 (1.73)	2.02 (1.34)	78
7	124	5.02 (1.52)	4.6 (1.36)	108
8	115	6.23 (1.77)	0.54 (1.04)	60
9	108	6.44 (1.65)	3.5 (1.84)	107
10	127	5.9 (1.21)	3.94 (1.15)	45
11	118	7.62 (0.99)	0.68 (0.82)	162

### Overall Prediction Performance Using Different Time Scales

#### Positive Affect

We first evaluated the performance of different models predicting positive affect. First, we varied the predictors included in each model: data from all measures, digital phenotyping only, ESM only, or egocentric networks data only. Second, we constructed models at different levels of aggregation. Third, we varied the number of observations used to train the model in the cross validation set-up (i.e., 15, 20, 30 observations; see Table 2 for an overview).

**Fig. 3:**
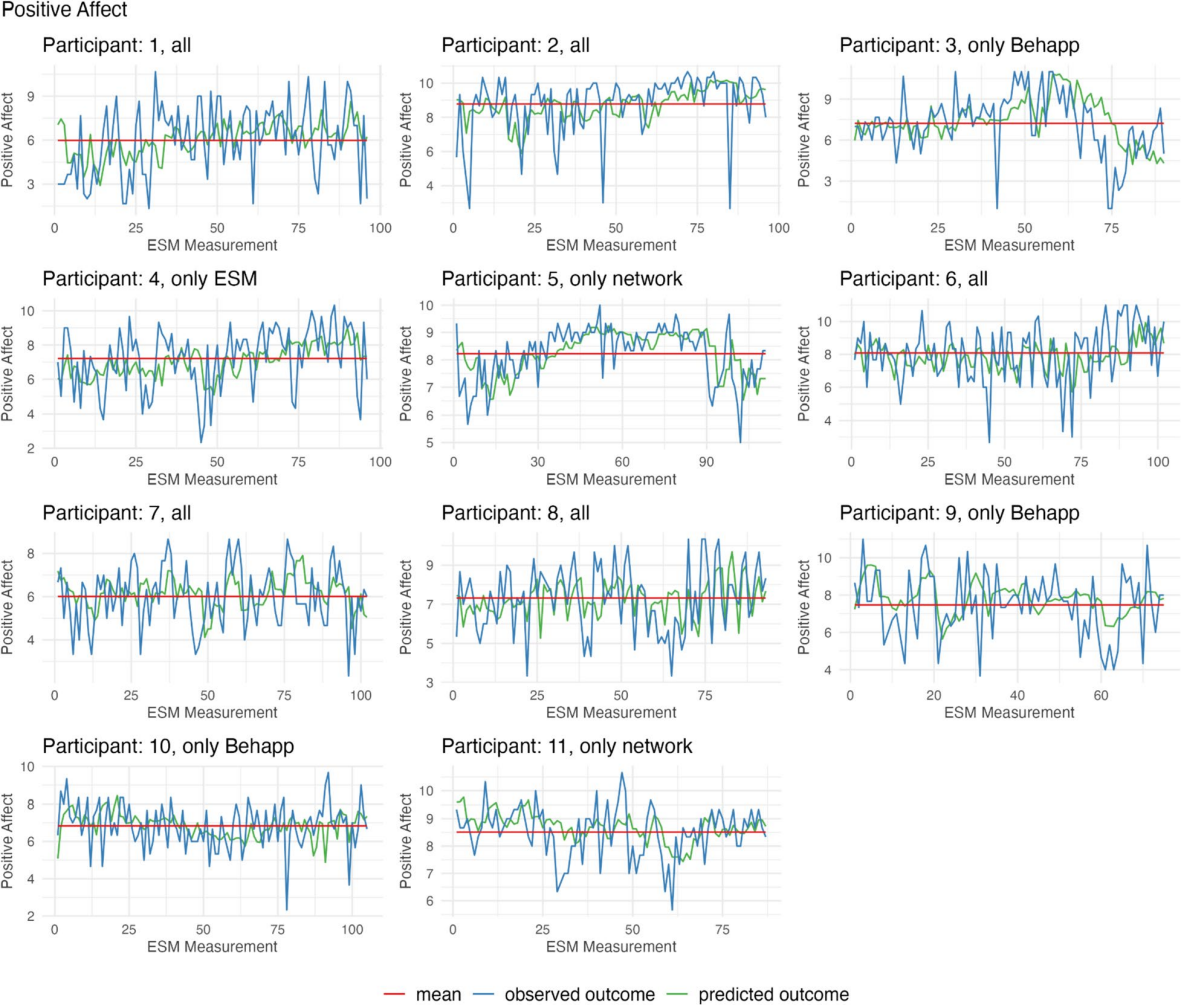
Predicted and observed values of positive affect for each participant. *Note.* The Figure displays positive affect scores measured at each scheduled ESM questionnaire. The green line indicates the predicted positive affect score from the random forest model with the highest prediction accuracy (based on *R*^2^, using individualized models with a 1 h lag between the predictors and the assessment of positive affect). The blue line shows the observed positive affect score, while the red line represents the mean value of positive affect (Color figure online)

We examined the accuracy of each model in predicting positive affect. We start by describing the overall prediction performance based on the best-performing model (i.e., model with the highest *R*^2^ for each participant). Figure 3 displays the predicted and observed values of positive affect measured during the scheduled ESM questionnaires for each participant, providing an overview of the model’s performance. Table 4 summarizes the accuracy of the best-performing model per participant.

**Table 4:** Results for the best-performing model to predict positive affect (lag 60 min) including the standard deviation and minimum value using different window sizes and levels of aggregation

	Predictors	Coefficient of determination	Correlation	Mean absolute error	Mean absolute percentage error	Window	Aggregation
1	All*	0.04 (min = − 0.14, SD = 0.06)	.27 (min = .13, SD = .05)	1.92 (max = 2.08, SD = 0.05)	47.89% (max = 49.21%, SD = 1.33%)	20	6
2	All*	0.03 (min = − 0.28, SD = 0.09)	.28 (min = .08, SD = .06)	1.19 (max = 1.32, SD = 0.06)	19.56% (max = 20.71%, SD = 1.4%)	15	24
3	Only Behapp*	0.09 (min = − 0.12, SD = 0.07)	.41 (min = .2, SD = .09)	1.52 (max = 1.83, SD = 0.11)	42.6% (max = 53.45%, SD = 5.01%)	15	3
4	Only ESM*	0.07 (min = − 0.15, SD = 0.09)	.32 (min = .14, SD = .08)	1.44 (max = 1.58, SD = 0.05)	24.17% (max = 26.61%, SD = 0.98%)	20	6
5	Only Network*	0.21 (min = − 0.17, SD = 0.13)	.52 (min = .33, SD = .07)	0.66 (max = 0.7, SD = 0.03)	8.69% (max = 9.19%, SD = 0.48%)	15	24
6	All*	0.07 (min = − 0.24, SD = 0.11)	.33 (min = .05, SD = .1)	1.34 (max = 1.56, SD = 0.08)	18.75% (max = 22.88%, SD = 1.4%)	30	6
7	All*	0.01 (min = − 0.32, SD = 0.1)	.27 (min = .18, SD = .14)	1.13 (max = 1.34, SD = 0.06)	22.19% (max = 25.27%, SD = 0.93%)	20	24
8	All*	0.19 (min = − 0.03, SD = 0.08)	.45 (min = .22, SD = .09)	1.21 (max = 1.37, SD = 0.05)	18.24% (max = 20.53%, SD = 0.88%)	20	6
9	Only Behapp*	0.03 (min = − 0.26, SD = 0.12)	.3 (min = − .14, SD = .19)	1.4 (max = 1.56, SD = 0.08)	22.03% (max = 23.85%, SD = 1.17%)	30	24
10	Only Behapp	− 0.05 (min = − 0.23, SD = 0.06)	.21 (min = − .02, SD = .07)	0.95 (max = 1.02, SD = 0.03)	15.61% (max = 16.72%, SD = 0.41%)	20	6
11	Only Network	0.01 (min = − 0.59, SD = 0.21)	.31 (min = − .13, SD = .17)	0.73 (max = 0.93, SD = 0.07)	9.13% (max = 11.28%, SD = 0.84%)	30	24

The table shows the prediction performance for the best-performing model predicting positive affect. We varied the window size of the cross-validation set-up and the level of aggregation of the predictor variables. The models tested included using all predictors, only ESM, only Behapp, or only the egocentric network variables. The star indicates that the model performed better than a model using the mean in the training set as a predictor (using the same rolling window size), while other models either performed equally well or worse. In brackets, we show the standard deviation and worst-performing model across different moving window sizes and levels of aggregation, using the same predictor variables

Participant 3 and Participant 10 had several instances where a model could not make a prediction when using all predictors, as there was insufficient data to impute the missing ESM and egocentric network predictors. In these cases, we considered the model to be performing worse than a model that could generate predictions for all data points with a more limited set of variables.

For the majority of participants (9 out of 11) the best-performing model predictions outperformed predicting the mean in the training set (using the same window size, see Supplementary Material for the results). However, *R*^2^ values were low with an average *R*^2^ over all participants of 0.06 (min = − 0.05, max = 0.21, SD = 0.08), indicating that the model was not able to explain a substantial fraction of variance in the data (note that negative *R*^2^ can occur because our measure of *R*^2^ is relative to an intercept-only model; a negative *R*^2^ implies that our fitted model performs worse than an intercept only model). The models achieved a moderate average correlation of 0.33 (min = 0.21, max = 0.52, SD = 0.09) between predictions and outcomes and an average MAE of 1.23 (min = 0.66, max = 1.92, SD = 0.36). This means that, on average, the prediction differed by 1.23 points from the observed value, which was measured on an 11-point scale. This resulted in an average MAPE of 22.62% (min = 8.69%, max = 47.89%, SD = 12.29%).

We explored different levels of aggregation and moving window sizes to determine their impact on the model performance. Results demonstrated that a time frame of 6 h or 24 h produced the most accuracy results for the majority of participants (see Table 4; 5 out of 11 each). There was one participant whose model demonstrated the best performance when aggregated at 3 h. The optimal moving window size in the cross validation set-up also varied, with 15 being the best for three participants, 20 for five participants, and 30 for three participants. Overall, the performance within a participant was not very robust using different levels of aggregation and moving window sizes as indicated by the relatively large standard deviation of performance measures across different time scales (see Table 4).

To summarize, the overall prediction performance for positive affect was weak and varied per participant. Moreover, the prediction performance varied between different time scales that were used to summarize the predictor variables and to train the prediction model.

#### Negative Affect

**Fig. 4:**
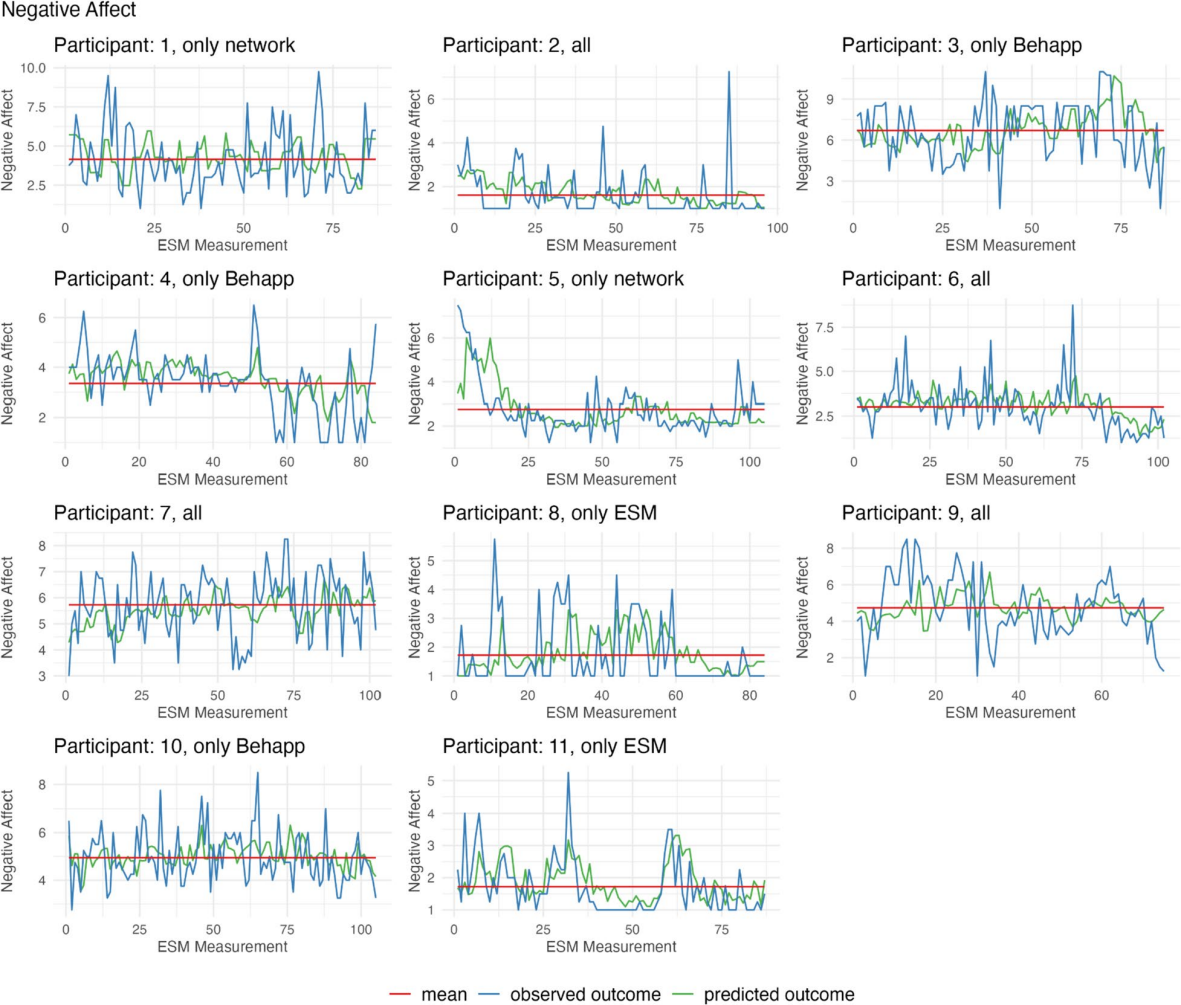
Predicted and observed values of negative affect for each participant. *Note.* The Figure shows negative affect scores measured at each scheduled ESM questionnaire. The green line indicates the predicted negative affect score from the random forest model with the highest prediction accuracy, based on *R*^2^, using individualized models with a 1 h lag between the predictors and the assessment of positive affect. The blue line shows the observed negative affect score, while the red line represents the mean value of negative affect (Color figure online)

Next, we examined how well we were able to predict negative affect. Figure 4 displays the predicted and observed values of negative affect. Table 5 shows the accuracy of the best-performing model. If the model was unable to make any prediction (as was the case for Participant 3 and Participant 10), the model was considered as performing worse than a model capable of making predictions for all data points.

**Table 5:** Results for the best-performing model to predict negative affect (lag 60 min) including the standard deviation and minimum value using different window sizes and levels of aggregation

	Predictors	Coefficient of determination	Correlation	Mean absolute error	Mean absolute percentage error	Window	Aggregation
1	Only Network	− 0.08 (min = − 0.33, SD = 0.09)	.16 (min = .01, SD = .06)	1.67 (max = 1.82, SD = 0.09)	51.15% (max = 55.18%, SD = 3.27%)	30	6
2	All*	− 0.02 (min = − 0.31, SD = 0.09)	.23 (min = − .1, SD = .1)	0.67 (max = 0.76, SD = 0.02)	45.31% (max = 52.16%, SD = 2.39%)	15	24
3	Only Behapp*	− 0.07 (min = − 0.27, SD = 0.08)	.24 (min = 0, SD = .08)	1.86 (max = 2.04, SD = 0.07)	39.56% (max = 45.43%, SD = 2.88%)	20	24
4	Only Behapp*	0.16 (min = − 0.12, SD = 0.08)	.43 (min = .26, SD = .06)	0.8 (max = 0.93, SD = 0.04)	38.69% (max = 43.91%, SD = 2.11%)	30	6
5	Only Network*	0.21 (min = − 0.74, SD = 0.71)	.53 (min = − .07, SD = .2)	0.71 (max = 0.89, SD = 0.08)	25.43% (max = 30.12%, SD = 1.5%)	20	24
6	All*	0.21 (min = − 0.04, SD = 0.07)	.47 (min = .27, SD = .06)	0.81 (max = 0.92, SD = 0.04)	32.13% (max = 35.34%, SD = 1.45%)	30	6
7	All*	0.02 (min = − 0.23, SD = 0.08)	.27 (min = .03, SD = .07)	0.97 (max = 1.19, SD = 0.06)	18.43% (max = 23.64%, SD = 1.73%)	20	24
8	Only ESM*	0.02 (min = − 0.11, SD = 0.05)	.31 (min = .22, SD = .04)	0.85 (max = 0.89, SD = 0.04)	55.54% (max = 57.42%, SD = 1.94%)	30	24
9	All*	− 0.04 (min = − 0.22, SD = 0.06)	.13 (min = − .01, SD = .06)	1.41 (max = 1.51, SD = 0.05)	44.24% (max = 50.69%, SD = 3.13%)	30	24
10	Only Behapp*	0.01 (min = − 0.21, SD = 0.07)	.24 (min = .05, SD = .07)	0.86 (max = 0.95, SD = 0.03)	18.16% (max = 19.93%, SD = 0.63%)	20	6
11	Only ESM*	0.3 (min = − 0.22, SD = 0.17)	.59 (min = .17, SD = .14)	0.54 (max = 0.73, SD = 0.07)	35.52% (max = 47.19%, SD = 5.15%)	30	24

The table indicates the prediction performance for the best-performing model predicting negative affect. We used different moving window sizes in the cross-validation set-up and varied the level of aggregation of the predictor variables. The models tested included using all predictors, only ESM, only Behapp, or only the egocentric network variables. The star indicates that the model performed better than using the mean from the training set as the prediction, while other models either performed equally well or worse. In the brackets, we show the standard deviation and worst-performing model across different moving window sizes and levels of aggregation, using the same predictor variables

Similar to the positive affect models, for most participants (10/11) trained models showed better predictions than using only the mean from the training set. Nevertheless, the *R*^2^ values were low, with a mean of 0.06 (min = − 0.08, max = 0.3, SD = 0.13), indicating that the variance in the observed data was not well explained by the predictions. The correlation was moderate, with a mean of 0.33 (min = 0.13, max = 0.59, SD = 0.15). The MAE was slightly lower than when predicting positive affect, with a mean of 1.01 (min = 0.54, max = 1.86, SD = 0.43). However, the MAPE was higher, with an average of 36.74% (min = 18.16%, max = 55.54%, SD = 12.38%).

In line with results from the positive affect models, participants had varying optimal levels of aggregation and moving window sizes for predicting negative affect. The optimal time frame of aggregation was 6 h for four participants and 24 h for seven participants. The moving window size with the highest accuracy was 15 for one participant, 20 for four participants, and 30 for six participants. The results show varying levels of prediction performance stability when changing the moving window size or level of aggregation.

Overall, the prediction performance for negative affect was weak and also varied per participant. Similar to results from the positive affect models, prediction performance varied between different time scales that were used to summarize the predictor variables and to train the prediction model.

### How Much Does Each Method Add to Predicting Mood

We further investigated which combination of variables led to the highest prediction performance for positive and negative affect. Four different sets of predictors were examined, including ESM, passive smartphone measures, egocentric networks, or a combination of all. We start by investigating the set of predictors that performed best per participant.

Results demonstrated that the best set of predictors varied across participants (see Table 4). Including all predictors improved prediction accuracy of positive affect for five participants. Using only egocentric network variables resulted in the highest performance for two participants. For another three participants, using only Behapp outperformed the other models. However, two of these three participants were Participant 3 and Participant 10, who both had a low number of logged interactions, resulting in missing values in the predicted data points. Lastly, for one participant, using only ESM achieved the best results.

**Fig. 5:**
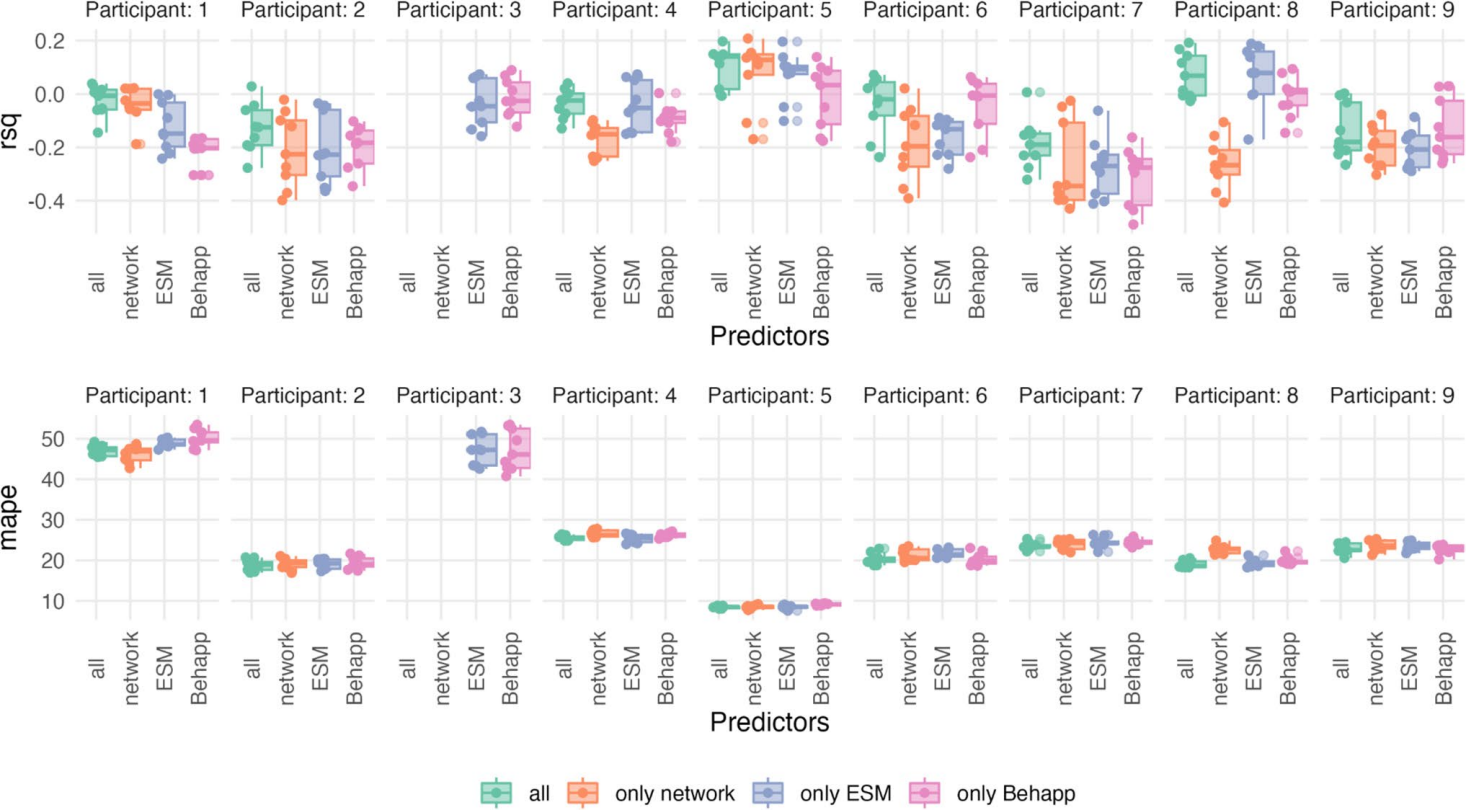
Distribution of *R*^2^ and the MAPE over different models predicting positive affect. *Note*. This Figure illustrates the variation of prediction performance for positive affect across different models (see Table 2) and participants. The top row shows the distribution of *R*^2^ values using boxplots, while the bottom row shows the MAPE. Both plots include the jittered raw data points next to the boxplot. Only participants for whom the best-performing model was better than using the mean in the training set as prediction and had a positive *R*^2^ are displayed

The best model's prediction performance was often similar to the next best-performing model (using a different moving window size, level of aggregation, or set of predictors). Therefore, we also investigated how often different sets of predictors lead to an acceptable prediction performance over different time scales and participants. We define an acceptable prediction as one that performs better than the mean prediction from each training set (i.e., higher *R*^2^ and lower MAE) and obtains a positive *R*^2^ value. Figure 5 shows the distribution of *R*^2^ and MAPE for the participants whose best-performing model had an acceptable prediction. We observe that there was variability between which predictors performed best between participants, but that models that performed well were often close to each other.

Among the selected participants, using all predictors resulted in acceptable predictions for 22.2% of the models (n = 22). This means that out of all 99 models (3 levels of aggregation × 3 moving window sizes × 11 participants) across all participants in which all predictors were used, 22 models gave predictions that outperformed an intercept-only model and the mean from each training set. This was followed by using only ESM (13.1%, n = 13) and only Behapp data (12.1%, n = 12). Using only variables from the egocentric network performed worse for the selected participants (10.1%, n = 10).

**Fig. 6:**
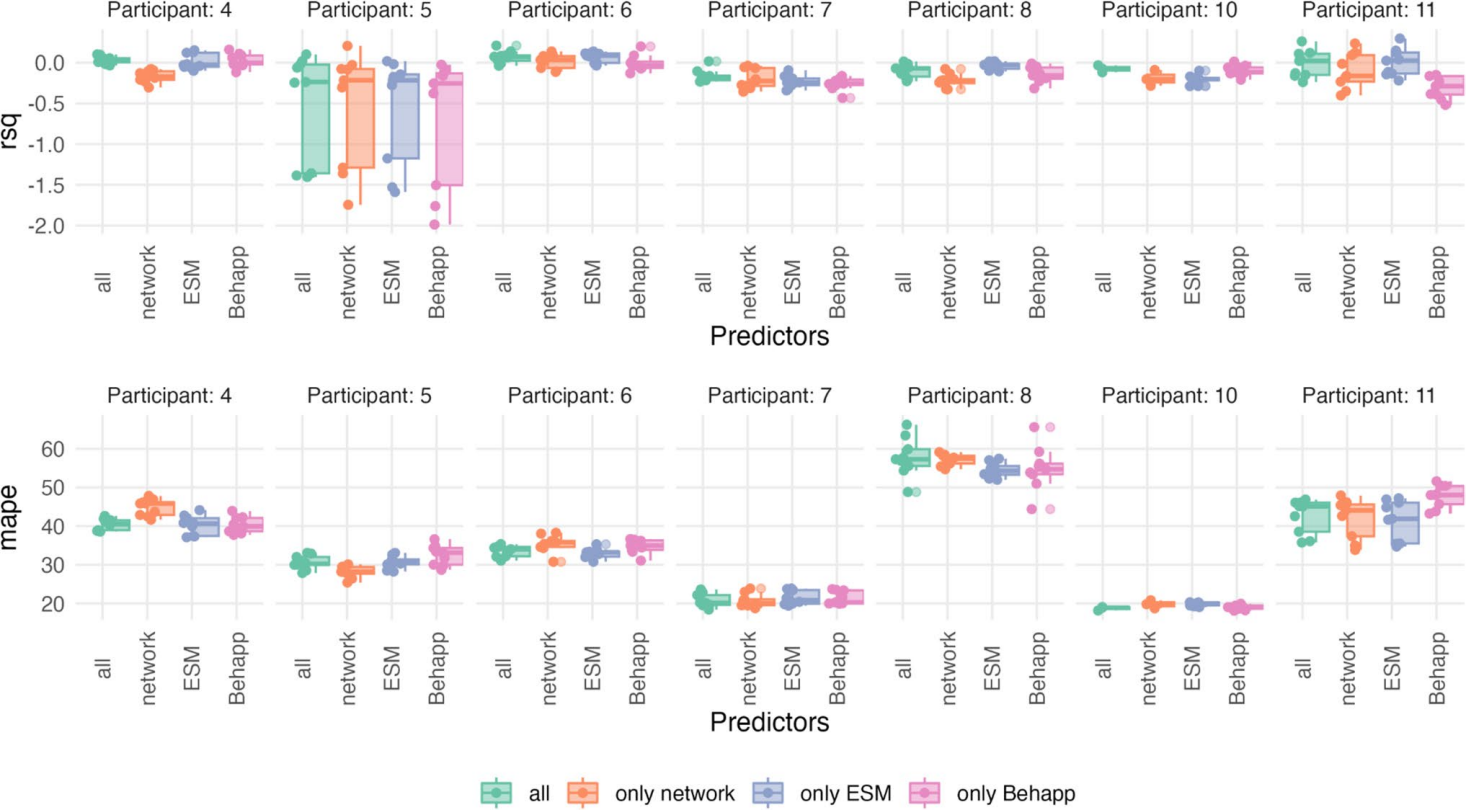
Distribution of *R*^2^ and the MAPE Over Different Models Predicting Negative Affect. *Note.* This Figure shows how prediction performance for negative affect varies across different models (see Table 2) and participants. The top row displays *R*^2^ values using boxplots, while the bottom row shows MAPE, including jittered raw data points in both plots. Only participants whose best performing model performed better than the mean in the training set and had a positive *R*^2^ are shown

We observed similar results for negative affect models. Including all predictors improved model accuracy for four participants; whereas using only Behapp outperformed other models for three participants (including Participants 3 and Participant 10). For two participants, using only egocentric network variables achieved the best performance, and for another two participants, using only ESM produced the best predictions. Figure 6 shows that, similarly to predicting positive affect, there was variability between which predictors performed best across participants (whose best-performing model made an acceptable prediction), but that good-performing models were often close to each other.

Using all predictors yielded the best results and led to an acceptable prediction in 14.1% of the cases (n = 14). The second-best model used only ESM variables (12.1%, n = 12), followed by using only network variables (6.1%, n = 6). Among the selected participants, only in three cases using only Behapp data produced acceptable predictions (3%, n = 3).

### Robustness Check

To ensure the robustness of our results, we used four different datasets summarized inTable 2.^2^ First, we examined whether the *R*^2^ and MAPE were similar across the different datasets. We also checked whether the best predictors included in a model were robust by recalculating how often different sets of predictors (i.e., using all predictors, only egocentric network, only Behapp, or only ESM) resulted in acceptable predictions. A full overview of the robustness checks can be found in the Supplementary Material (section *Robustness Checks*). In this section, we provide a brief summary of the results.

For positive affect, we observed a strong association between the *R*^2^ values and the MAPE across the different datasets (all r > 0.97). Across all datasets, using all predictors performed best, followed by using only Behapp and only ESM. Using only egocentric network variables performed worst. This suggests that when predicting positive affect, the results are robust across different datasets.

For negative affect, the association between *R*^2^ values across different datasets revealed a high correlation for three out of the four datasets (all r > 0.98). However, a notable difference emerged when utilizing a dataset that included an equal test set length for different moving window sizes (r = 0.7). A similar pattern emerged when examining which predictors performed best. Upon closer examination, we found that this pattern was primarily due to one participant (Participant 5, see the Supplementary Material Fig. 51 for more information) who had large values of negative affect at the beginning of the study. By making the test length equal across different moving window sizes, we excluded those data points, leading to unstable results.

### Variable Importance

We computed Shapley values to gain insights into the importance of different variables in predicting mood. This analysis was conducted on the best performing model for each participant, considering all predictors. However, it is important to note that we only computed Shapley values for participants whose best performing model using all predictors had a higher predictive accuracy compared to the baseline model, as none of the variables in models with poor predictive accuracy will be important.

**Fig. 7:**
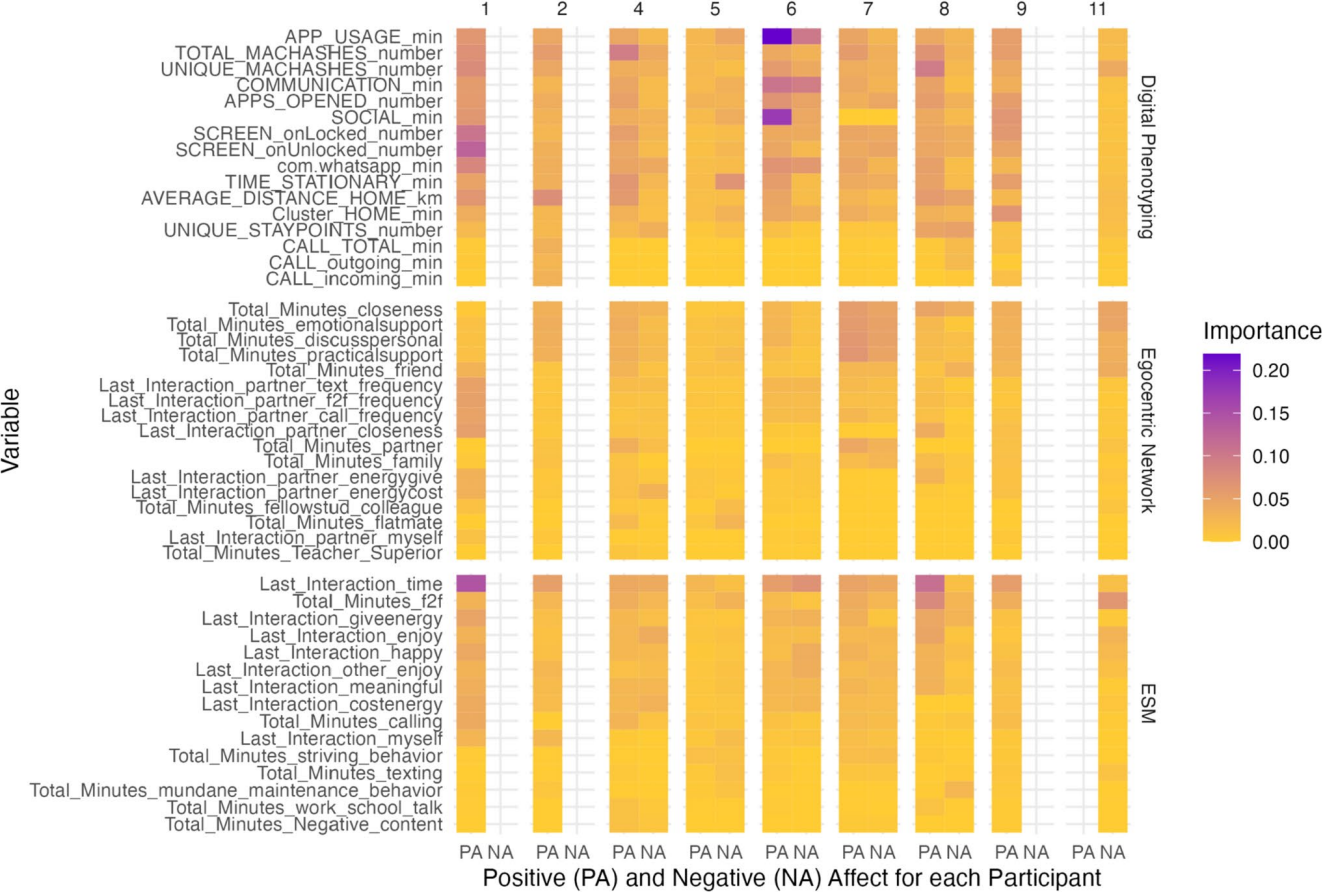
Shapley values.* Note* This Figure shows the variable importance (mean absolute Shapley value) for the best performing individualized prediction model for positive and negative affect using all predictors for each participant. We only present the results of participants whose prediction performance was better than that of the baseline model. The x-axis represents the participant ID and the outcome variable, which can be either positive affect (PA) or negative affect (NA). The y-axis displays the different variables included in the analysis. The color illustrates the variable importance. The variables are sorted based on the mean importance value within each category across participants, from highest to lowest (Color figure online)

Figure 7 illustrates the overall variable importance for each variable and participant. As an example, the highest variable importance was found for respondent 6 for positive affect: The total duration of app usage had an average mean absolute Shapley value of 0.22. This indicates that, on average, app usage contributed to a difference of 0.22 compared to the mean prediction across different coalitions and data points for this participant. Some values are zero, for example, the total minutes spent with a teacher or superior showed a value of zero for respondents 1, 2, 7, 8, and 9 for positive affect. This implies that this particular variable had no impact on the prediction, most likely because it was often removed from the model due to its (near-)zero variance. Moreover, our results suggest that certain variables are of little importance across participants. For example, text messaging was not a strong predictor of mood. Additionally, the content of conversations (e.g., about work or school versus small talk), seemed to have little impact (although this pattern could be due to limited variation as participants did not record many interactions throughout the day). Phone calls recorded via Behapp also appeared relatively unimportant for predicting mood.

## Discussion

### Insights and Reflections on Clinical Utility

In this study, we examined the extent to which various aspects of social context predicted mood. To capture the social context, we integrated data from digital phenotyping, ESM, and egocentric networks. Our three primary objectives were to: (1) to assess the accuracy of mood prediction using different methods that measured distinct aspects of the social context over different time scales, (2) to investigate the extent to which the chosen time scale impacted prediction performance, and (3) to explore whether a combination of different methods increases predictive accuracy.

#### Low Overall Predictive Accuracy and Variation Among Participants

Our findings indicated that the overall predictive accuracy is low, as reflected by an average *R*^2^ value of 0.06 for positive affect and negative affect. Additionally, the average MAE for positive affect is 1.23 points, and the average MAPE is 22.62%. Similarly, for negative affect, the average MAE is 1.01, and the average MAPE is 36.74%. These results suggest that our machine learning models are unable to explain a large portion of the variance. Furthermore, the overall predictive ability varied among participants. Some participants had a moderate predictive accuracy, with the highest *R*^2^ values of 0.21 and 0.3 for positive affect and negative affect, respectively. In contrast, for other participants, the predictions were worse than those of a baseline model. In practice, it will thus be challenging to determine in advance which participants’ personalized models will have acceptable predictive accuracy and can be used for personalized treatment.

Our results are similar to the findings of Asselbergs et al. (2016) who aimed to predict day–to–day mood using solely passive smartphone measures and individualized prediction models. Their goal was to replicate the research conducted by LiKamWa et al. (2013), who were the first to investigate the potential of predicting mood based on passive measures. Contrary to our results, LiKamWa et al. (2013) found a high predictive accuracy of 93% of the self-reported mood within a tolerated error margin of 0.5 around the observed scores. The findings of Asselbergs et al. (2016), however, were less promising, as their predictions were worse compared to a simple benchmark model, which is in line with our low predictive accuracy. Several factors may have contributed to these results. LiKamWa et al. (2013) conducted their study over a duration of 60 days, while both our study and that of Asselbergs et al. (2016) had considerably shorter durations of 28 and 42 days, respectively, which could have affected predictive accuracy (Asselbergs et al., 2016). Additionally, our study focused on a different population, consisting of Dutch students, while LiKamWa et al. (2013) conducted their research with Chinese students. These differences in population could also have influenced the predictive accuracy of the models.

#### Variation of Predictive Accuracy Across Time Scales

We observed that the chosen time scale for aggregating and analyzing the data influenced the prediction performance, leading to considerable variation in the predictive accuracy across different models. Generally, it appears that longer aggregation windows (e.g., 6 and 24 h) yielded superior results compared to shorter time windows (e.g., 3 h). One possible explanation for this could be that the participants did not interact frequently throughout the day; thus, longer time windows (e.g., 6 and 24 h) were able to capture infrequent, but impactful, social interactions.

A recent study investigated whether passive smartphone measures predicted negative affect using data from 50 participants who reported their negative affect 10 times a day (Niemeijer et al., 2022). Their aim was to predict the average negative affect of the next day using different machine learning models and they conducted a multiverse analysis to explore the impact of various preprocessing and analysis choices for each sensor on their results. Consistent with our own findings, this study exhibit a low predictive accuracy for negative affect, with the best model achieving an *R*^2^ value of 0.10 in the test set (Niemeijer et al., 2022).

Similar to our results, the multiverse analysis conducted by Niemeijer et al. (2022) revealed that different choices in methodology led to varying results in predictive performance. In practical applications, it is crucial to determine the appropriate preprocessing steps for each sensor and identify the optimal time scale in advance or to incorporate them into the cross-validation setup. Choosing preprocessing steps and the optimal time scale retrospectively can hinder real-time applications and potentially result in overfitting. Therefore, future research should focus on applying suitable methods to determine optimal preprocessing steps and time scales beforehand (Verachtert et al., 2022).

To summarize, in contrast to prior research that yielded promising results in mood prediction (LiKamWa et al., 2013), our findings align with studies indicating that it is challenging to accurately predict mood (Asselbergs et al., 2016; Niemeijer et al., 2022). Despite the combination of measures (i.e., passive smartphone measures, ESM, egocentric networks) the overall predictive accuracy for mood in our study remained low. Based on our results and following the findings by Asselbergs et al. (2016) and Niemeijer et al. (2022), the application of mood prediction models in real-world clinical settings is currently unlikely to be useful due to the low predictive accuracy and the observed variation among respondents and prediction models.

#### Different Parts of the Social Context Are Important to Predict Mood

Our results suggest that the optimal set of social context predictors varied among participants and that there is variation among participants in which specific variable was most important for predicting mood. This makes sense, considering that different parts of the social context may be more or less important for predicting the mood of different individuals. For instance, for people with few social interactions throughout the whole study period, passive measures, such as app usage, might be a better indicator of their mood than the total minutes they spent in interactions. However, we found that utilizing all predictors generally yielded the best predictions, which highlights that a combination of methods leads to higher predictive accuracy for the majority of participants and that different methods (digital phenotyping, ESM, egocentric networks) indeed tap into different aspects of the social context, even though the overall predictive accuracy was low.

We calculated Shapley values and found that phone calls recorded via Behapp are relatively unimportant for predicting mood. This is somewhat at odds with the finding that, for some participants (e.g., Participants 1 and 4), the total minutes spent calling recorded via ESM, is more important. This may be explained by the fact that Behapp only captures phone calls via the mobile network and no other forms of communication like (video) calls made via WhatsApp. Results support the integration of digital data sources to measure distinct aspects of social behavior that may otherwise be missed by relying solely on a single method.

### Limitations and Future Outlook

A unique strength of our study is the demonstrated impact of aggregation window and number of cross-validation observations on variability in predictive performance. We evaluated our prediction performance by choosing the model that led to the highest predictive accuracy over different time scales. However, in practical applications, it is crucial to determine the appropriate level of aggregation and moving window size in advance or to incorporate it into the cross-validation setup. Choosing the optimal time scale retrospectively can hinder real-time applications and potentially lead to overfitting. Thus, in future research methods should be applied to determine the optimal time-scale beforehand (Verachtert et al., 2022).

One limitation of our study is that a significant portion of the ESM measures and passive measures employed were not validated, which introduces potential challenges when interpreting our findings (Flake & Fried, 2020; Langener et al., 2023). It remains uncertain which specific aspects of social behavior are captured by the selected passive measures as results are inconsistent across studies and participants (Langener et al., 2023). For example, the association between the distance traveled and the number of interactions that a person had differed between people with schizophrenia and healthy controls (Fulford et al., 2021). Therefore, it is crucial for future research to prioritize the development and validation of measures that characterize social situations and the social environment. Additionally, we decided to classify apps based on how they were categorized in the Google Play Store. Fortunately, more scientific classification systems have recently become available, such as the one developed by Schoedel et al. (2022), which are recommended for use in the future.

In our study, we used positive and negative affect as an indicator of mood. This decision was based on previous research indicating a link between short-term emotions, commonly measured through positive and negative affect scales, and psychological well-being (Houben et al., 2015). However, a recent study suggests that researchers should be more critical when using a score that consists of multiple items and that using single items might be superior (Cloos et al., 2023; McNeish & Wolf, 2020). Thus, it would be interesting to investigate how results would change if we would only aim to predict the score of a single item, such as “I feel happy” or “I feel sad”, instead of positive or negative affect. In addition, the selection of items to measure positive and negative affect or other constructs in ESM research is often arbitrary. This can reduce construct validity (Bringmann et al., 2022; Flake & Fried, 2020), which could potentially lead to lower predictive performance when attempting to predict these constructs.

Our study employed a relatively small sample size of students (N = 11), with a majority of participants being female students (n = 10). It is important to acknowledge the limitations of this sample composition, as it may restrict the generalizability of our findings to a broader population. To gain a more comprehensive understanding of the predictive accuracy and its potential variations, future research would benefit from utilizing larger and more diverse samples. This goes in line with the current call to have larger samples for studies using passive smartphone measures in order to get more robust findings. One potential solution could be to pool data across studies, even though this might come with privacy concerns (e.g., Davidson, 2022; Huckvale et al., 2019). Furthermore, it is challenging to combine data from different studies as measures might differ, for example, the frequency of ESM assessments and collection of passive data. Additionally, constructs across different studies are likely to be operationalized in different ways which makes it further hard to pool data across studies (e.g., Davidson, 2022; Huckvale et al., 2019).

The variability in predictive ability and importance of variables in predicting mood across participants indicates that it is difficult to generalize our findings, even in this sample with participants from fairly homogeneous backgrounds. Our findings also suggest that, despite our intensive data collection, the sample sizes for the prediction models may have been a limiting factor Thus, future research using individual-level tailored models to predict mood within individuals based using similar methods and larger sample sizes are recommended.

Another limitation of our study comes from the mixed design incorporating both event-based and signal-based ESM for self-reporting social behavior. In the signal-based format, participants retrospectively reported all social interactions since the last questionnaire during daily semi-random assessments. During the event-based format, participants reported social interactions immediately after they occurred. Each format was implemented for a duration of two weeks. It is possible that participants’ reporting behavior may have varied after two weeks, potentially changing the relationship between ESM predictors and mood. However, we employed a rolling window cross-validation strategy, which involved retraining the model. Thus, the model is likely to capture changes in participants' response patterns. Hence, while there might have been a temporary decline in performance during the transition period from one reporting format to the other, it is unlikely to have significantly impacted the overall predictive accuracy.

We employed various self-reported questionnaires to measure social behavior and mood. One limitation of these measures is their burden on participants, which could potentially impact the validity of the collected data (e.g., Asselbergs et al., 2016; Eisele et al., 2020). In contrast, passive smartphone measures are a less burdensome alternative. For future research, it would be interesting to investigate how the respondents’ motivation to complete questionnaires influenced the validity of the data and subsequently affected the predictive performance compared to using only passive measures.

One strength of our study is that we conducted various robustness checks using different datasets, which enhances the reliability of our findings. However, it is important to note that we applied certain preprocessing steps without investigating their impact on the results. Specifically, data quality and missing data are important considerations when using digital phenotyping data (Bähr et al., 2022; Niemeijer et al., 2023; Roos et al., 2023). For example, different devices than those tested while developing the app may lead to missing data or third-party apps may interfere with the app used to passively collect data (Bähr et al., 2022). In this study, we labeled passive smartphone measures as missing if the corresponding sensor was not recorded for 24 h (choosing a different time window may produce different outcomes). Nevertheless, the data coverage in our sample was rather high, with minimal missing data due to potential problems with the app (data coverage per hour above 85%, see Supplementary Material section *Data Quality*). Therefore, we do not anticipate any significant changes in the results. Furthermore, when using a larger level of aggregation (i.e., 24 h), it could be argued that the first day of data should be excluded since participants were unable to log their social behavior prior to the study start (i.e., the first ESM measurement). However, we made the decision to utilize all available data to not lose any valuable information. As a result, it is possible that the first mood assessments include unrecorded social interactions that took place before the study started. Nevertheless, considering that most participants reported few interactions during the study, we believe that excluding the first day of data would not have significantly impacted the predictive performance.

## Conclusion

Our study highlights the challenges with accurately predicting mood based on social behavior and suggest that employing a combination of measures yields higher predictive accuracy. We demonstrated the substantial influence of the chosen time scale for aggregating predictors and analyzing data on predictive accuracy, which supports calls for future research to determine appropriate time scales for psychological constructs a priori. We encourage future research that integrates across multiple measures (i.e., ESM, egocentric networks, digital phenotyping) to improve our understanding of how social behavior impacts mood and well-being in everyday life.

### Supplementary Information

Below is the link to the electronic supplementary material.

Supplementary file1 (DOCX 13793 kb)

## Footnotes

1 We are using Shapley values due to their ability to handle categorical and noncategorical predictors without introducing significant biases (Henninger et al., 2023). Shapley values can be computed in both the training and testing sets, and a recent study found that both approaches produce similar results (Borup et al., 2022). A drawback of using Shapley values is that they only measure a variable's contribution relative to the mean prediction, without directly assessing the actual prediction performance. Recently, a new method has been developed, which calculates the Shapley value based on the actual prediction performance (Borup et al., 2022).

2 It is important to distinguish between robustness checks, such as using different datasets, and parameters that are expected to impact the model's performance, such as level of aggregation and window size. Ideally, the former should not result in different performances (and any improved performance based on a particular robustness check is not interpreted as it being a better model), but the latter may and should be considered as hyperparameters to be fine-tuned. In this section, we refer to the use of different datasets based on different preprocessing steps, which ideally should not change the performance of the models.
