# Supplementary material for: Predicting Mood Based on the Social Context Measured Through the Experience Sampling Method, Digital Phenotyping, and Social Networks
Source: Adm Policy Ment Health. 2024 Jan 10;51(4):455–75. doi: 10.1007/s10488-023-01328-0 (PMC11196304; doi:10.1007/s10488-023-01328-0)
Supplement: Supplementary file 1 — Supplementary file1 (DOCX 13793 kb) [file 10488_2023_1328_MOESM1_ESM.docx]

**Supplementary Material**

**Predicting mood based on the social environment measured through the experience sampling method, digital phenotyping, and social networks**

[Robustness Checks 2](#_Toc150194816)

[Prediction Lag 30 Minutes 4](#_Toc150194817)

[Manually Adjusting Only One Interaction 6](#_Toc150194818)

[Test Length Equal 8](#_Toc150194819)

[Descriptive 10](#_Toc150194820)

[Mean in the Training Set as Prediction 35](#_Toc150194821)

[Robustness Check Participant 5 37](#_Toc150194822)

[Data Quality 38](#_Toc150194823)

# Robustness Checks

To ensure the robustness of our results, we used four different datasets, which are summarized in Table 2 of the manuscript. The first dataset, referred to as *lag 60 minutes* in Table S.0, used predictors that were summarized 60 minutes prior to the mood assessment (see Figure 2 in the manuscript). This dataset was used for the analysis of the results reported thus far. The second dataset, referred to as *lag 30 minutes*, differed from the first by summarizing the predictors 30 minutes prior to the mood assessment. The third dataset, referred to as *lag 60 minutes (only one adjustment)*, differed from the first by adjusting the end time of only one interaction of one participant that would otherwise have lasted for zero minutes. The last dataset, referred to as *lag 60 minutes (equal test length)*, used an equal test set length to calculate the performance measures.

To test the robustness of our results, we examined whether the R^2^ and mean average percentage error were similar across the different datasets. Each dataset involved running 396 models (11 participants x 3 levels of aggregation x 3 moving window sizes x 4 sets of included predictors). We examined the correlation between the R^2^ values obtained from different datasets. Additionally, we explored the correlation between the mean average percentage error values obtained from the same datasets. For positive affect, we observed a strong association between the R^2^ values and the mean average percentage values across the different datasets (all r > .97). This suggests that when predicting positive affect, the results are robust across different datasets.

For negative affect, the association between R^2^ values across different datasets revealed a high correlation for three out of the four datasets. Specifically, when comparing the use of a lag of 60 minutes to a lag of 30 minutes or a dataset with only one adjustment, we observed a strong correlation (all r > .98). A similar pattern emerged when assessing the association of the mean average percentage error between different datasets. However, there was a notable difference when utilizing a dataset that included an equal test set length for different moving window sizes. In this case, the correlation between the different R^2^ values was moderate (r = .7). Upon further inspection, we found that this was due to one participant (participant 5, see figure S.51 for more information) who had large values of negative affect at the beginning of the study. By making the test length equal across different moving window sizes, we excluded those data points. This changed the variance of the data and therefore the R^2^. The mean average percentage error remained high with a correlation of r = .98.

We also checked whether the best predictors included in a model were robust by recalculating how often different sets of predictors (i.e., using all predictors, only egocentric network, only Behapp, or only ESM) resulted in an acceptable prediction. Table 1 shows how often this was the case based on the different datasets.

For positive affect, the results seem robust. Across all datasets, using all predictors performed the best, followed by using only Behapp and only ESM. Using only egocentric network variables performed worse.

For negative affect, the results seem again robust for three of the datasets. Using all predictors performed the best, followed by using only ESM and only egocentric network variables. Using only Behapp seemed to perform worse across those three datasets. The fourth dataset, which used an equal test length, differed. Here, using all predictors and only ESM performed equally well, followed by using only network data and only Behapp (which also performed equally well). The reason for this difference is likely the unstable results of participant 5.

**Table 1**

*Robustness of Results of Different Predictor Sets Included*

|  | Lag 60 Min | Lag 30 Min | Lag 60 Min (only one adjustment) | Lag 60 Min (equal test length) |
| --- | --- | --- | --- | --- |
| *Positive Affect* |  |  |  |  |
| All | 22.2% (1) | 23.2% (1) | 21.2% (1) | 22.2% (1) |
| Only Network | 10.1% (4) | 7.1% (4) | 10.1% (4) | 4% (4) |
| Only ESM | 13.1% (2) | 21.2% (2) | 13.1% (2) | 19.2% (2) |
| Only Behapp | 12.1% (3) | 12.1% (3) | 12.1% (3) | 12.1% (3) |
| *Negative Affect* |  |  |  |  |
| All | 14.1% (1) | 18.2% (1) | 13.1% (1) | 12.1% (1) |
| Only Network | 6.1% (3) | 12.1% (3) | 6.1% (3) | 5.1% (2) |
| Only ESM | 12.1% (2) | 16.2% (2) | 12.1% (2) | 12.1% (1) |
| Only Behapp | 3% (4) | 3% (4) | 3% (4) | 5.1% (2) |

## Prediction Lag 30 Minutes

**Table 2**

*Results for the Best-Performing Model to Predict Positive Affect (Lag 30 Minutes) Including the Standard Deviation and Minimum Value Using Different Window Sizes and Levels of Aggregation*

|  | Predictors | Coefficient of Determination | Correlation | Mean Absolute Error | Mean Absolute Percentage Error | Window | Aggregation |
| --- | --- | --- | --- | --- | --- | --- | --- |
| 1 | Only Network | 0.07 (min = NA, SD = NA) | .35 (min = .19, SD = .05) | 1.93 (max = 2.04, SD = 0.06) | 46.75 (max = 47.78, SD = 2.15) | 20 | 24 |
| 2 | All | 0.05 (min = -0.29, SD = 0.12) | .3 (min = .11, SD = .06) | 1.17 (max = 1.26, SD = 0.03) | 19.22 (max = 20.2, SD = 1.15) | 15 | 24 |
| 3 | Only Behapp | 0.09 (min = -0.12, SD = 0.07) | .41 (min = .2, SD = .09) | 1.52 (max = 1.83, SD = 0.11) | 42.6 (max = 53.45, SD = 5.01) | 15 | 3 |
| 4 | Only ESM | 0.07 (min = -0.17, SD = 0.08) | .32 (min = .13, SD = .07) | 1.45 (max = 1.59, SD = 0.04) | 24.45 (max = 26.93, SD = 0.82) | 20 | 3 |
| 5 | All | 0.21 (min = -0.02, SD = 0.07) | .5 (min = .34, SD = .06) | 0.63 (max = 0.68, SD = 0.02) | 8.43 (max = 8.92, SD = 0.33) | 15 | 24 |
| 6 | All | 0.1 (min = -0.21, SD = 0.1) | .36 (min = .08, SD = .1) | 1.32 (max = 1.53, SD = 0.07) | 18.61 (max = 22.55, SD = 1.3) | 30 | 6 |
| 7 | All | 0.02 (min = -0.3, SD = 0.1) | .28 (min = -.17, SD = .13) | 1.12 (max = 1.31, SD = 0.06) | 21.81 (max = 24.68, SD = 0.91) | 20 | 24 |
| 8 | All | 0.21 (min = -0.04, SD = 0.09) | .47 (min = .21, SD = .09) | 1.19 (max = 1.37, SD = 0.05) | 18.02 (max = 20.58, SD = 0.96) | 20 | 6 |
| 9 | All | 0.04 (min = -0.2, SD = 0.09) | .27 (min = -.13, SD = .15) | 1.39 (max = 1.55, SD = 0.07) | 21.82 (max = 23.9, SD = 1.23) | 30 | 24 |
| 10 | Only Behapp | -0.05 (min = -00.23, SD = 0.06) | .21 (min = -.02, SD = .07) | 0.95 (max = 1.02, SD = 0.03) | 15.61 (max = 16.72, SD = 0.41) | 20 | 6 |
| 11 | Only Network | 0.04 (min = -.054, SD = 0.22) | .35 (min = -.06, SD = .17) | 0.73 (max = 0.91, SD = 0.07) | 9 (max = 11.03, SD = 0.87) | 20 | 24 |

**Table 3**

*Results for the Best-Performing Model to Predict Negative Affect (Lag 30 Minutes) Including the Standard Deviation and Minimum Value Using Different Window Sizes and Levels of Aggregation*

|  | Predictors | Coefficient of Determination | Correlation | Mean Absolute Error | Mean Absolute Percentage Error | Window | Aggregation |
| --- | --- | --- | --- | --- | --- | --- | --- |
| 1 | Only Network | 0.05 (min = NA, SD = NA) | .2 (min = .05, SD = .05) | 1.65 (max = 1.75, SD = 0.07) | 49.61 (max = 52.24, SD = 2.61) | 30 | 6 |
| 2 | All | -0.01 (min = -0.3, SD = 0.1) | .25 (min = -.1, SD = .11) | 0.66 (max = 0.75, SD = 0.03) | 43.52 (max = 51.46, SD = 3.29) | 15 | 24 |
| 3 | Only Behapp | -0.07 (min = -0.27, SD = 0.08) | .24 (min = 0, SD = .08) | 1.86 (max = 2.04, SD = 0.07) | 39.56 (max = 45.43, SD = 2.88) | 20 | 24 |
| 4 | Only Behapp | 0.16 (min = -0.12, SD = 0.08) | .43 (min = .26, SD = .06) | 0.8 (max = 0.93, SD = 0.04) | 38.69 (max = 43.91, SD = 2.11) | 30 | 6 |
| 5 | All | 0.14 (min = -1.2, SD = 0.56) | .45 (min = -.12, SD = .21) | 0.77 (max = 0.88, SD = 0.06) | 28.53 (max = 32.12, SD = 1.48) | 20 | 24 |
| 6 | All | 0.22 (min = -0.02, SD = 0.07) | .47 (min = .29, SD = .06) | 0.81 (max = 0.9, SD = 0.04) | 31.62 (max = 34.7, SD = 1.37) | 30 | 6 |
| 7 | Only Network | 0.04 (min = -0.33, SD = 0.11) | .34 (min = .08, SD = .08) | 0.96 (max = 1.19, SD = 0.07) | 18.38 (max = 23.98, SD = 1.89) | 20 | 24 |
| 8 | Only ESM | 0.07 (min = -0.11, SD = 0.06) | .34 (min = .2, SD = .06) | 0.8 (max = 0.88, SD = 0.04) | 49.63 (max = 55.51, SD = 1.79) | 30 | 24 |
| 9 | All | 0.05 (min = -0.21, SD = 0.08) | .24 (min = .06, SD = .06) | 1.35 (max = 1.53, SD = 0.06) | 43.17 (max = 52.09, SD = 3.47) | 30 | 24 |
| 10 | Only Behapp | 0.01 (min = -0.21, SD = 0.07) | .24 (min = .05, SD = .07) | 0.86 (max = 0.95, SD = 0.03) | 18.16 (max = 19.93, SD = 0.63) | 20 | 6 |
| 11 | Only ESM | 0.26 (min = -0.18, SD = 0.16) | .59 (min = .19, SD = .13) | 0.55 (max = 0.72, SD = 0.06) | 36.11 (max = 45.27, SD = 4.24) | 30 | 24 |

## Manually Adjusting Only One Interaction

**Table 4**

*Results for the Best-Performing Model to Predict Positive Affect (Lag 60 Minutes, with Manual Adjustment of a Single Interaction) Including Standard Deviation and Minimum Values Across Various Window Sizes and Levels of Aggregation*

|  | Predictors | Coefficient of Determination | Correlation | Mean Absolute Error | Mean Absolute Percentage Error | Window | Aggregation |
| --- | --- | --- | --- | --- | --- | --- | --- |
| 1 | All | 0.04 (min = -0.15, SD = 0.06) | .3 (min = .13, SD = .05) | 1.96 (max = 2.07, SD = 0.04) | 46.03 (max = 49.53, SD = 1.53) | 15 | 3 |
| 2 | All | 0.03 (min = -0.28, SD = 0.09) | .28 (min = .08, SD = .06) | 1.19 (max = 1.32, SD = 0.06) | 19.56 (max = 20.71, SD = 1.4) | 15 | 24 |
| 3 | Only Behapp | 0.09 (min = -0.12, SD = 0.07) | .41 (min = .2, SD = .09) | 1.52 (max = 1.83, SD = 0.11) | 42.6 (max = 53.45, SD = 5.01) | 15 | 3 |
| 4 | All | 0.06 (min = -0.1, SD = 0.05) | .3 (min = .13, SD = .05) | 1.43 (max = 1.56, SD = 0.04) | 24.51 (max = 26.1, SD = 0.54) | 20 | 24 |
| 5 | Only Network | 0.21 (min = -0.17, SD = 0.13) | .52 (min = .33, SD = .07) | 0.66 (max = 0.7, SD = 0.03) | 8.69 (max = 9.19, SD = 0.48) | 15 | 24 |
| 6 | All | 0.07 (min = -0.24, SD = 0.11) | .33 (min = .05, SD = .1) | 1.34 (max = 1.56, SD = 0.08) | 18.75 (max = 22.88, SD = 1.4) | 30 | 6 |
| 7 | Only Network | -0.02 (min = -0.43, SD = 0.17) | .26 (min = -.13, SD = .15) | 1.16 (max = 1.34, SD = 0.07) | 21.98 (max = 25.22, SD = 1.29) | 30 | 24 |
| 8 | All | 0.19 (min = -0.03, SD = 0.08) | .45 (min = .22, SD = .09) | 1.21 (max = 1.37, SD = 0.05) | 18.24 (max = 20.53, SD = 0.88) | 20 | 6 |
| 9 | Only Behapp | 0.03 (min = -0.26, SD = 0.12) | .3 (min = -.14, SD = .19) | 1.4 (max = 1.56, SD = 0.08) | 22.03 (max = 23.85, SD = 1.17) | 30 | 24 |
| 10 | Only Behapp | -0.05 (min = -0.23, SD = 0.06) | .21 (min = -.02, SD = .07) | 0.95 (max = 1.02, SD = 0.03) | 15.61 (max = 16.72, SD = 0.41) | 20 | 6 |
| 11 | Only Network | 0 (min = -0.58, SD = 0.21) | .33 (min = -.13, SD = .16) | 0.73 (max = 0.93, SD = 0.07) | 8.97 (max = 11.3, SD = 0.83) | 20 | 24 |

**Table 5**

*Results for the Best-Performing Model to Predict Negative Affect (Lag 60 Minutes, with Manual Adjustment of a Single Interaction) Including Standard Deviation and Minimum Value Across Different Window Sizes and Levels of Aggregation*

|  | Predictors | Coefficient of Determination | Correlation | Mean Absolute Error | Mean Absolute Percentage Error | Window | Aggregation |
| --- | --- | --- | --- | --- | --- | --- | --- |
| 1 | Only Network | -0.08 (min = -0.34, SD = 0.09) | .18 (min = -.01, SD = .06) | 1.61 (max = 1.84, SD = 0.09) | 47.6 (max = 55.78, SD = 3.26) | 20 | 6 |
| 2 | All | -0.02 (min = -0.31, SD = 0.09) | .23 (min = -.1, SD = .1) | 0.67 (max = 0.76, SD = 0.02) | 45.31 (max = 52.16, SD = 2.39) | 15 | 24 |
| 3 | Only Behapp | -0.07 (min = -0.27, SD = 0.08) | .24 (min = 0, SD = .08) | 1.86 (max = 2.04, SD = 0.07) | 39.56 (max = 45.43, SD = 2.88) | 20 | 24 |
| 4 | Only Behapp | 0.16 (min = -0.12, SD = 0.08) | .43 (min = .26, SD = .06) | 0.8 (max = 0.93, SD = 0.04) | 38.69 (max = 43.91, SD = 2.11) | 30 | 6 |
| 5 | Only Network | 0.21 (min = -1.74, SD = 0.71) | .53 (min = -.07, SD = .2) | 0.71 (max = 0.89, SD = 0.08) | 25.43 (max = 30.12, SD = 1.5) | 20 | 24 |
| 6 | All | 0.21 (min = -0.04, SD = 0.07) | .47 (min = .27, SD = .06) | 0.81 (max = 0.92, SD = 0.04) | 32.13 (max = 35.34, SD = 1.45) | 30 | 6 |
| 7 | All | -0.01 (min = -0.21, SD = 0.06) | .26 (min = .04, SD = .07) | 0.99 (max = 1.19, SD = 0.06) | 18.56 (max = 23.61, SD = 1.73) | 20 | 24 |
| 8 | Only ESM | 0.02 (min = -0.11, SD = 0.05) | .31 (min = .22, SD = .04) | 0.85 (max = 0.89, SD = 0.04) | 55.54 (max = 57.42, SD = 1.94) | 30 | 24 |
| 9 | All | -0.03 (min = -0.22, SD = 0.05) | .13 (min = -.01, SD = .06) | 1.4 (max = 1.53, SD = 0.06) | 44.2 (max = 51.35, SD = 3.4) | 30 | 24 |
| 10 | Only Behapp | 0.01 (min = -0.21, SD = 0.07) | .24 (min = .05, SD = .07) | 0.86 (max = 0.95, SD = 0.03) | 18.16 (max = 19.93, SD = 0.63) | 20 | 6 |
| 11 | Only ESM | 0.3 (min = -0.22, SD = 0.17) | .59 (min = .17, SD = .13) | 0.54 (max = 0.73, SD = 0.07) | 35.12 (max = 47.09, SD = 5.22) | 30 | 24 |

## Test Length Equal

**Table 6**

*Results for the Best-Performing Model to Predict Positive Affect (Lag 60 Minutes, Equal Test Length) Including Standard Deviation and Minimum Value Across Different Window Sizes and Levels of Aggregation*

|  | Predictors | Coefficient of Determination | Correlation | Mean Absolute Error | Mean Absolute Percentage Error | Window | Aggregation |
| --- | --- | --- | --- | --- | --- | --- | --- |
| 1 | All | 0.01 (min = -0.14, SD = 0.04) | .24 (min = .13, SD = .03) | 1.88 (max = 2.02, SD = 0.04) | 44.84 (max = 45.92, SD = 0.77) | 20 | 6 |
| 2 | All | -0.03 (min = -0.26, SD = 0.08) | .27 (min = .08, SD = .06) | 1.13 (max = 1.32, SD = 0.06) | 17.46 (max = 19.53, SD = 0.67) | 15 | 24 |
| 3 | Only Behapp | 0.1 (min = -0.14, SD = 0.08) | .43 (min = .2, SD = .09) | 1.61 (max = 1.85, SD = 0.08) | 47.98 (max = 54.03, SD = 3.01) | 15 | 3 |
| 4 | Only ESM | 0.07 (min = -0.19, SD = 0.1) | .35 (min = .13, SD = .08) | 1.45 (max = 1.6, SD = 0.06) | 24.17 (max = 27.21, SD = 1.04) | 20 | 24 |
| 5 | Only ESM | 0.24 (min = -0.1, SD = 0.11) | .55 (min = .35, SD = .07) | 0.56 (max = 0.65, SD = 0.03) | 7.14 (max = 8.31, SD = 0.38) | 15 | 24 |
| 6 | All | 0.07 (min = -0.24, SD = 0.11) | .33 (min = .09, SD = .09) | 1.34 (max = 1.56, SD = 0.07) | 18.75 (max = 22.31, SD = 1.21) | 30 | 6 |
| 7 | All | 0.02 (min = -0.32, SD = 0.1) | .28 (min = -.18, SD = .14) | 1.15 (max = 1.34, SD = 0.06) | 22.12 (max = 25.27, SD = 0.95) | 20 | 24 |
| 8 | Only ESM | 0.21 (min = -0.16, SD = 0.11) | .47 (min = .18, SD = .09) | 1.21 (max = 1.46, SD = 0.08) | 18.36 (max = 21.87, SD = 1.15) | 20 | 6 |
| 9 | Only Behapp | 0.03 (min = -0.26, SD = 0.11) | .3 (min = -.14, SD = .19) | 1.4 (max = 1.61, SD = 0.08) | 22.03 (max = 24.99, SD = 1.02) | 30 | 24 |
| 10 | Only Behapp | -0.07 (min = -0.26, SD = 0.06) | .14 (min = -.03, SD = .08) | 0.92 (max = 1.02, SD = 0.03) | 15.81 (max = 17.01, SD = 0.41) | 30 | 3 |
| 11 | Only Network | 0.01 (min = -0.66, SD = 0.24) | .31 (min = -.17, SD = .18) | 0.73 (max = 0.96, SD = 0.08) | 9.13 (max = 11.79, SD = 0.97) | 30 | 24 |

**Table 7**

*Results for the Best-Performing Model to Predict Negative Affect (Lag 60 Minutes, Equal Test Length) Including Standard Deviation and Minimum Value Across Different Window Sizes and Levels of Aggregation*

|  | Predictors | Coefficient of Determination | Correlation | Mean Absolute Error | Mean Absolute Percentage Error | Window | Aggregation |
| --- | --- | --- | --- | --- | --- | --- | --- |
| 1 | Only Network | 0 (min = -0.33, SD = 0.11) | .23 (min = .01, SD = .07) | 1.53 (max = 1.82, SD = 0.1) | 48.59 (max = 55.18, SD = 2.5) | 20 | 6 |
| 2 | All | -0.1 (min = -0.29, SD = 0.06) | .07 (min = -.12, SD = .07) | 0.67 (max = 0.75, SD = 0.03) | 44.88 (max = 49.34, SD = 2.93) | 20 | 24 |
| 3 | Only Behapp | -0.06 (min = -0.24, SD = 0.06) | .25 (min = .03, SD = .06) | 1.89 (max = 2, SD = 0.06) | 41.85 (max = 47.57, SD = 3.36) | 20 | 24 |
| 4 | Only ESM | 0.21 (min = -0.06, SD = 0.1) | .49 (min = .27, SD = .08) | 0.8 (max = 0.9, SD = 0.04) | 37.1 (max = 44.13, SD = 2.53) | 20 | 3 |
| 5 | Only Network | -0.73 (min = -1.74, SD = 0.35) | .2 (min = -.07, SD = .11) | 0.59 (max = 0.7, SD = 0.04) | 24.48 (max = 30.12, SD = 1.98) | 20 | 3 |
| 6 | All | 0.21 (min = 0, SD = 0.07) | .47 (min = .31, SD = .05) | 0.81 (max = 0.92, SD = 0.04) | 32.13 (max = 35.33, SD = 1.39) | 30 | 6 |
| 7 | All | 0.01 (min = -0.23, SD = 0.08) | .23 (min = -.02, SD = .08) | 0.98 (max = 1.11, SD = 0.04) | 18.56 (max = 20.94, SD = 0.81) | 20 | 24 |
| 8 | Only ESM | 0.02 (min = -0.14, SD = 0.05) | .31 (min = .22, SD = .03) | 0.85 (max = 0.93, SD = 0.03) | 55.54 (max = 64.24, SD = 3.04) | 30 | 24 |
| 9 | Only Behapp | 0.02 (min = -0.3, SD = 0.1) | .3 (min = -.03, SD = .12) | 1.29 (max = 1.59, SD = 0.1) | 42.11 (max = 54.98, SD = 4.51) | 15 | 24 |
| 10 | Only Behapp | -0.01 (min = -0.16, SD = 0.05) | .21 (min = .07, SD = .06) | 0.88 (max = 0.94, SD = 0.03) | 18.35 (max = 19.79, SD = 0.58) | 20 | 6 |
| 11 | only ESM | 0.3 (min = -0.22, SD = 0.18) | .59 (min = .18, SD = .14) | 0.54 (max = 0.74, SD = 0.07) | 35.52 (max = 48.77, SD = 5.56) | 30 | 24 |

# Descriptive

**Figure 1**

*Total Minutes Spent in Face-to-Face Interactions (in Percentage)*


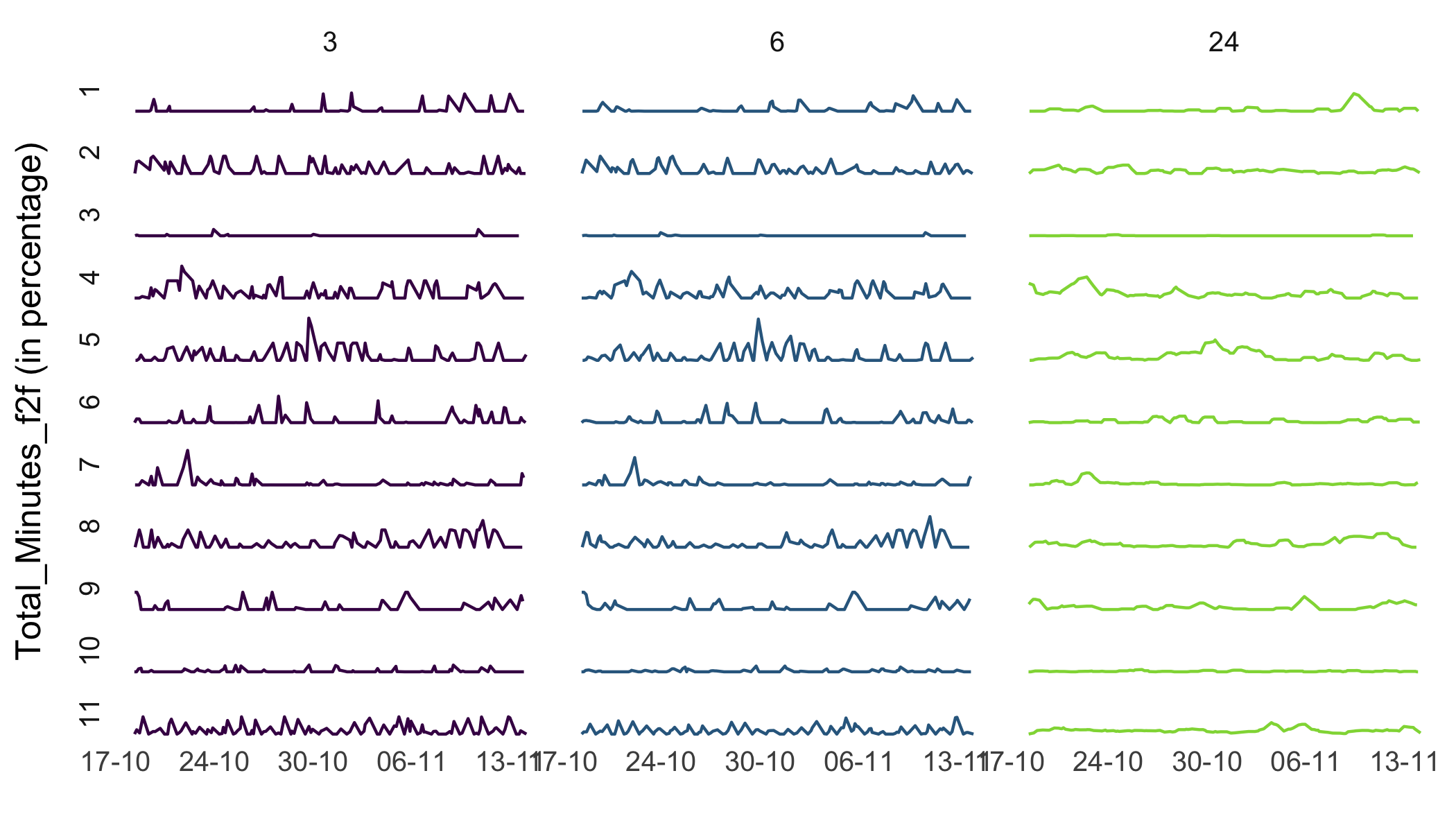


**Figure 2**

*Total Minutes Spent Calling (in Percentage)*
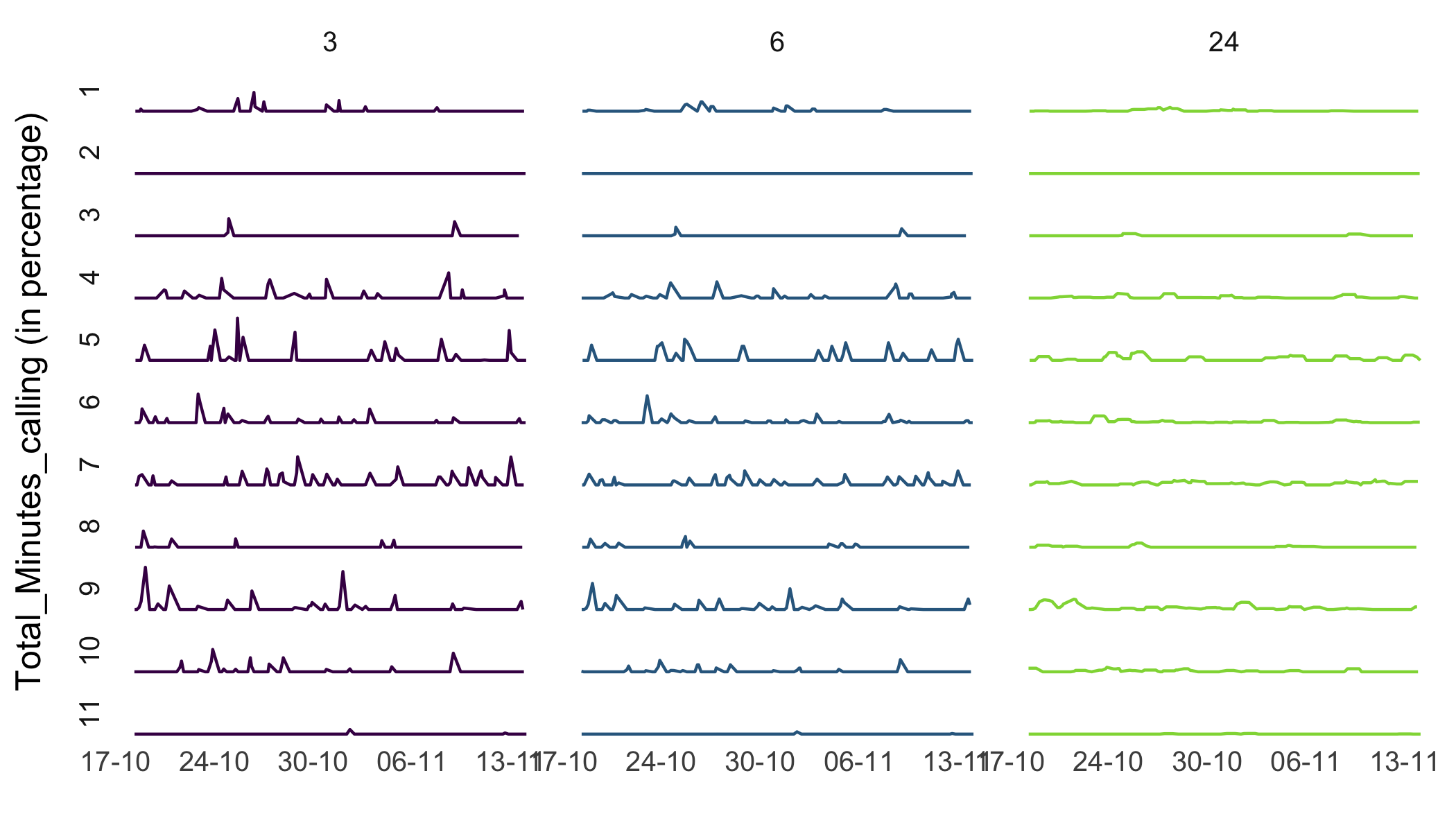


**Figure 3**

*Total Minutes Spent Texting (in Percentage)*


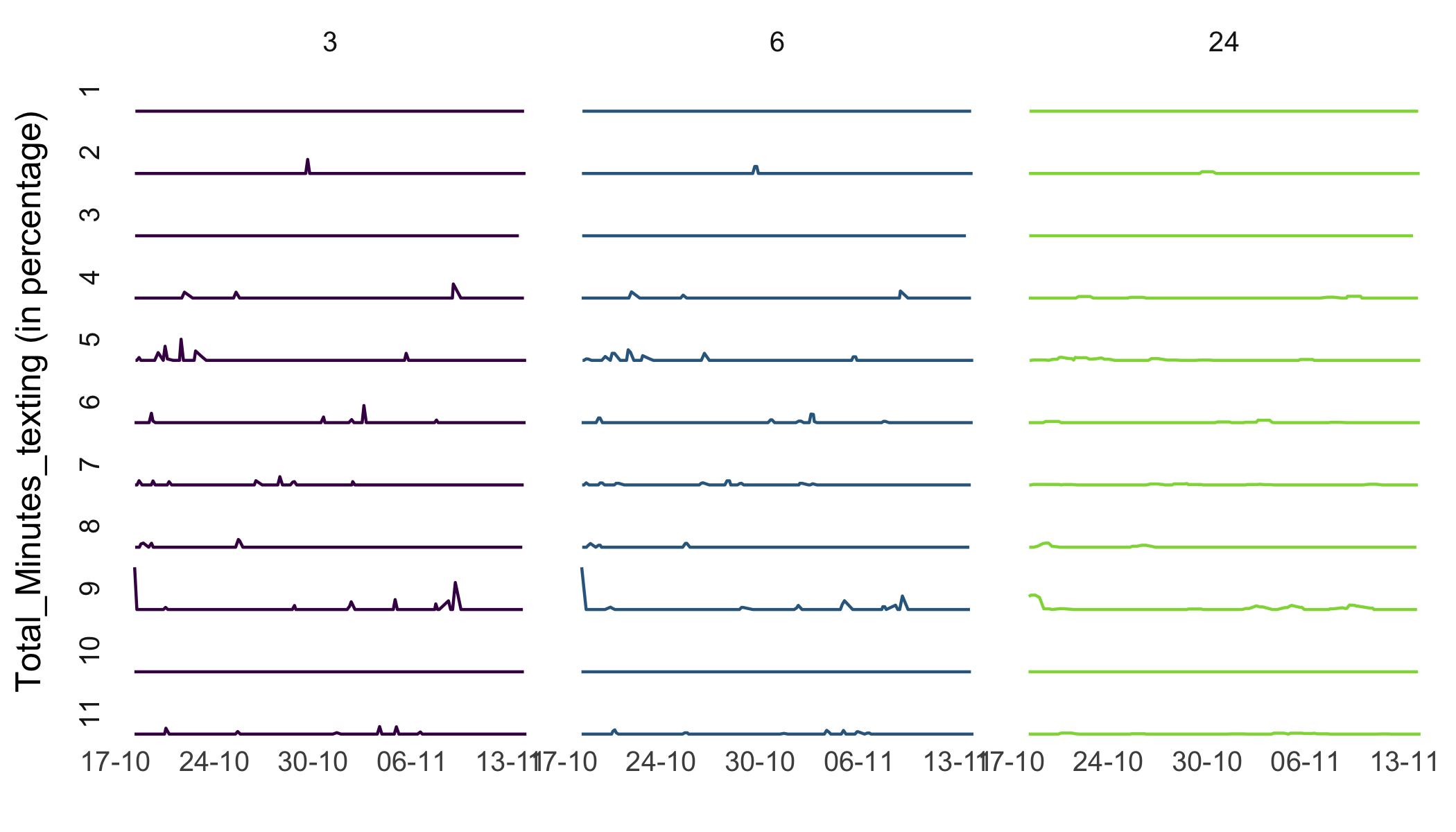


**Figure 4**

*Total Minutes Spent in Conversations With the Content “Striving Behavior” (i.e., Expressing Love or Affection, Joking Around, Meaningful, Catching Up; f2f, Call,Text, in Percentage)*
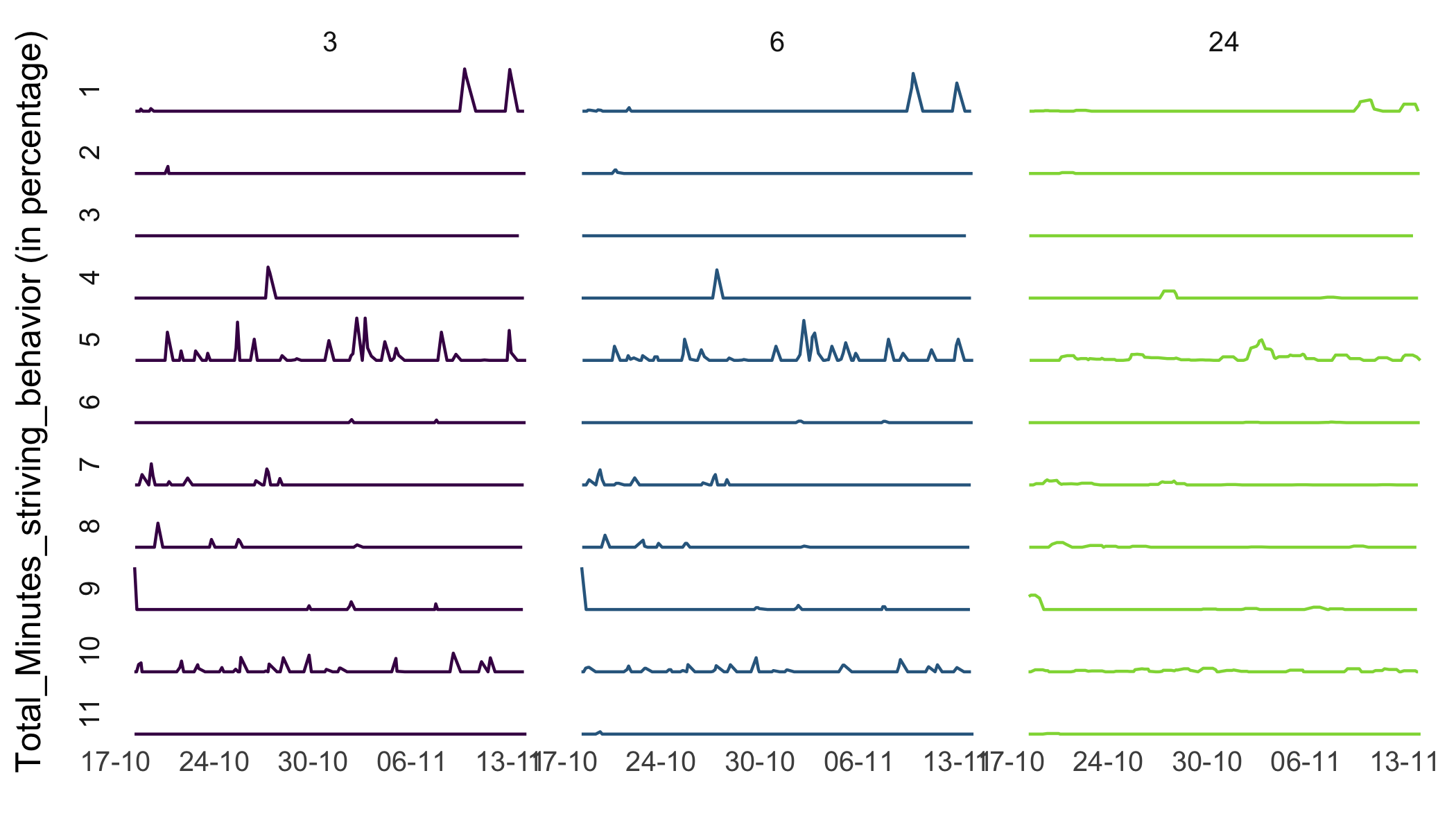


**Figure 5**

*Total Minutes Spent in Conversations With the Content “Mundane Maintenance Behavior” (i.e., Gossip, Task Talk, Small Talk, Making Plans, f2f, Call, Text, in Percentage)*
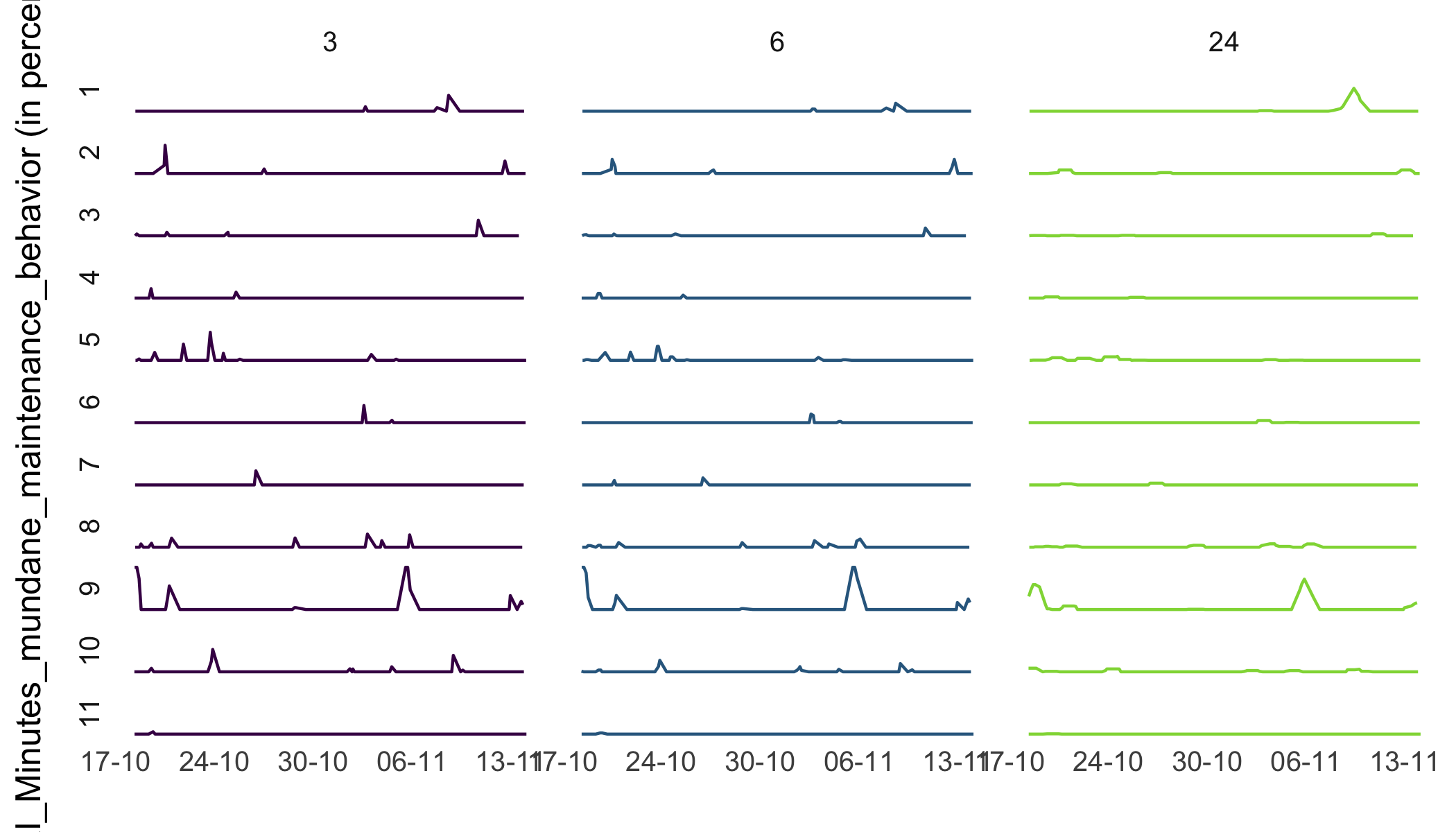


**Figure 6**

*Total Minutes Spent in Conversations With the Content “Work or School Talk” (in Percentage)*
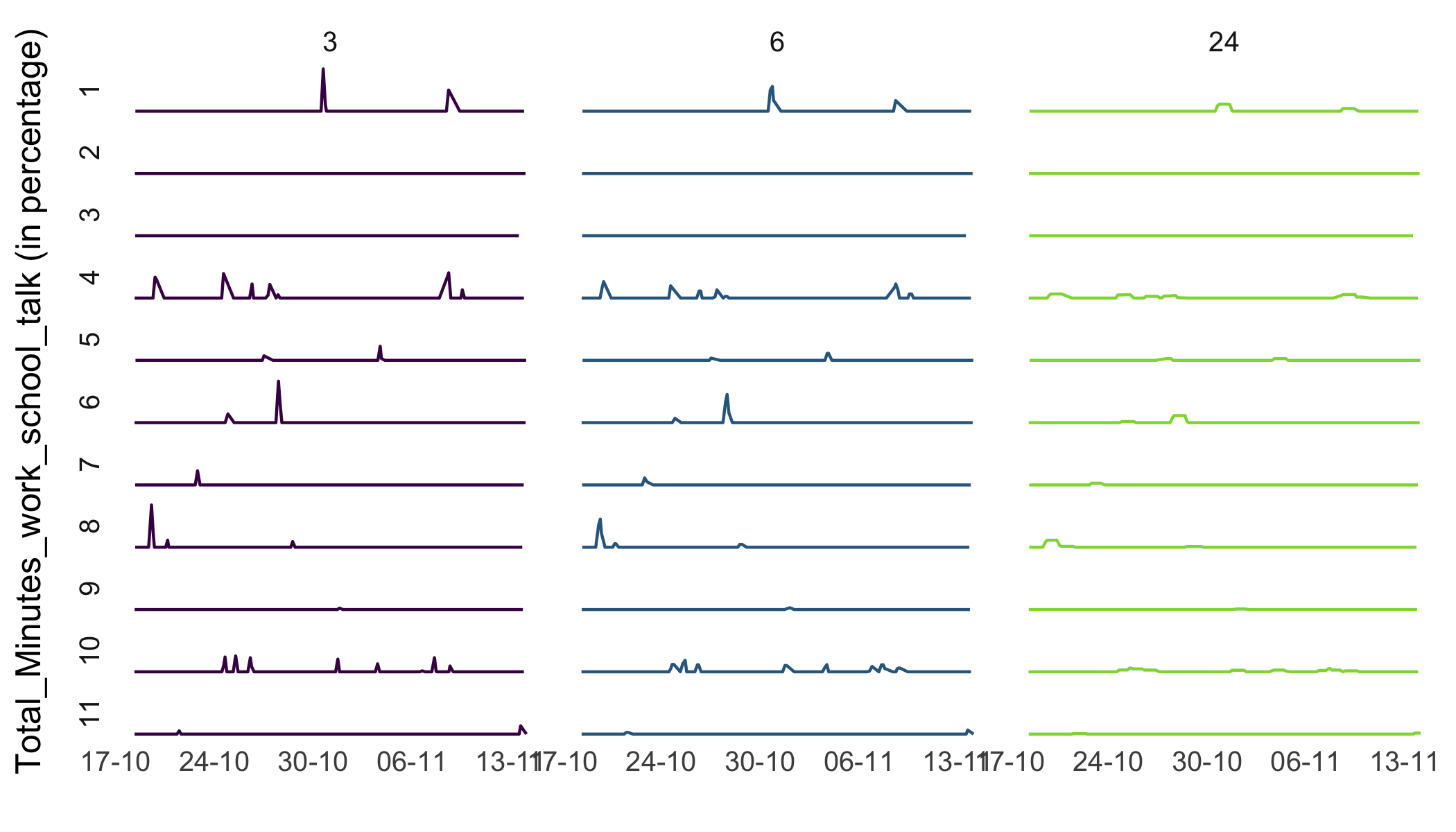


**Figure 7**

*Total Minutes Spent in Conversations With “negative content” (i.e., Complaining or Venting, Conflict or Disagreement; f2f, Call, Text, in Percentage)*
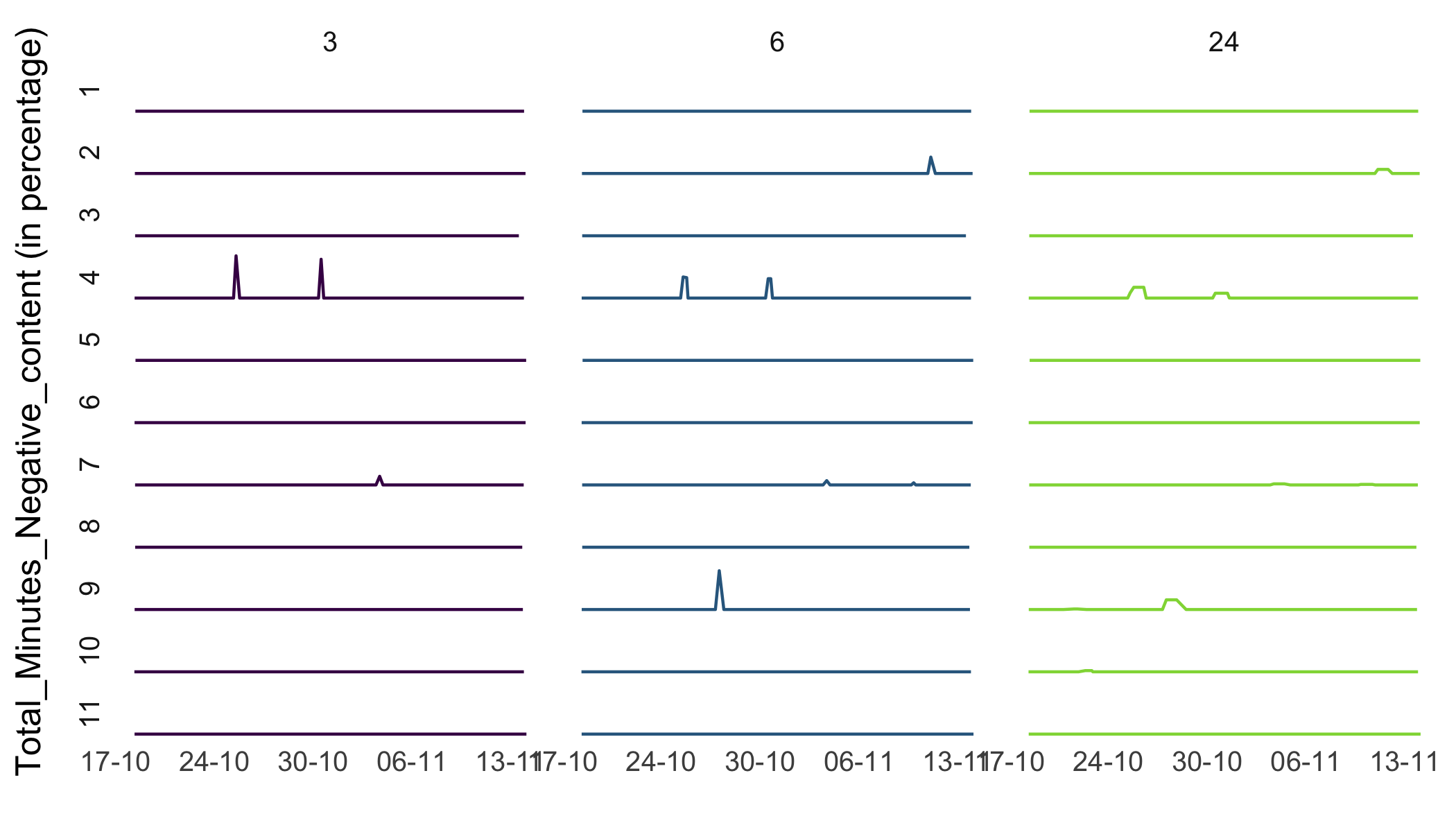


**Figure 8**

*Total Minutes Spent Interacting With Partner (in Percentage)*
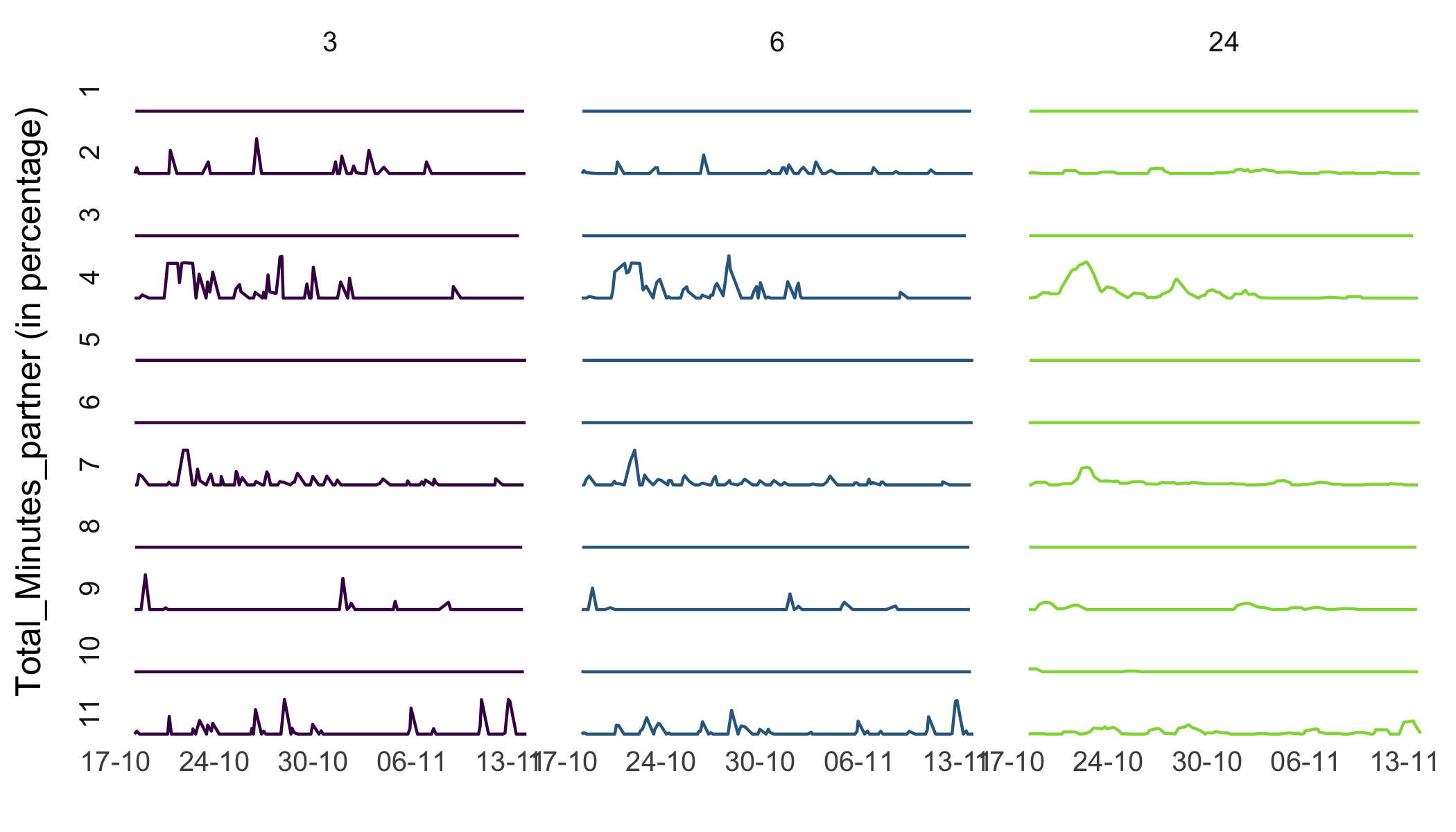


**Figure 9**

*Total Minutes Spent Interacting With Friend (in Percentage)*


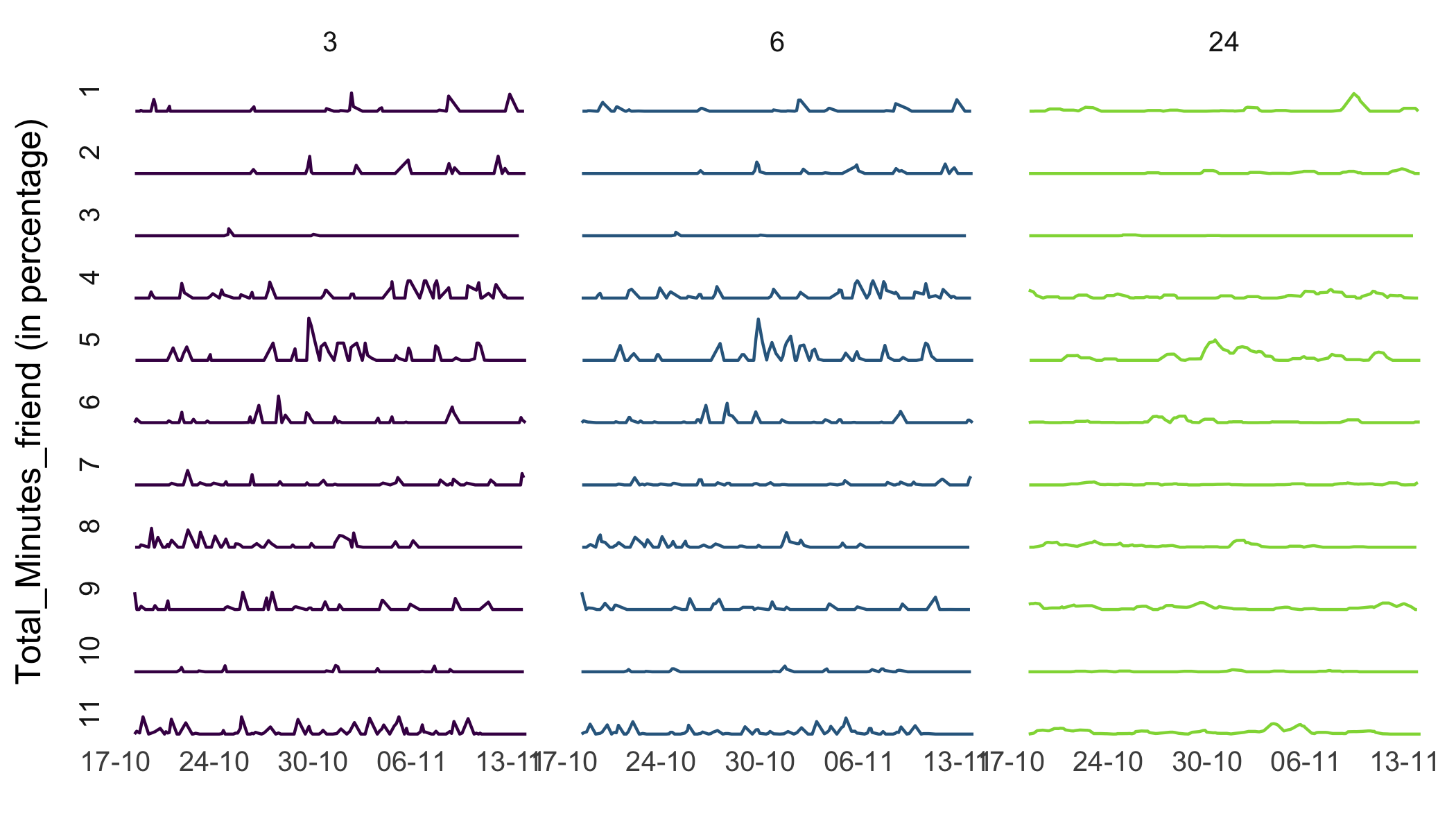


**Figure 10**

*Total Minutes Spent Interacting With Family (in Percentage)*
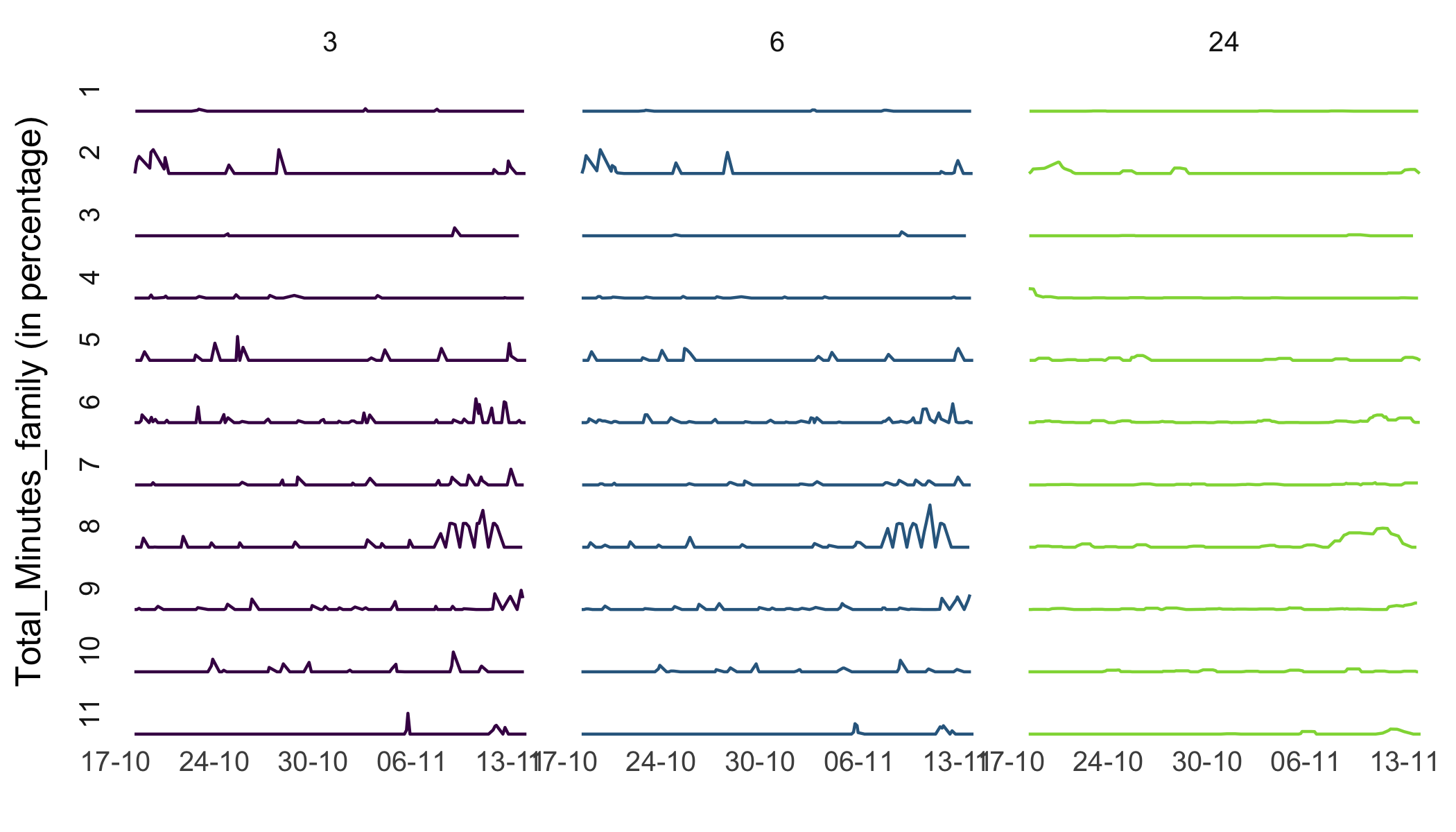


**Figure 11**

*Total Minutes Spent Interacting With Fellow Students or Colleagues (in Percentage)*
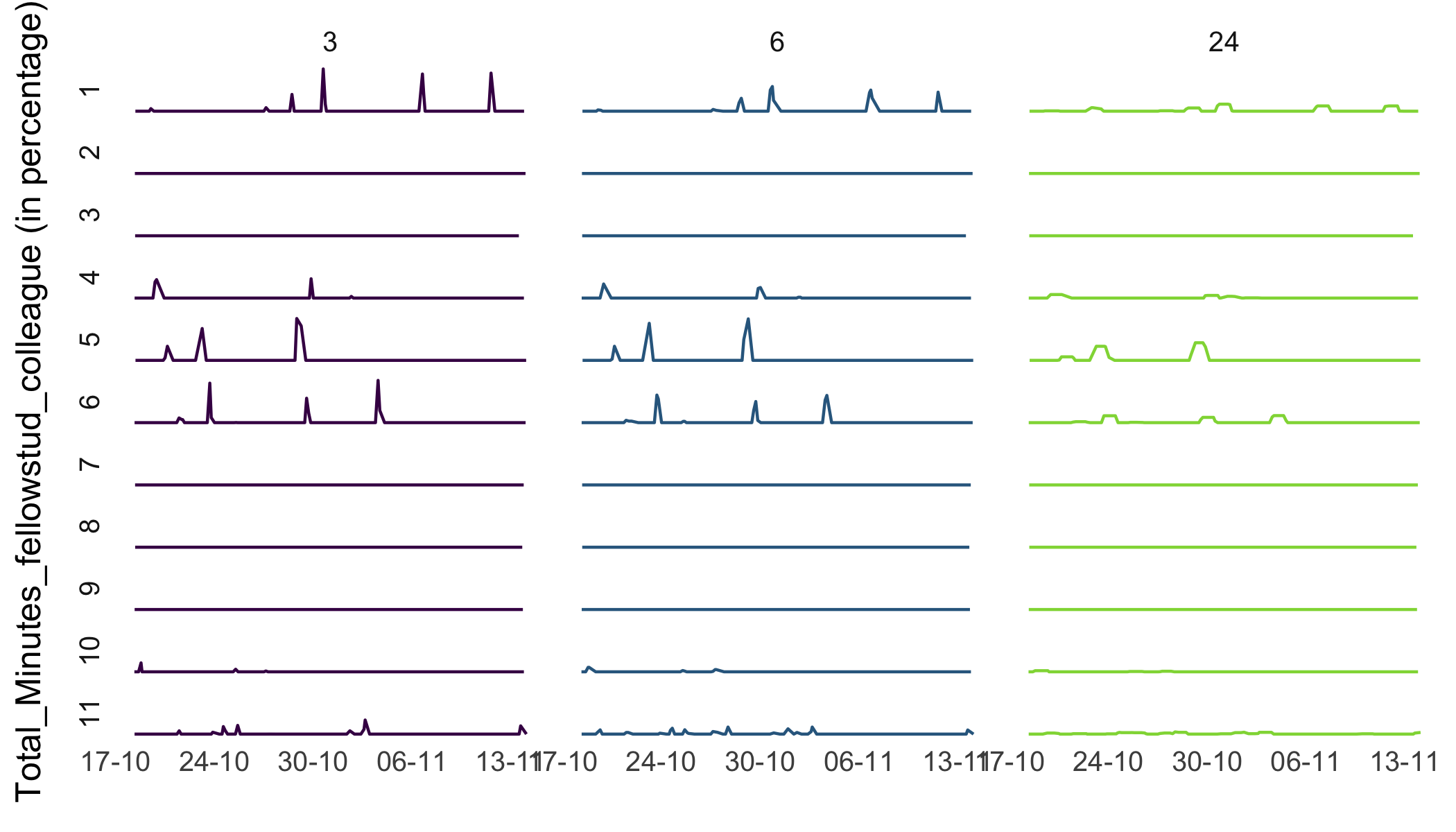


**Figure 12**

*Total Minutes Spent Interacting With Flatmates (in Percentage)*
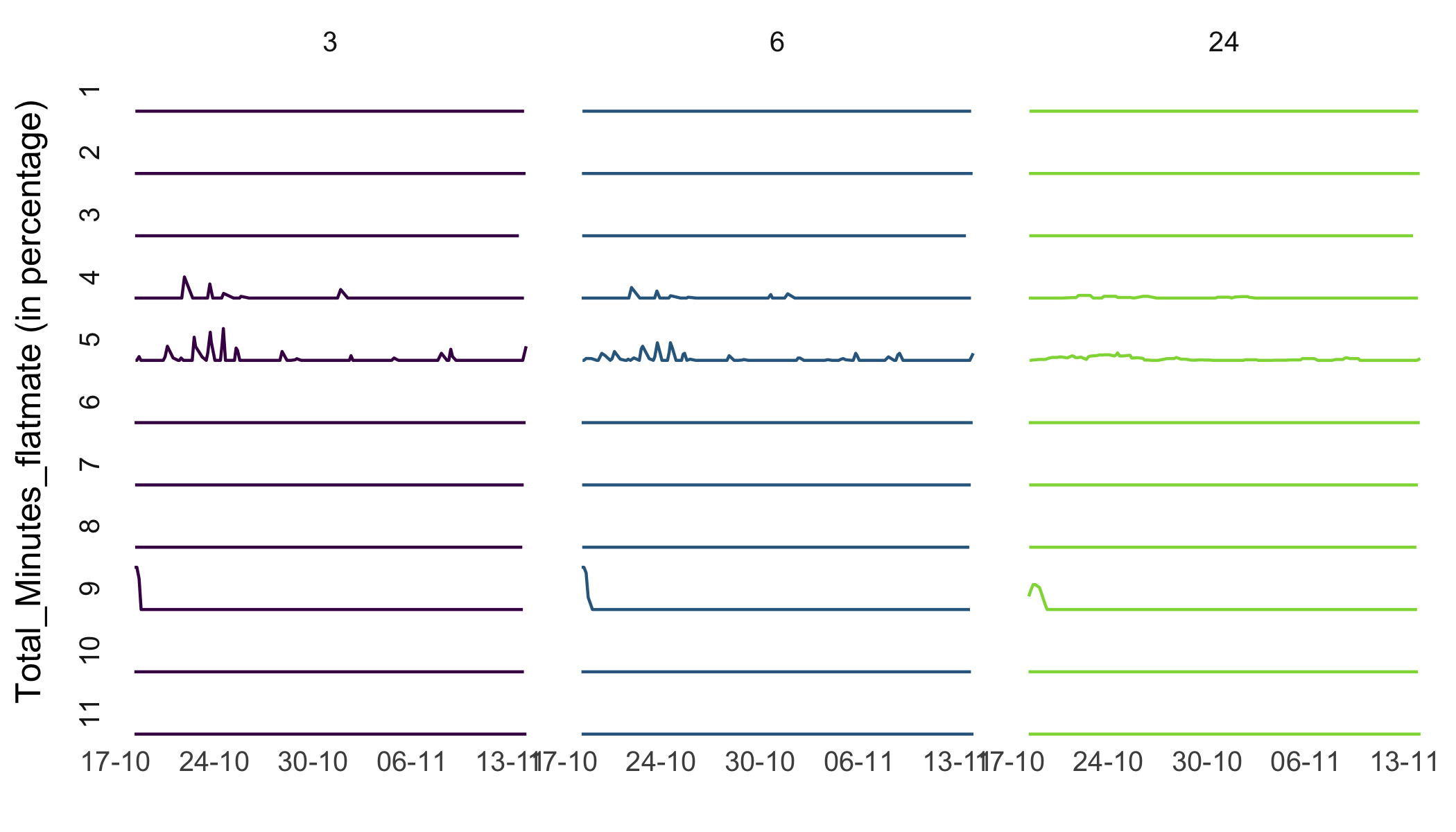


**Figure 13**

*Total Minutes Spent Interacting With Teacher or Superior (in Percentage)*
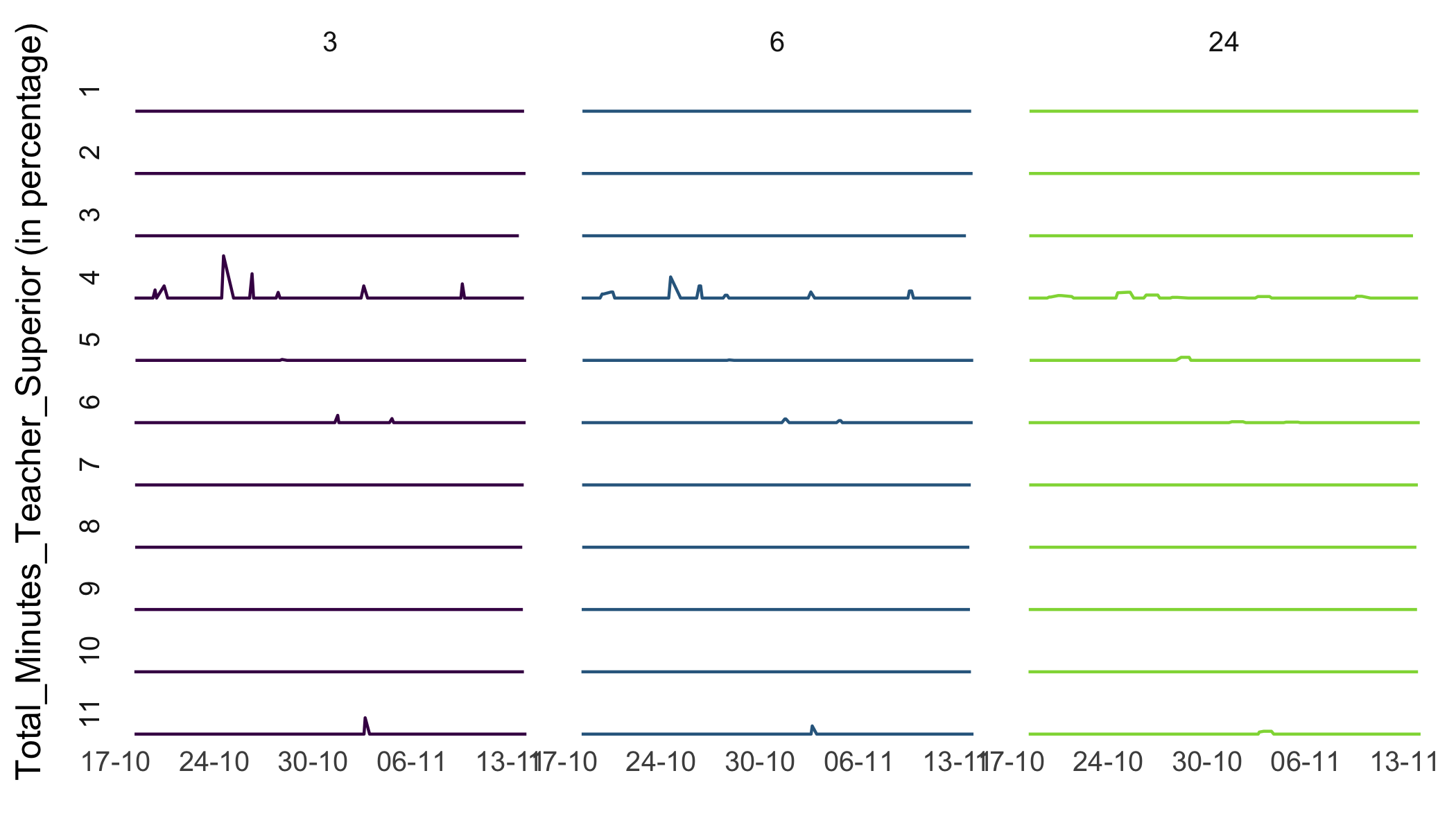


**Figure 14**

*Total Minutes Spent Interacting With Close Interaction Partner (in Percentage)*
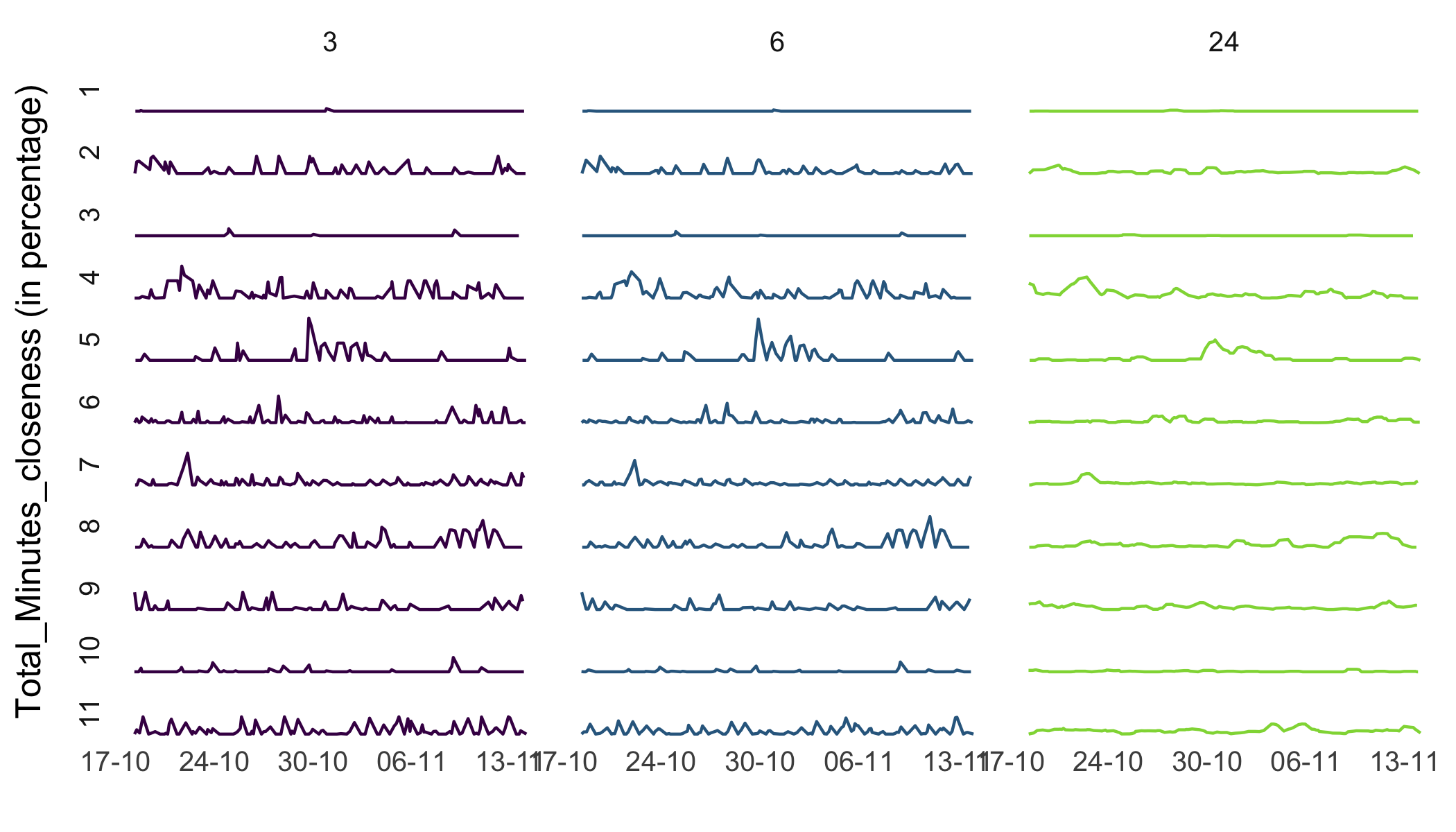


**Figure 15**

*Total Minutes Spent Interacting With Interaction Partner that Someone Discusses Personal Problems With (in Percentage)*
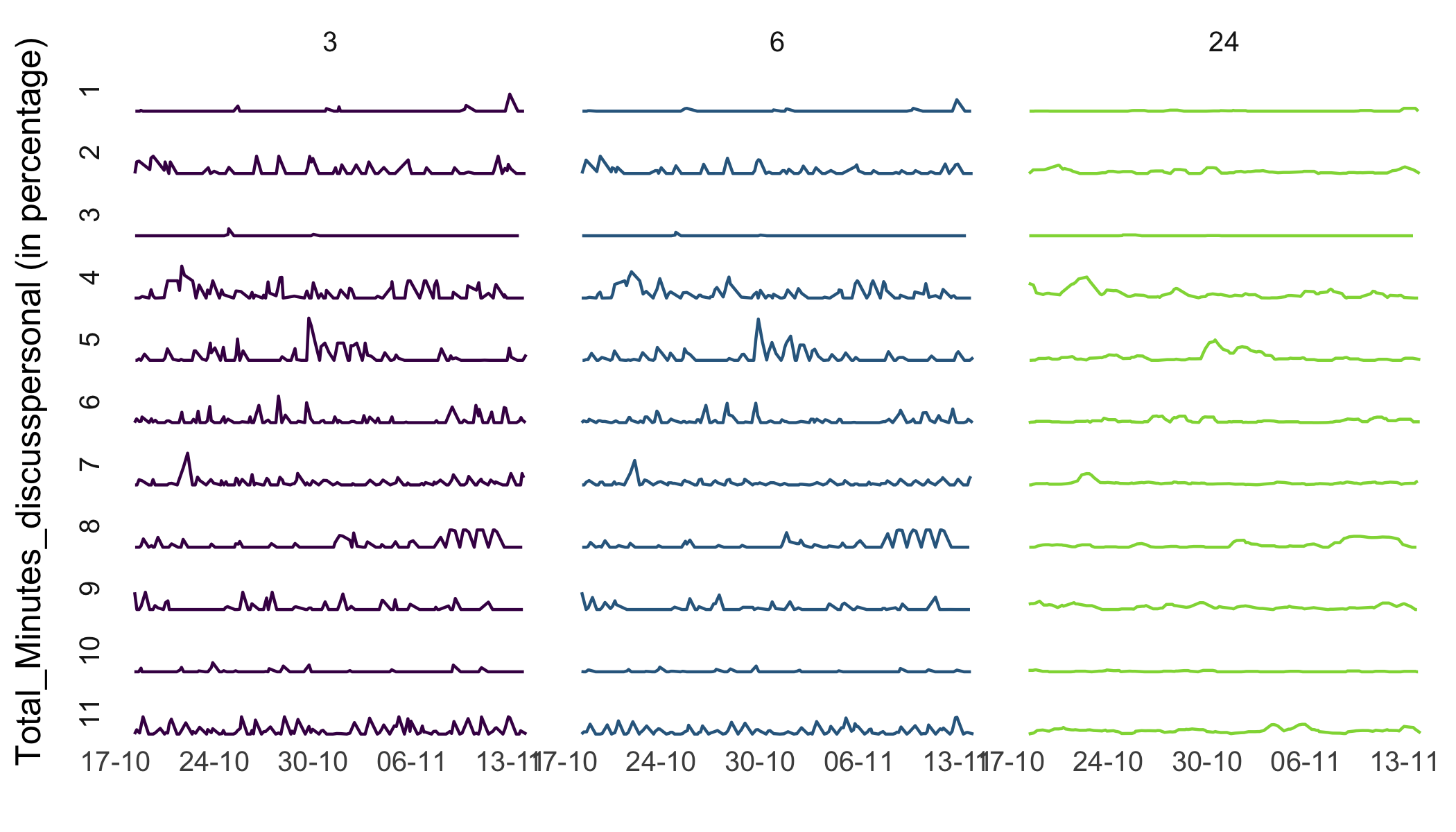


**Figure 16**

*Total Minutes Spent Interacting With Interaction Partner that Provides Emotional Support (in Percentage)*
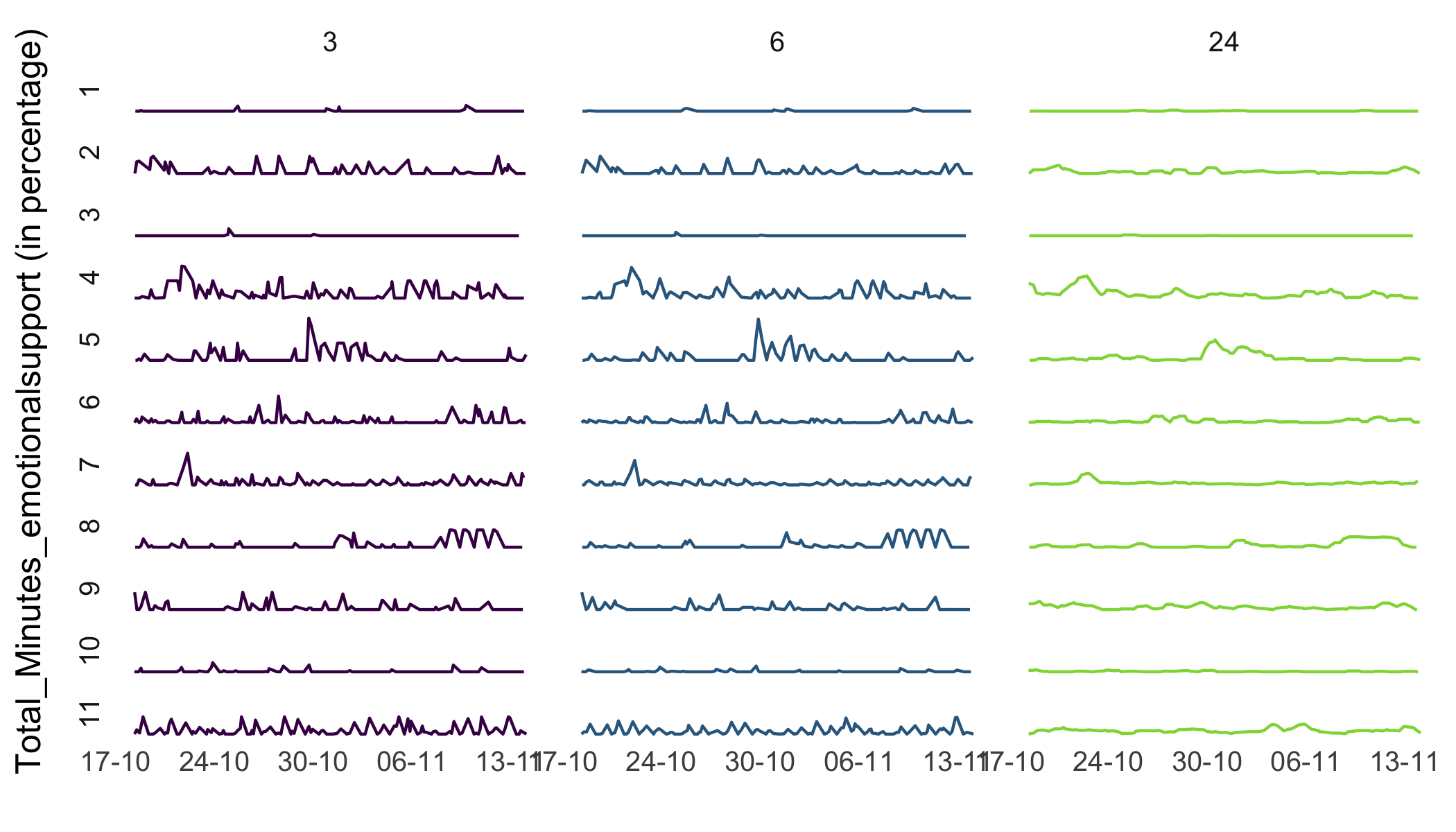


**Figure 17**

*Total Minutes Spent Interacting With Interaction Partner that Provides Practical Support (in Percentage)*
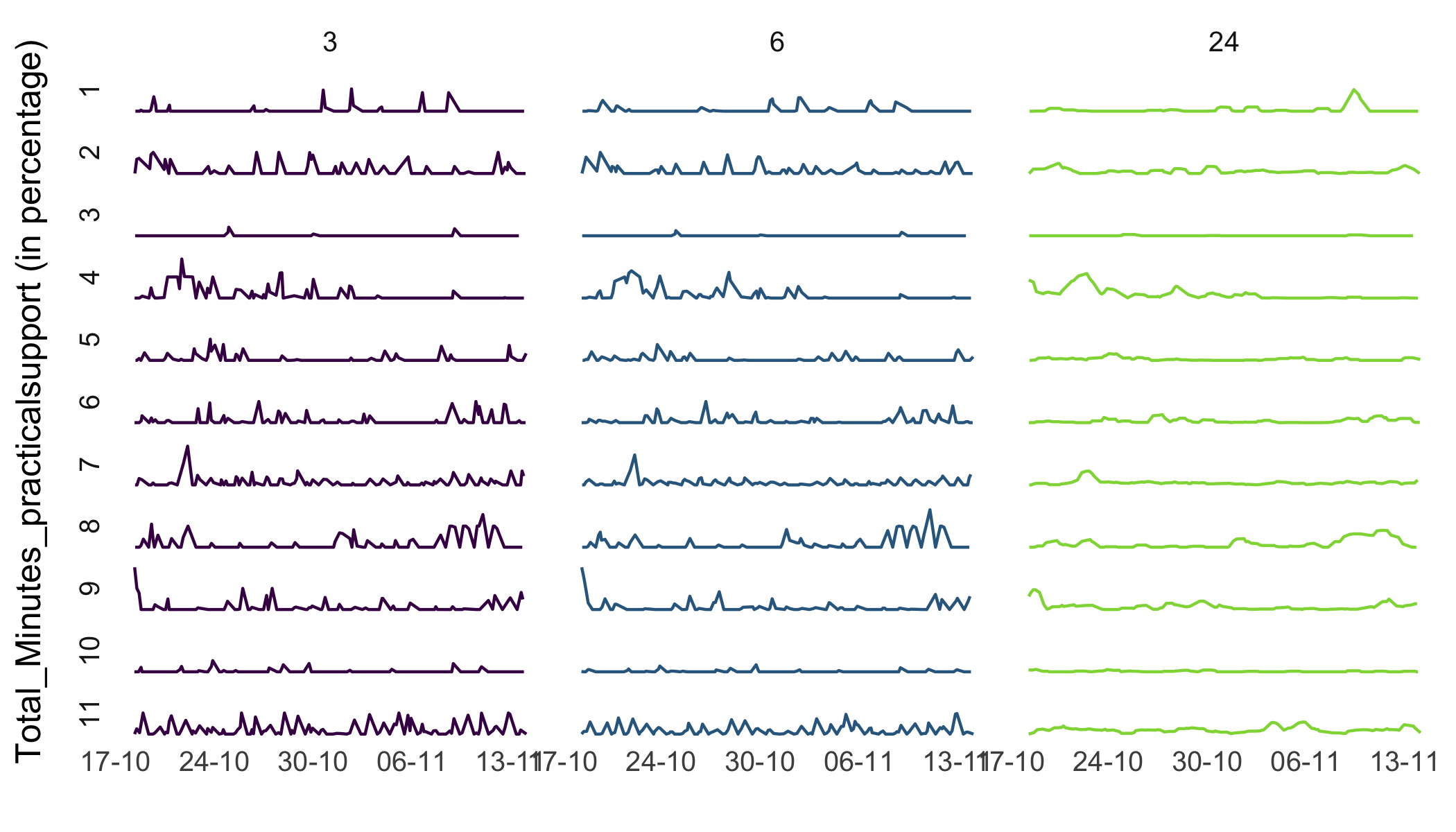


**Figure 18**

*Total Minutes App Usage (in Percentage)*
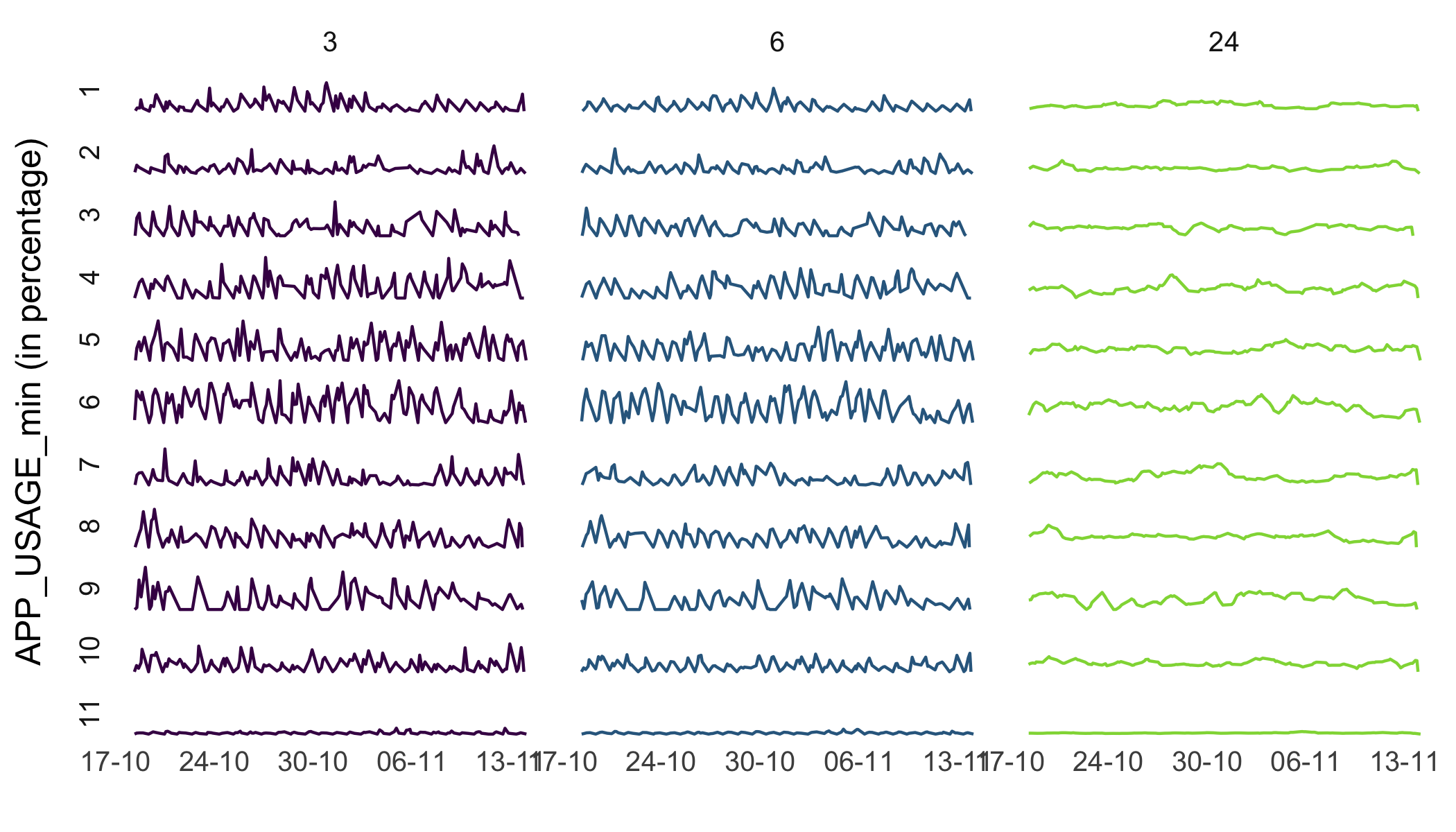


**Figure 19**

*Total Minutes Communication App Usage (in Percentage)*
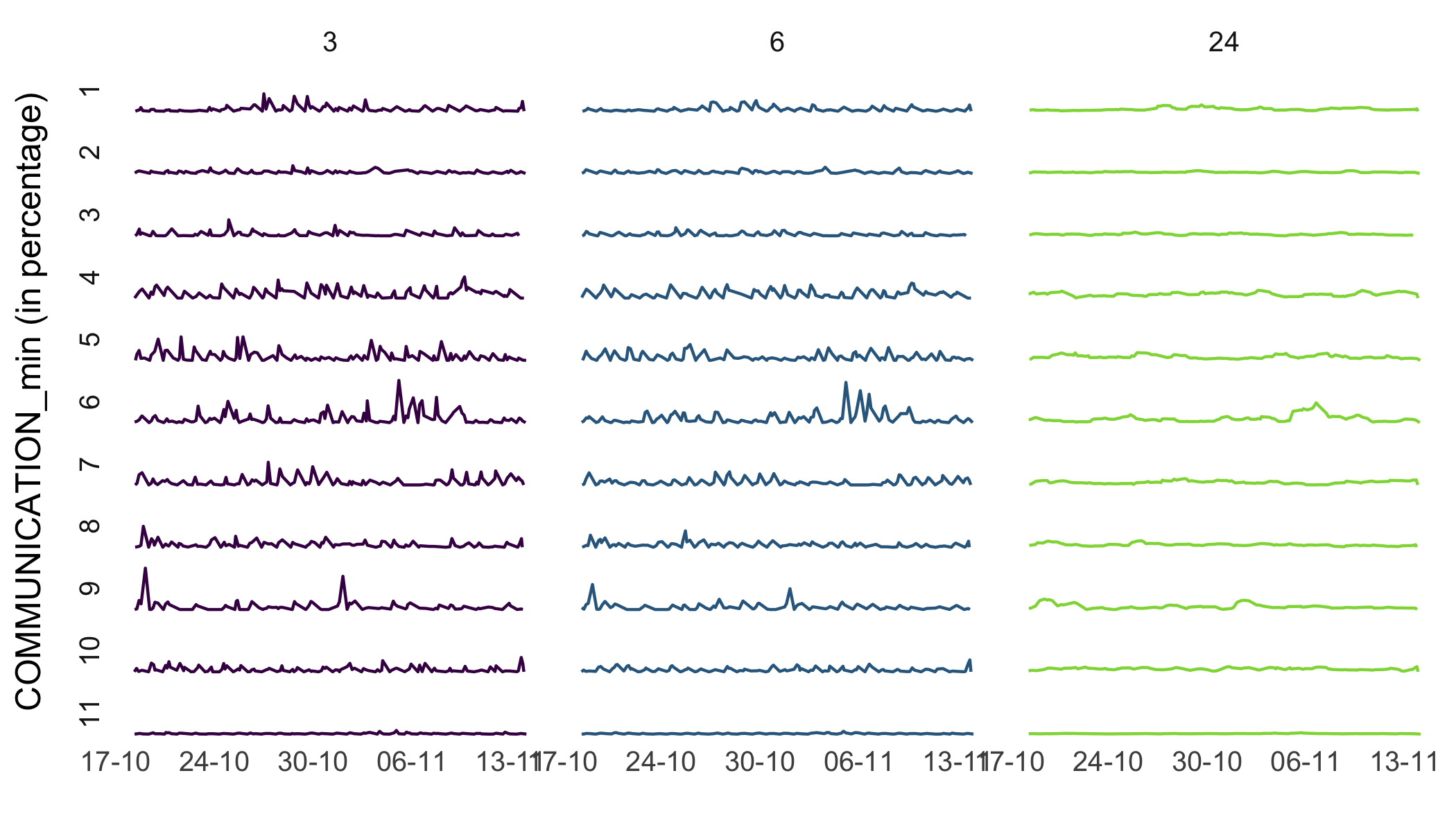


**Figure 20**

*Total Minutes Social Usage (in Percentage)*
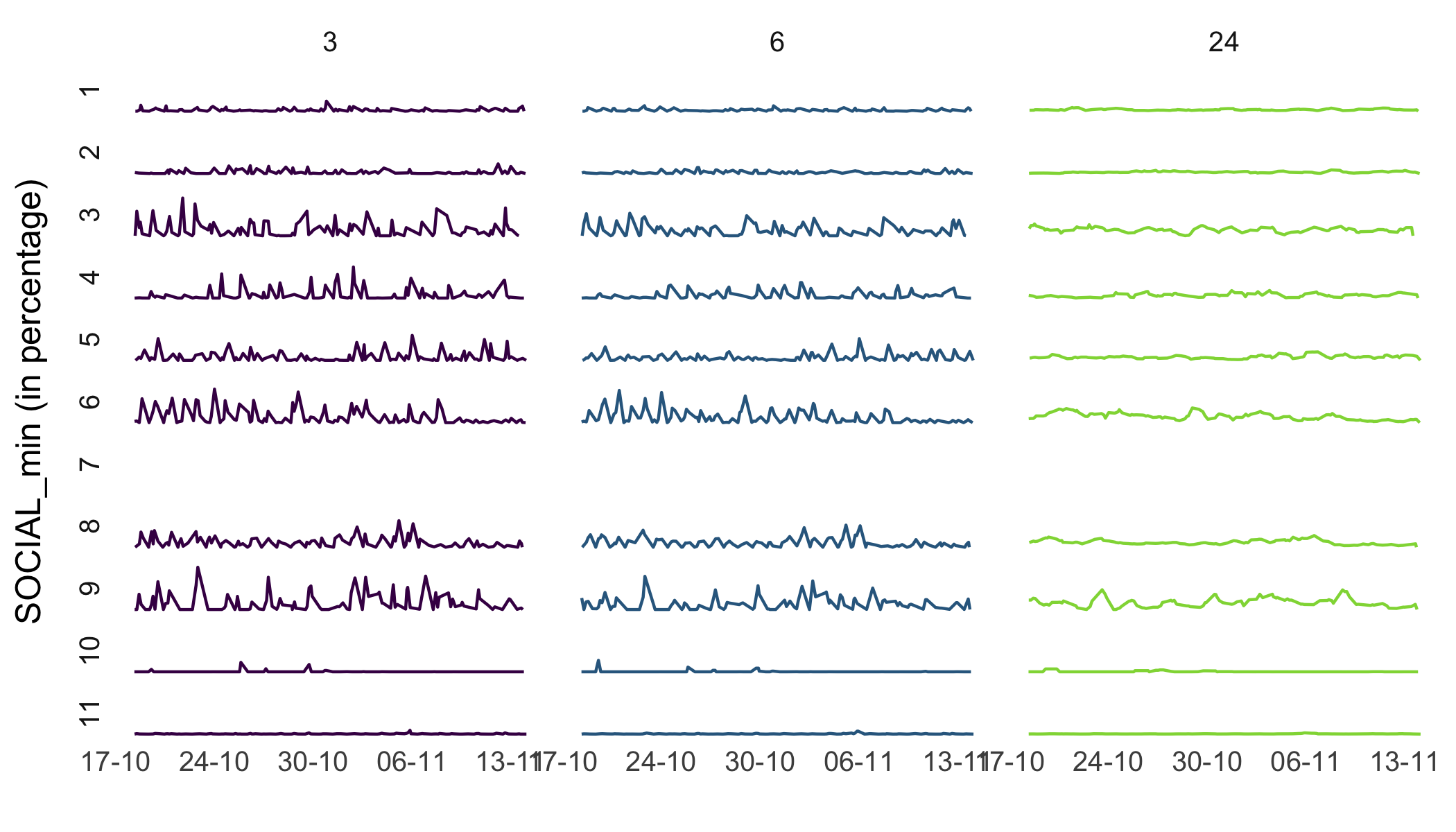


**Figure 21**

*Total Minutes Whatsapp Usage (in Percentage)*
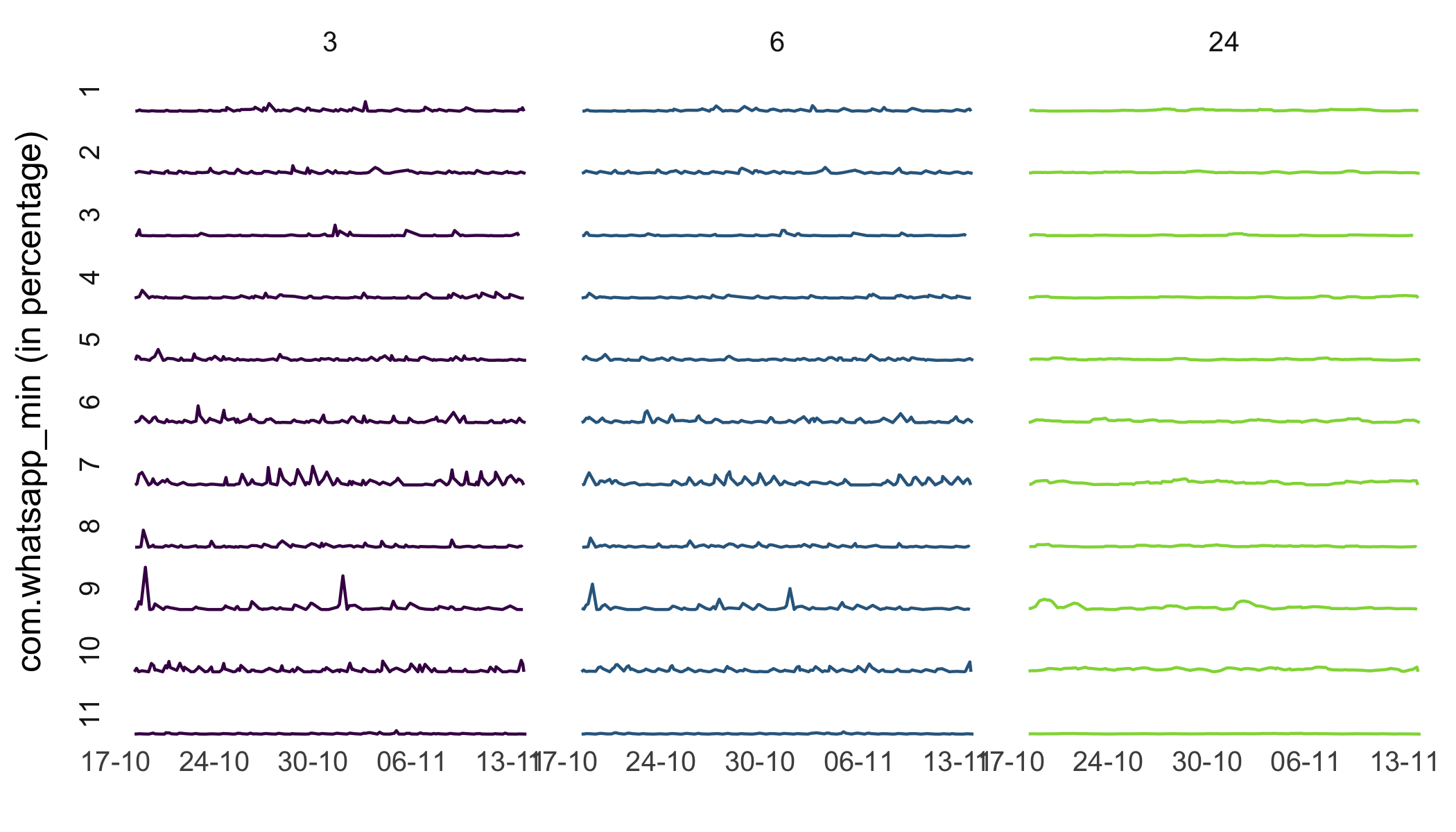


**Figure 22**

*Apps Opened (Normalized)*
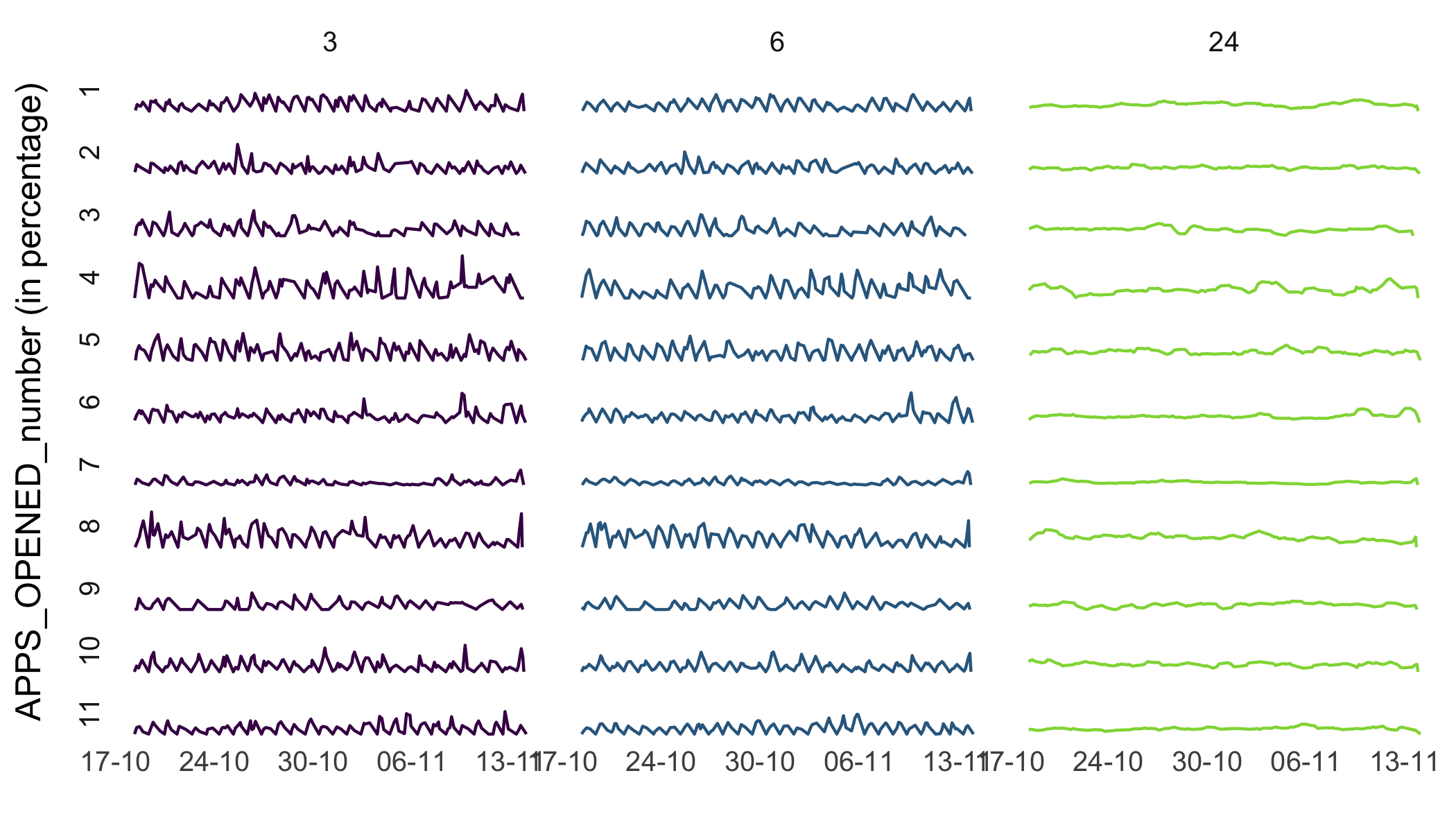


**Figure 23**

*Unique Staypoints (Normalized)*


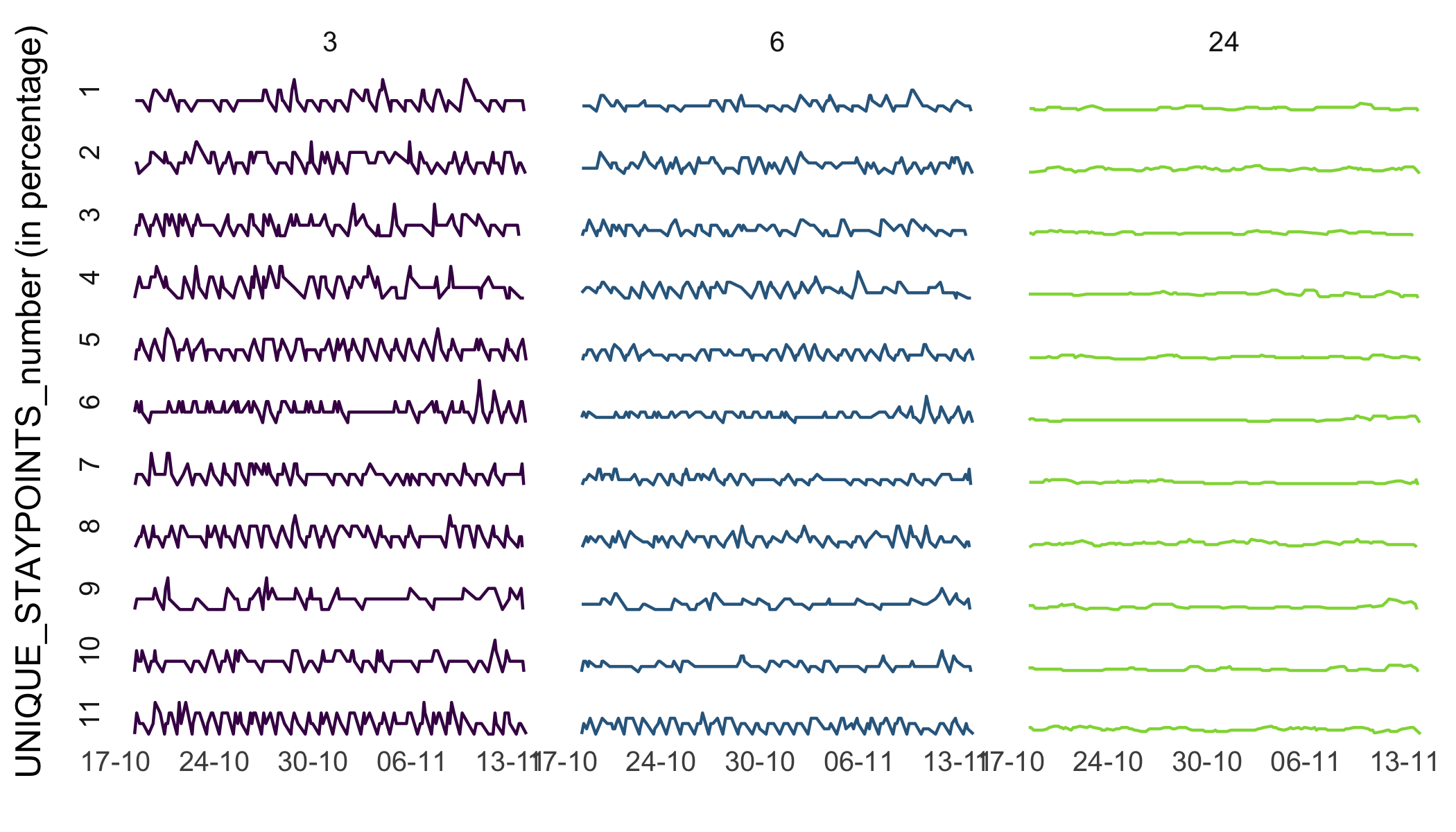


**Figure 24**

*Minutes Spent Home (in Percentage)*
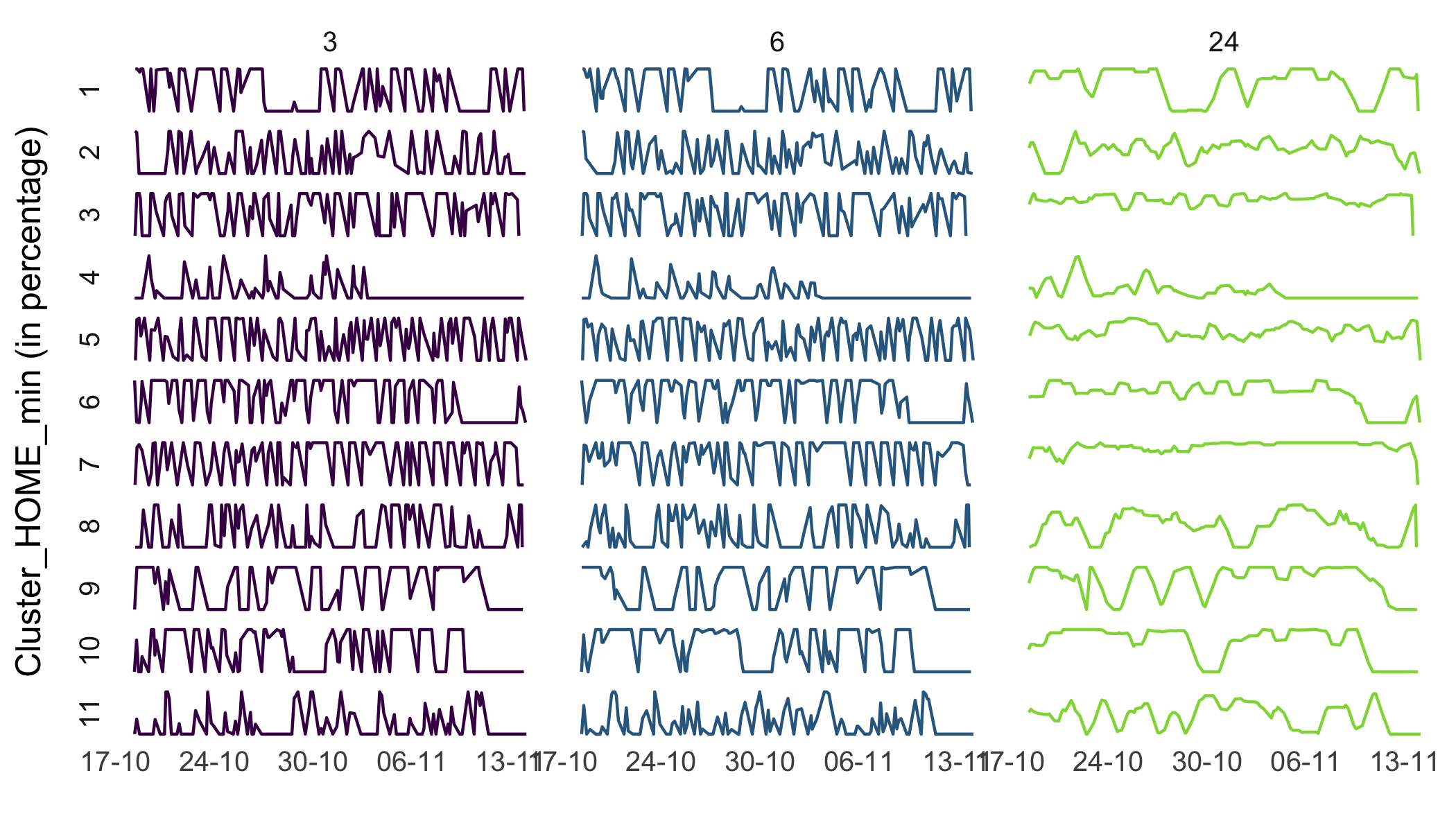


**Figure 25**

*Minutes Spent Stationary (in Percentage)*
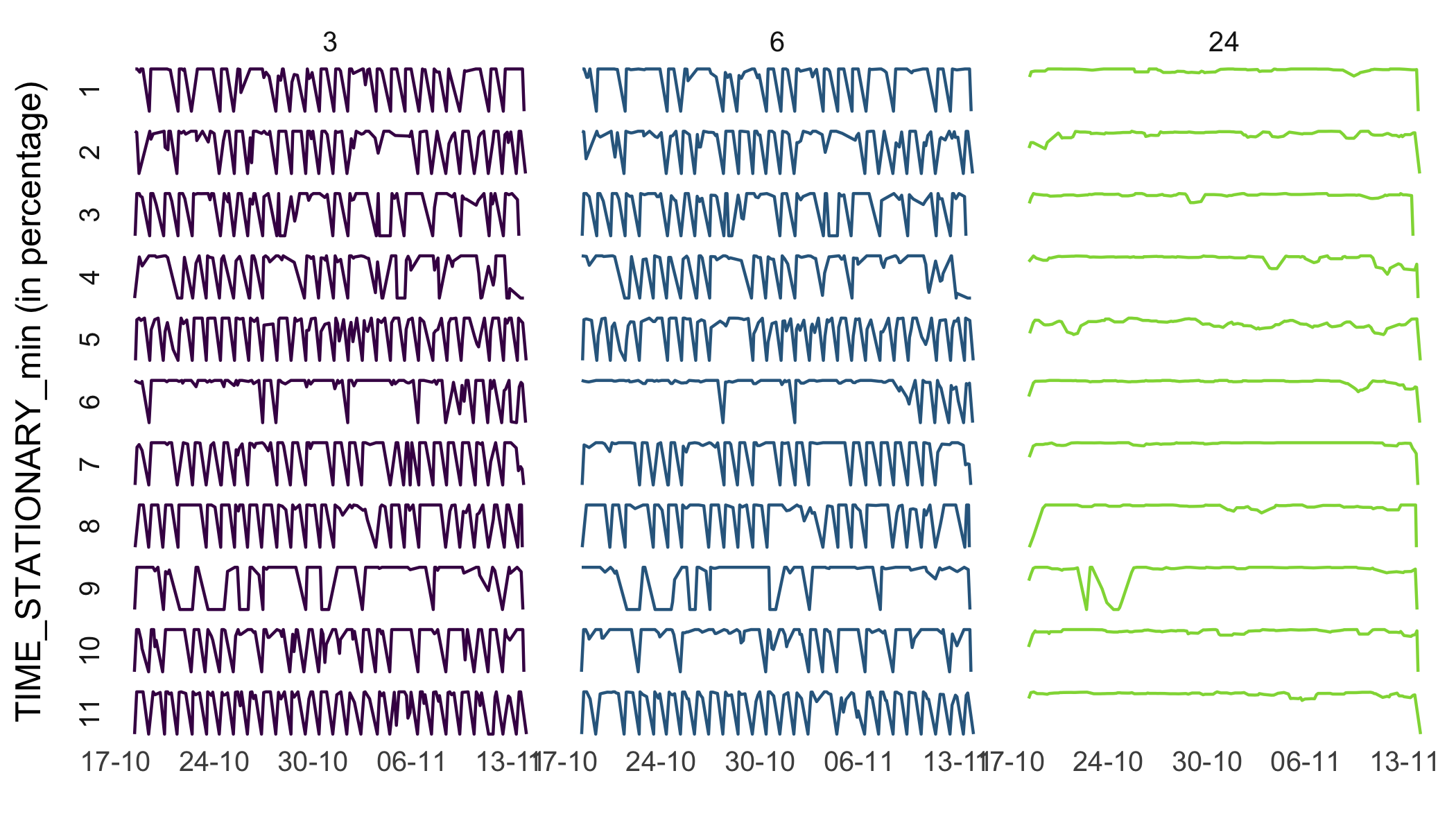


**Figure 26**

*Average Distance Travelled from Home (Normalized)*
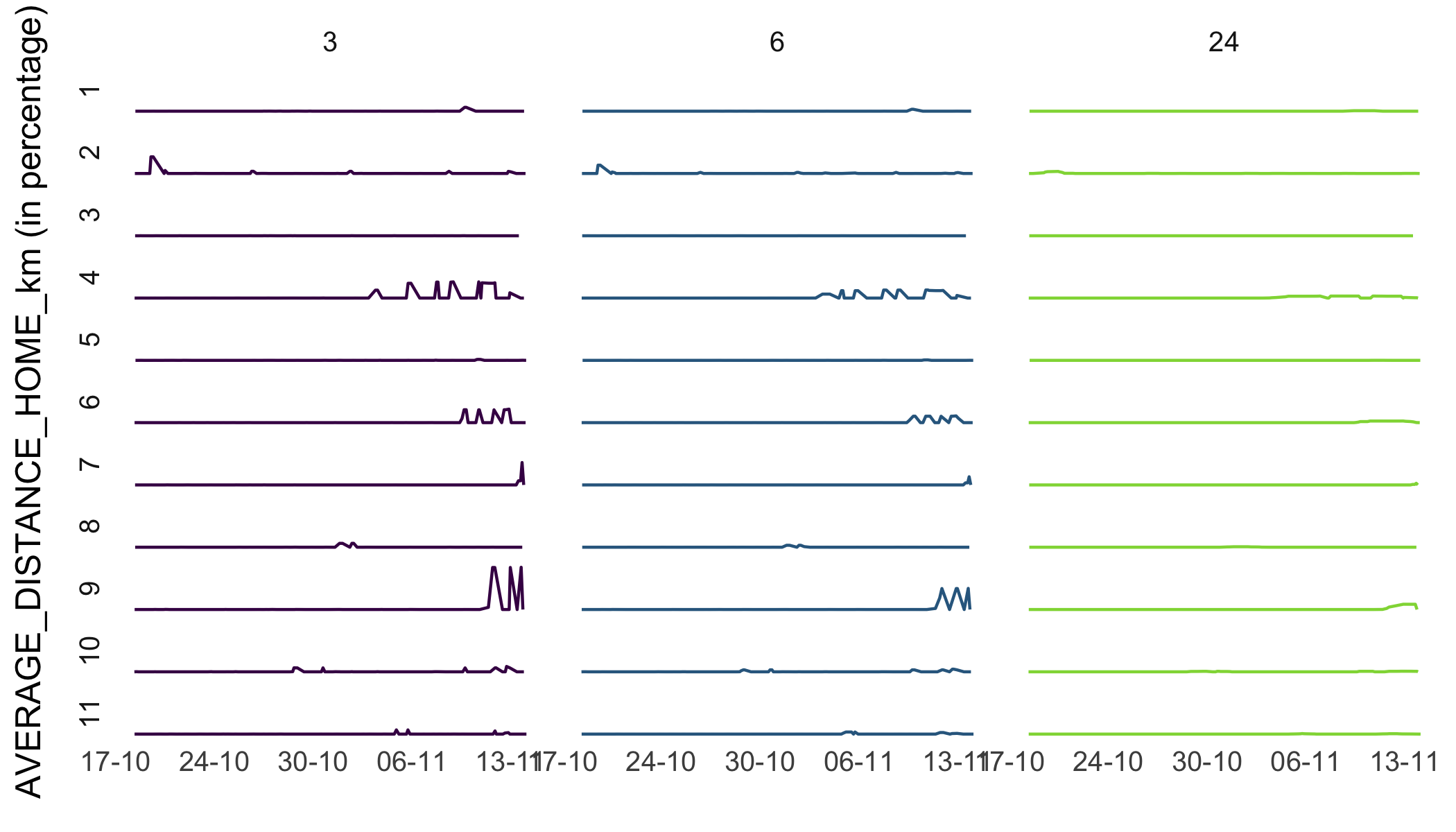


**Figure 27**

*Call Total Minutes (in Percentage)*
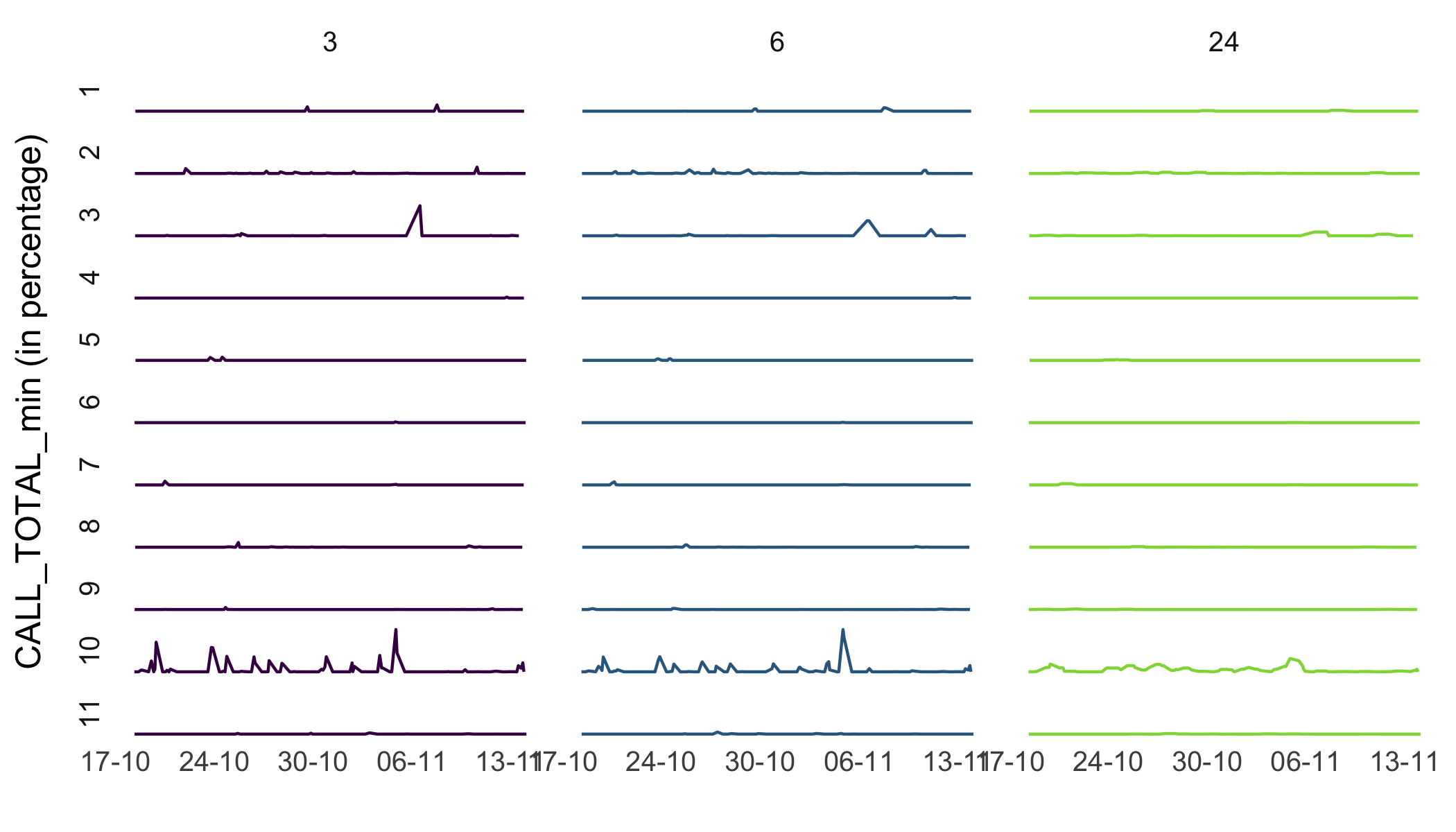


**Figure 28**

*Incoming Calls Total Minutes (in Percentage)*
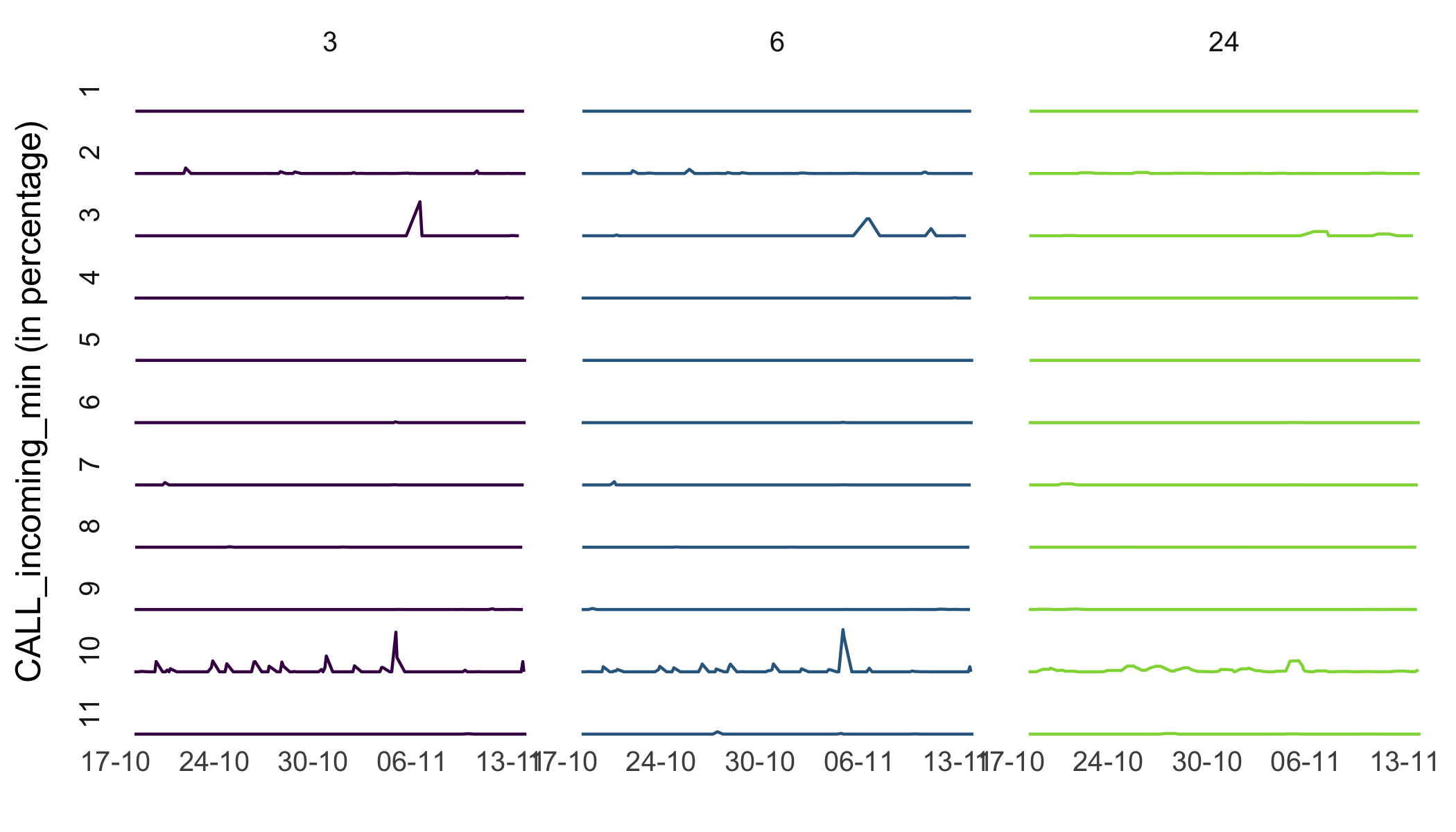


**Figure 29**

*Outgoing Calls Total Minutes (in Percentage)*
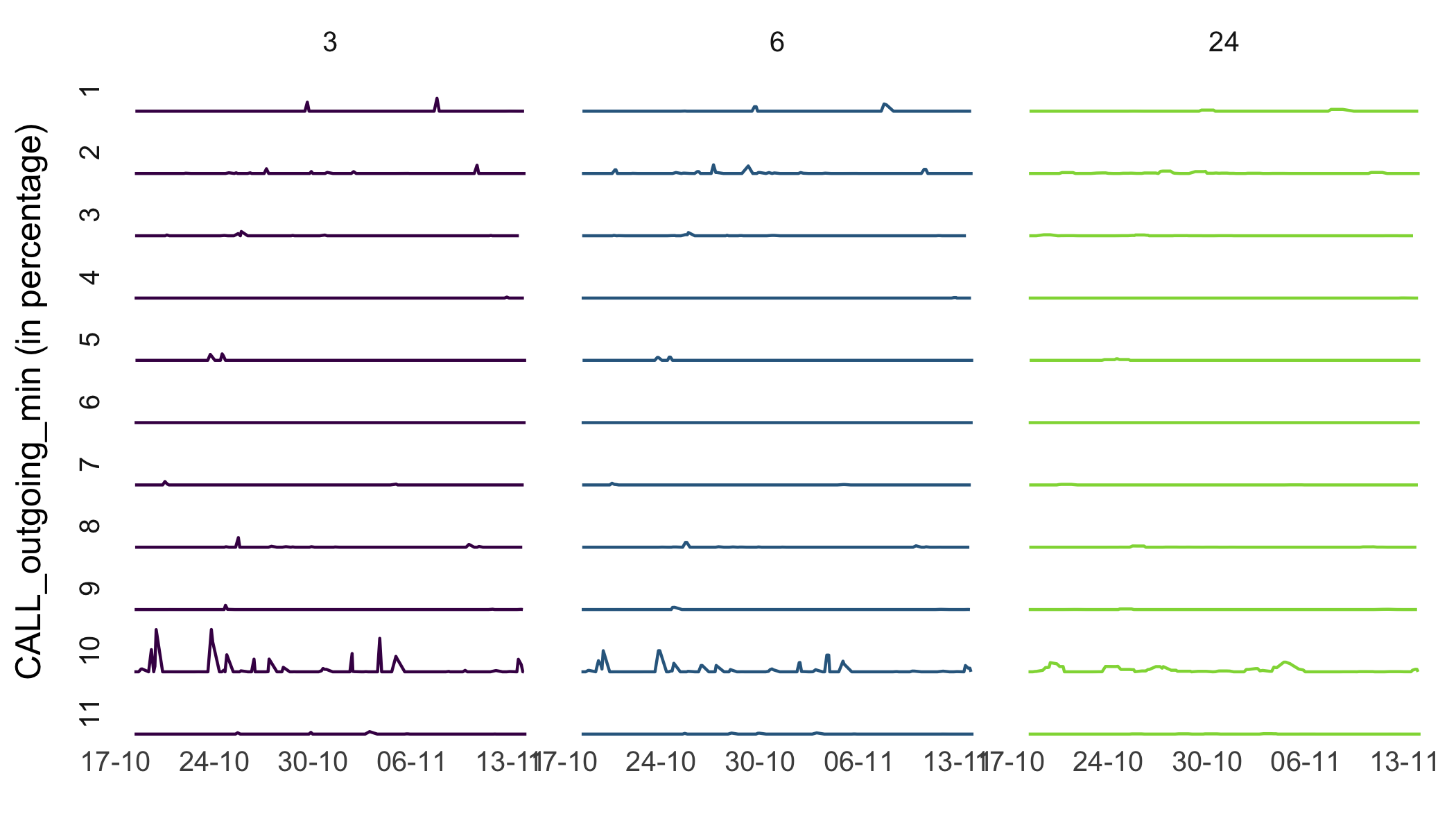


**Figure 30**

*Total Wi-Fi Connections (Normalized)*
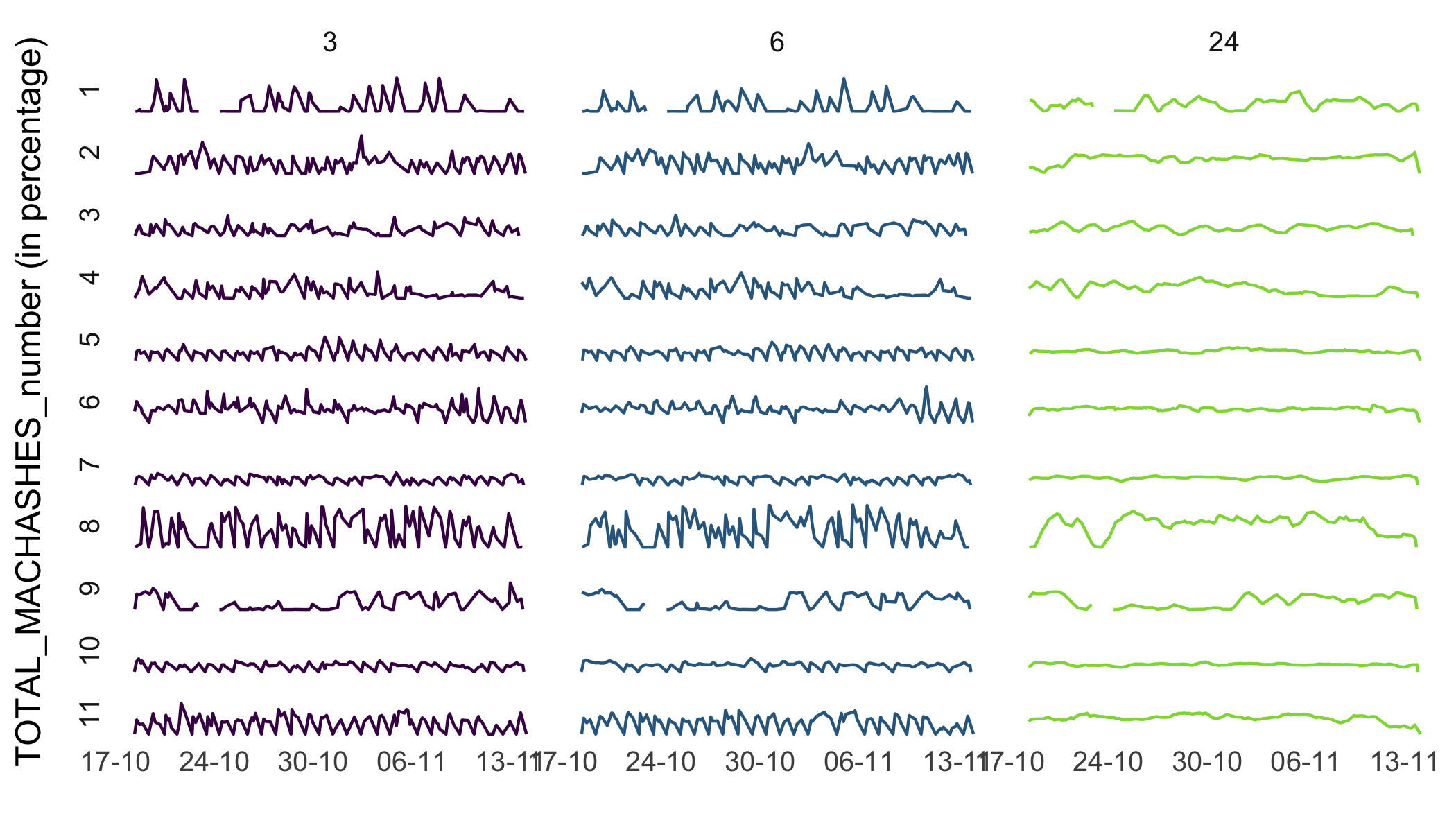


**Figure 31**


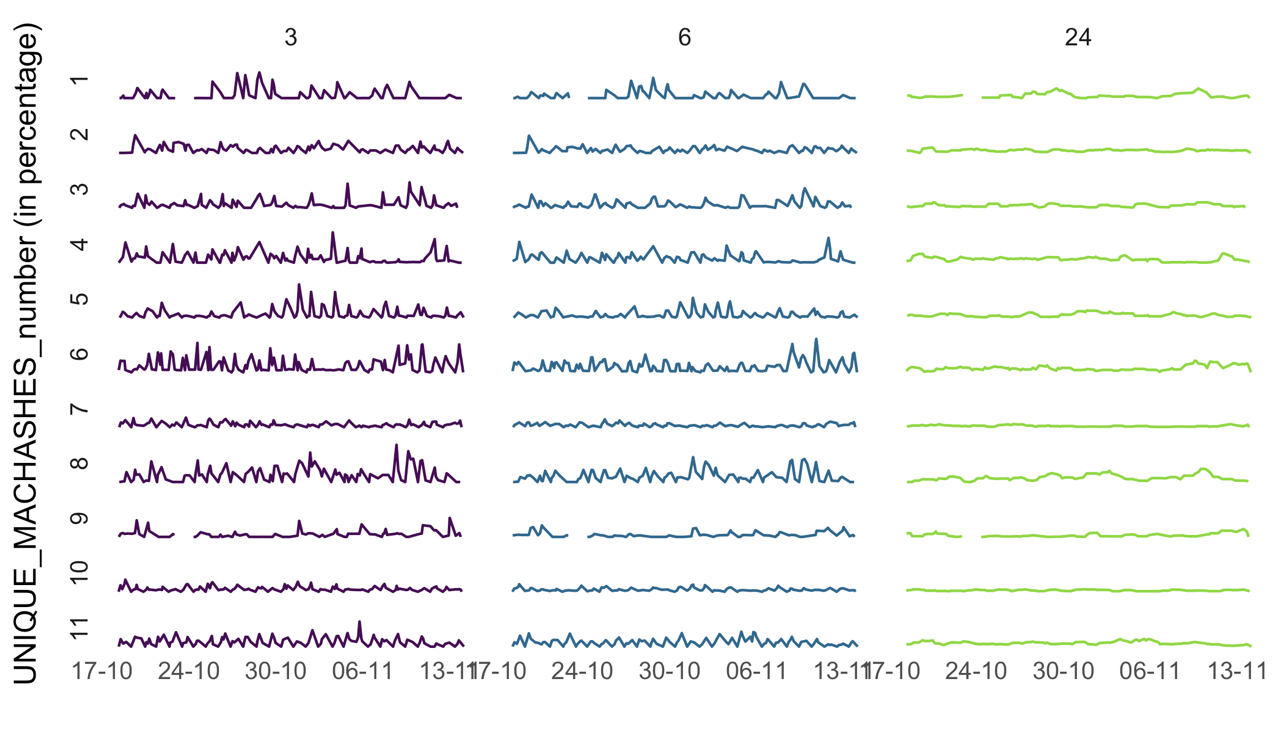
*Unique Wi-Fi Connections (Normalized)*

**Figure 32**

*Times Screen On Locked (Normalized)*
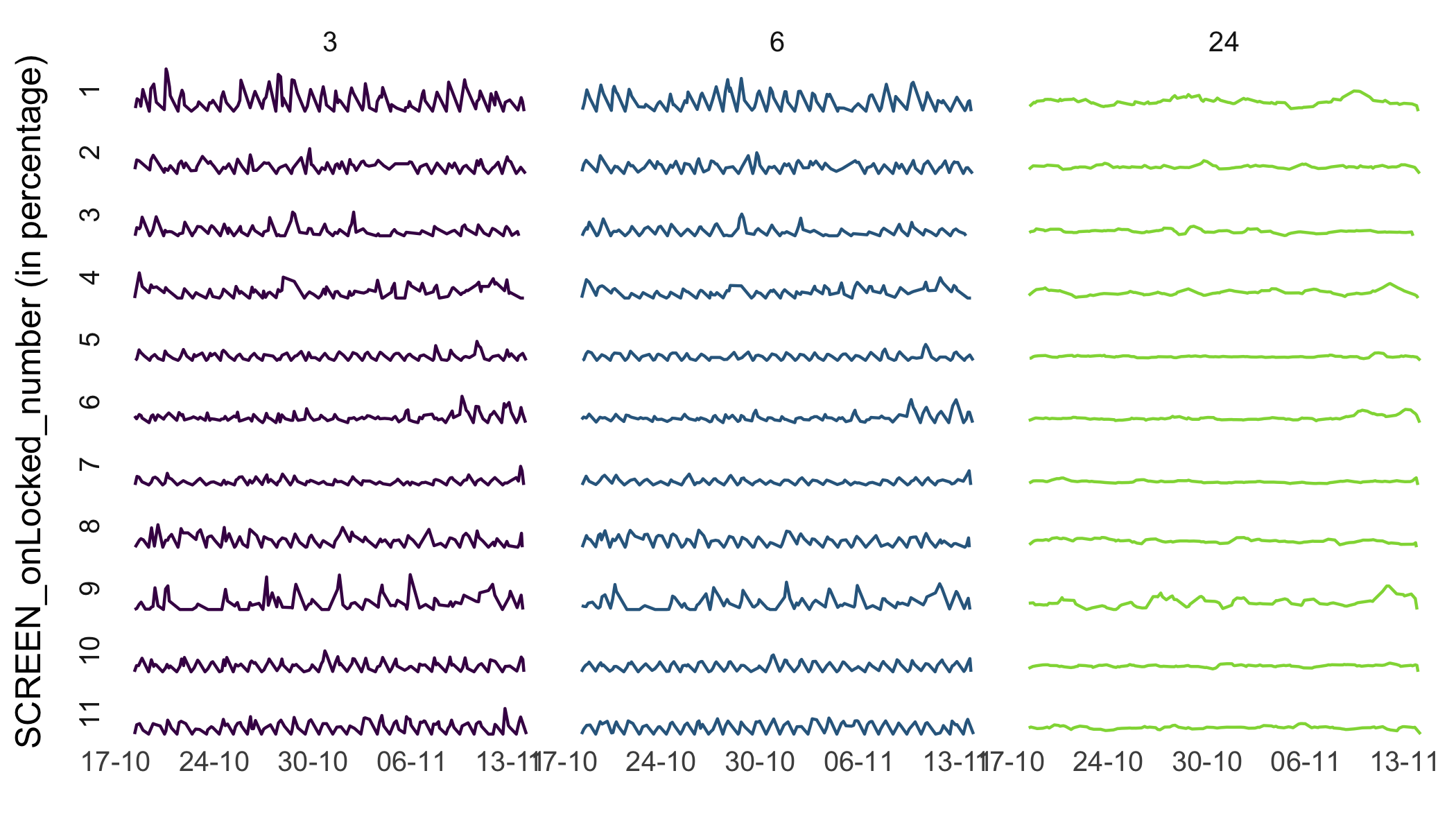


**Figure 33**

*Times Screen On Unlocked (Normalized)*


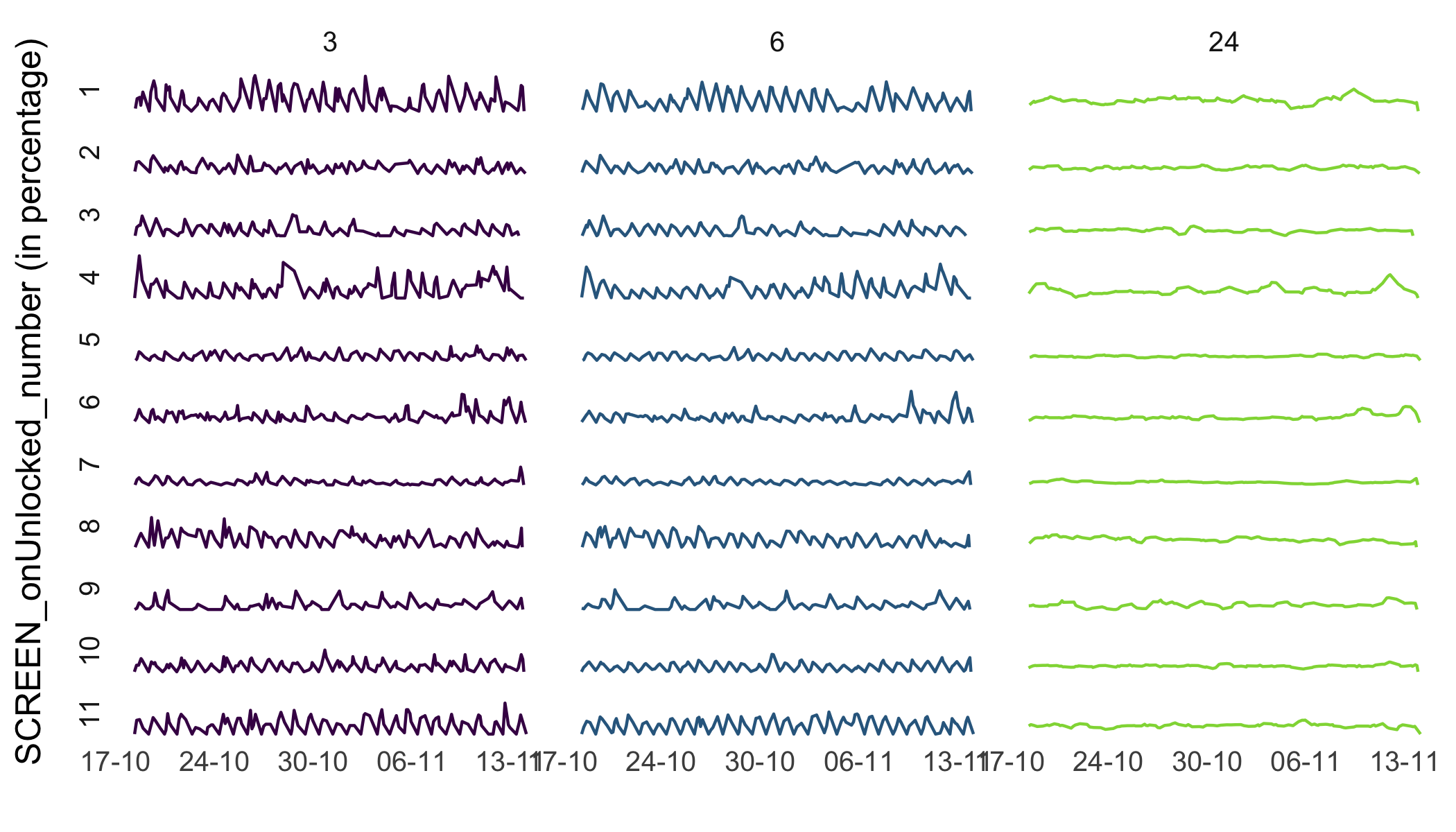


**Figure 34**

*Last Interaction Enjoy* *(Mean)*


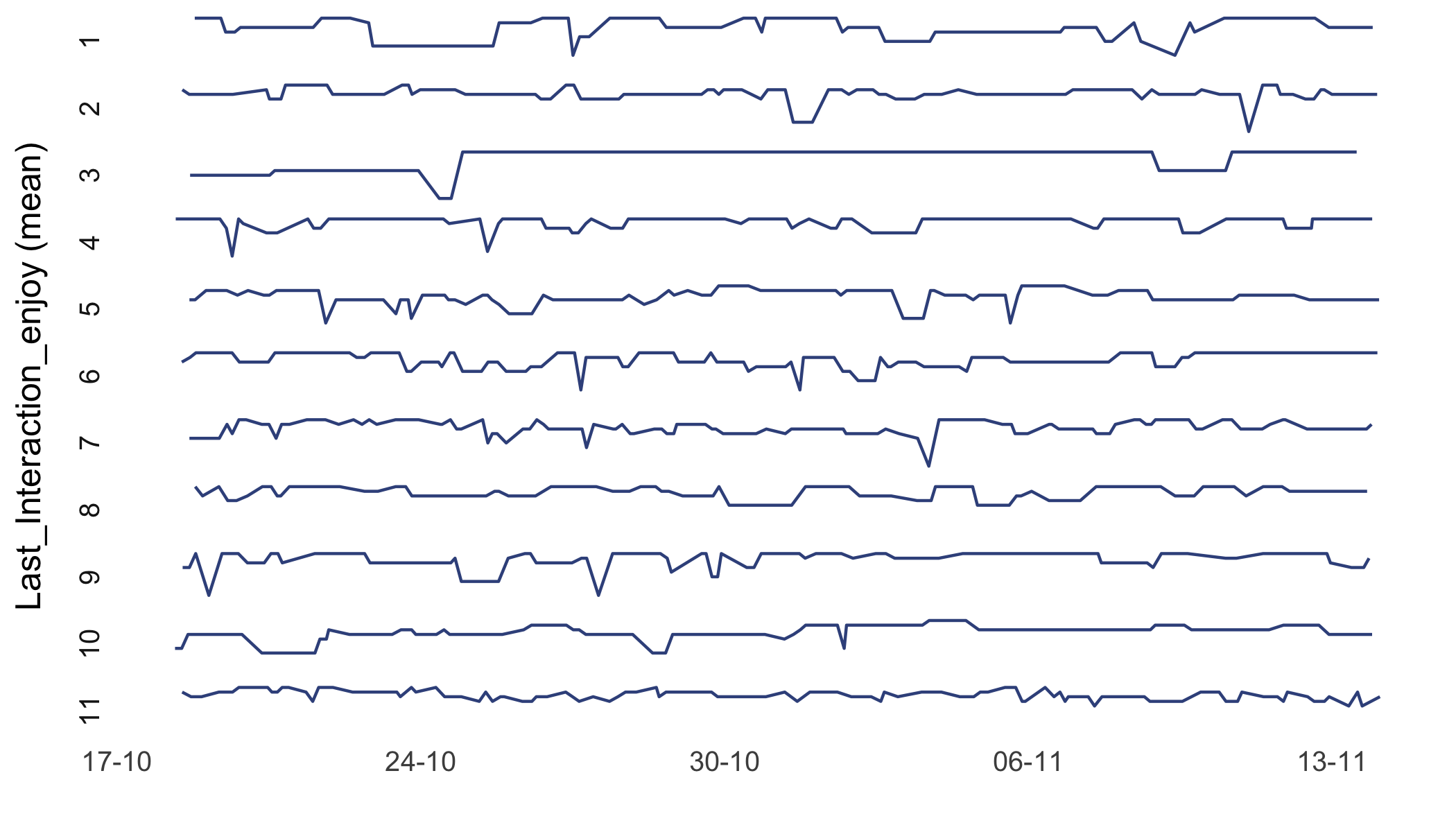


**Figure 35**

*Last Interaction Other Enjoy* *(Mean)*


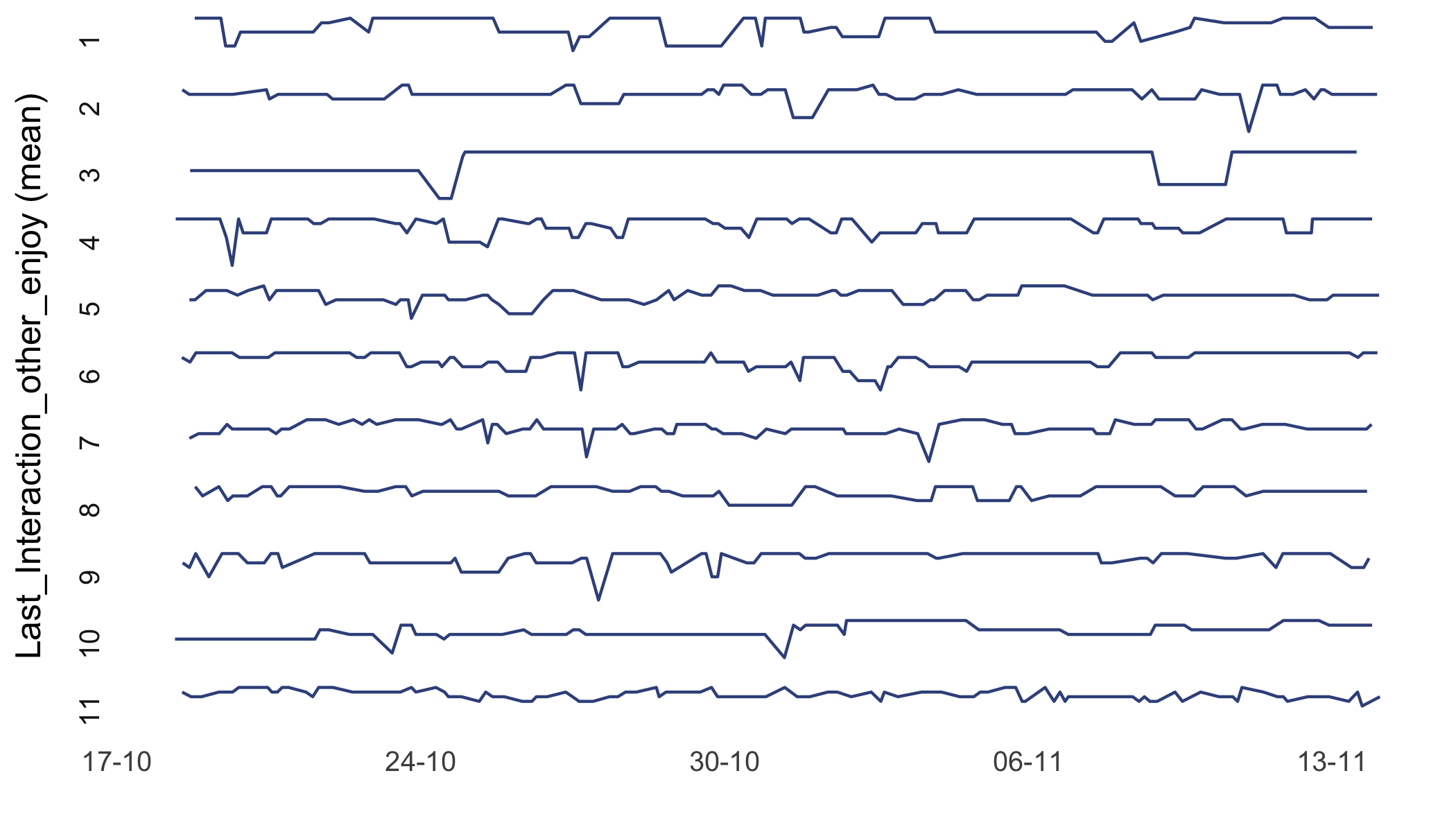


**Figure 36**

*Last Interaction Meaningful* *(Mean)*


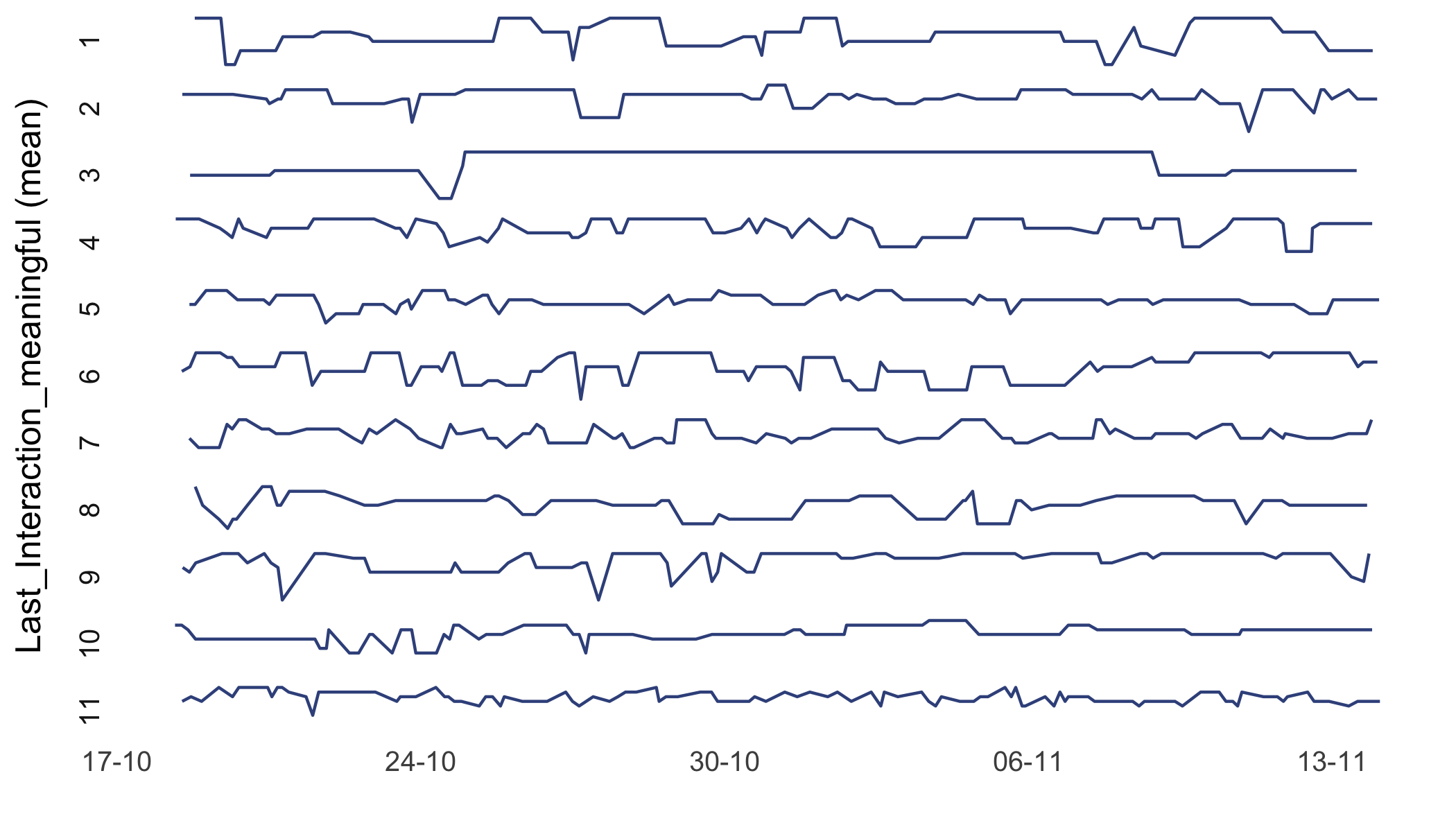


**Figure 37**

*Last Interaction Could Be Myself* *(Mean)*


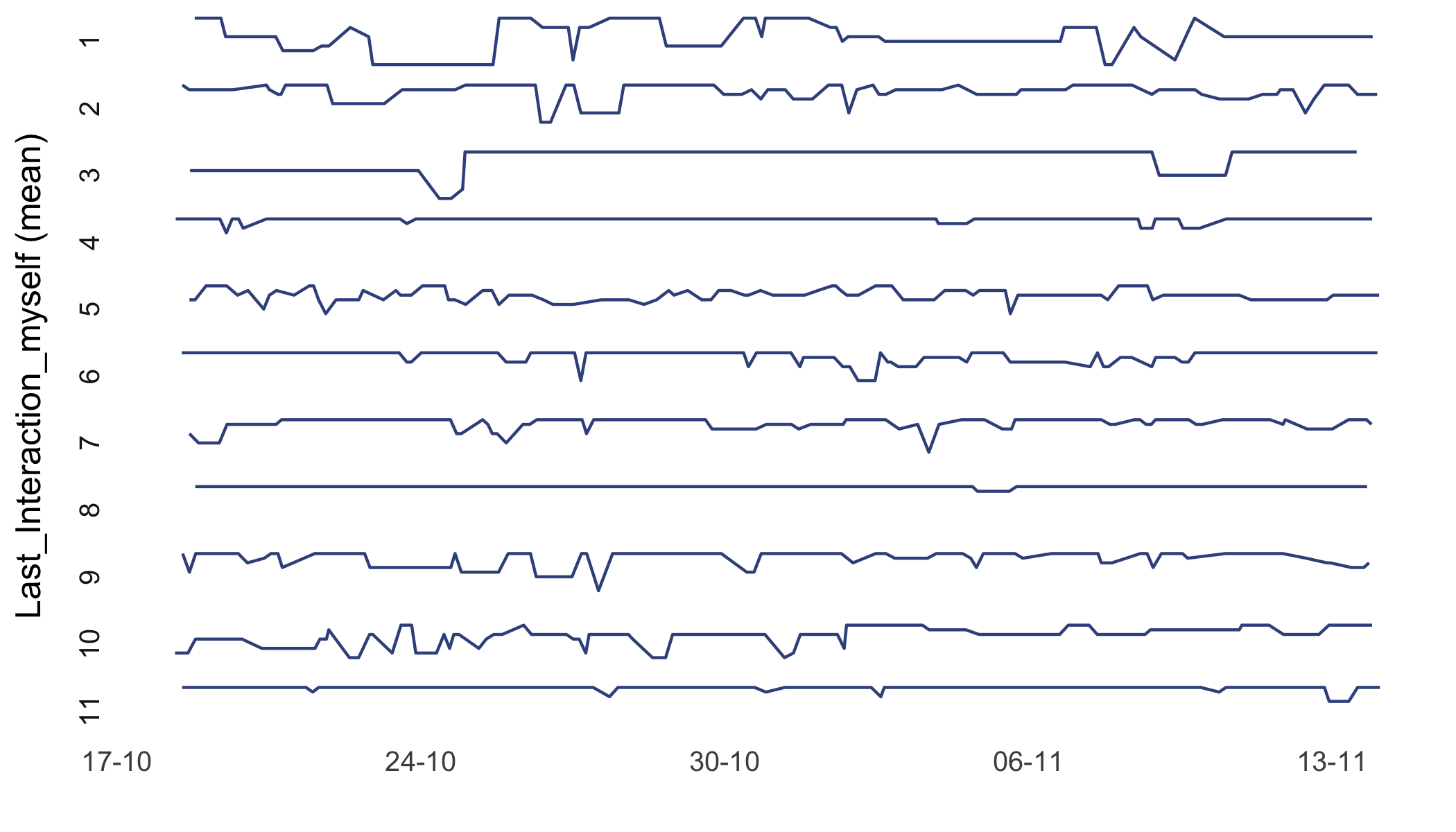


**Figure 38**

*Last Interaction Cost Energy* *(Mean)*


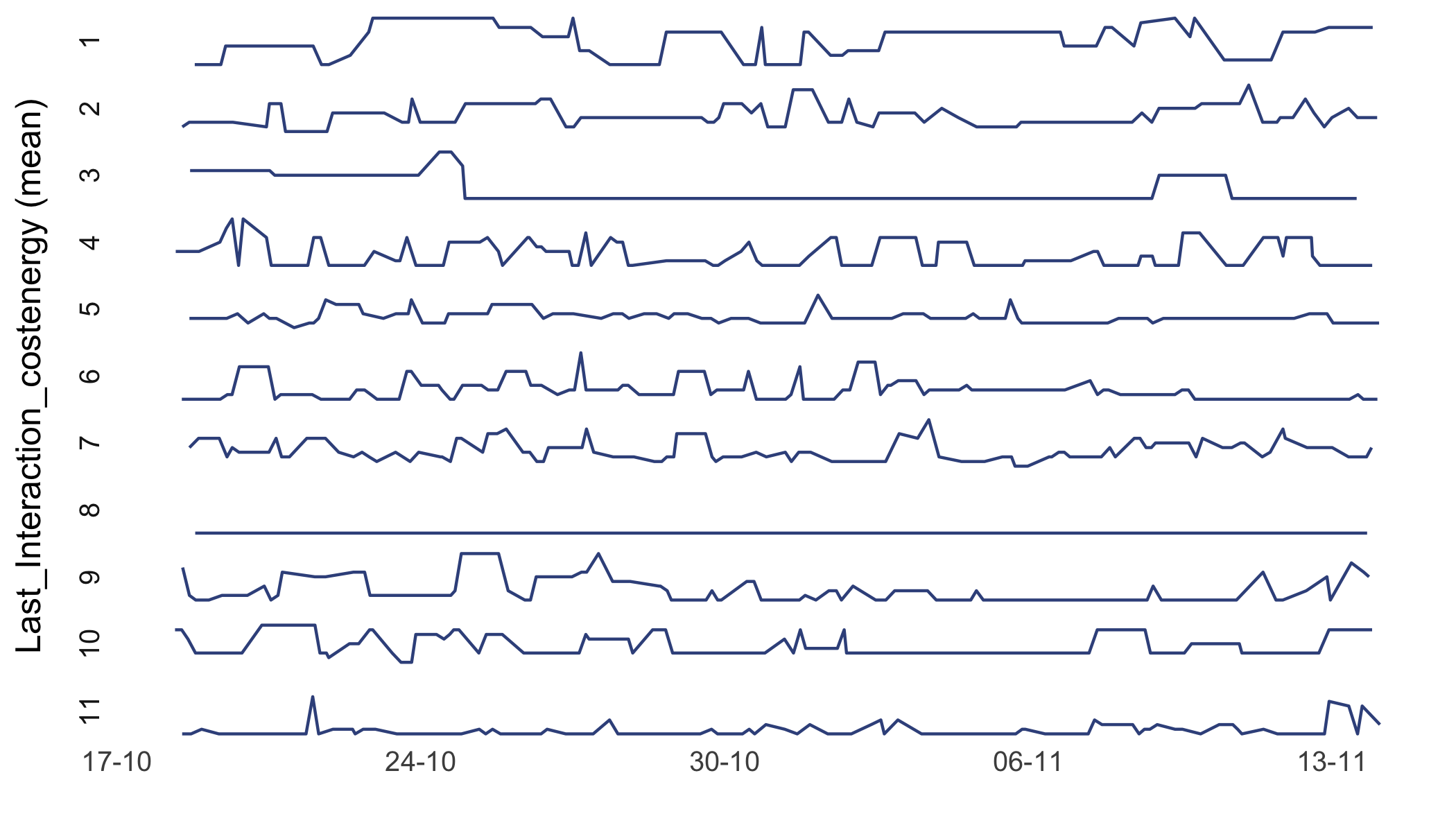


**Figure 39**

*Last Interaction Gives Energy* *(Mean)*


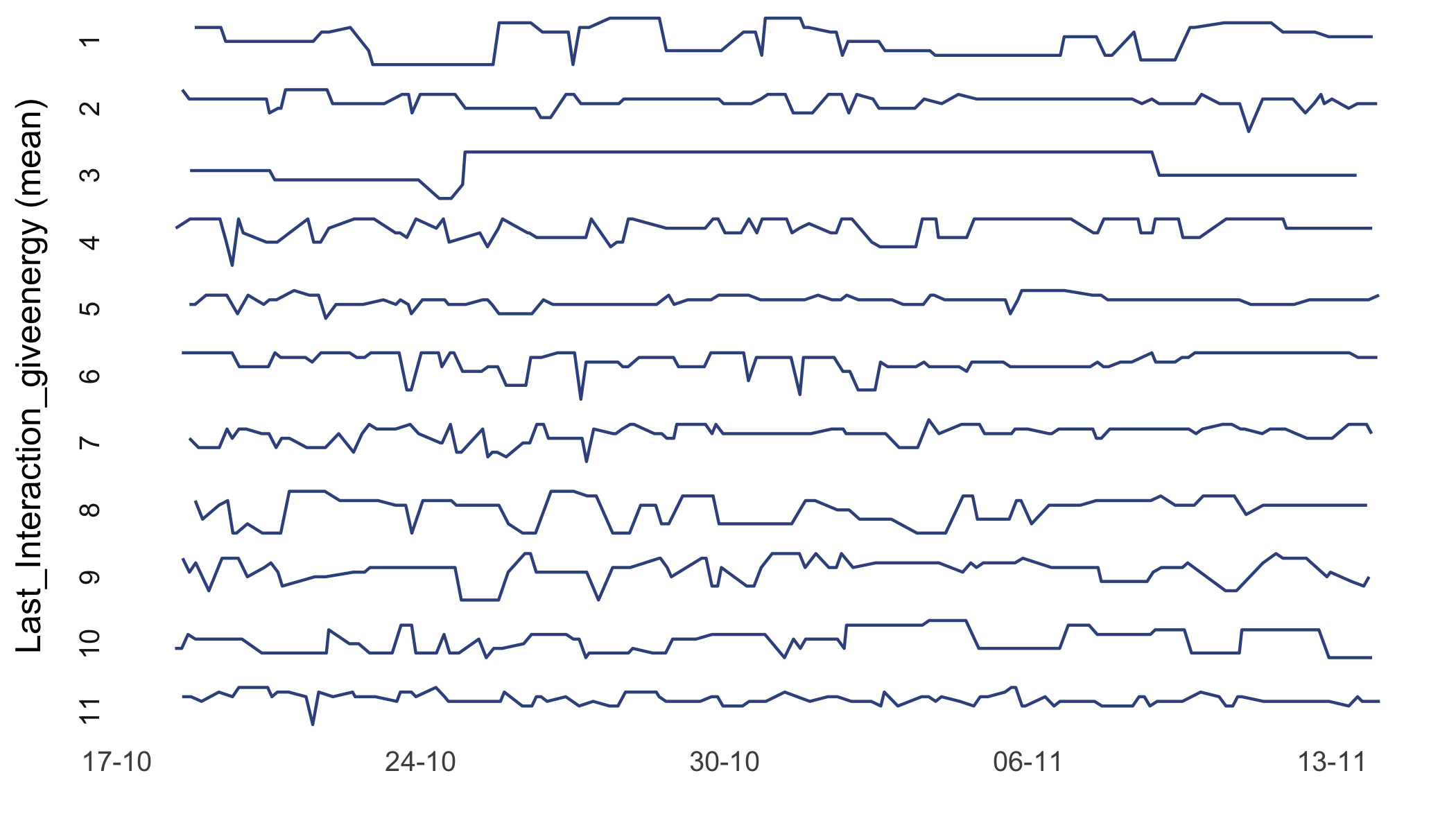


**Figure 40**

*Last Interaction Happy* *(Mean)*


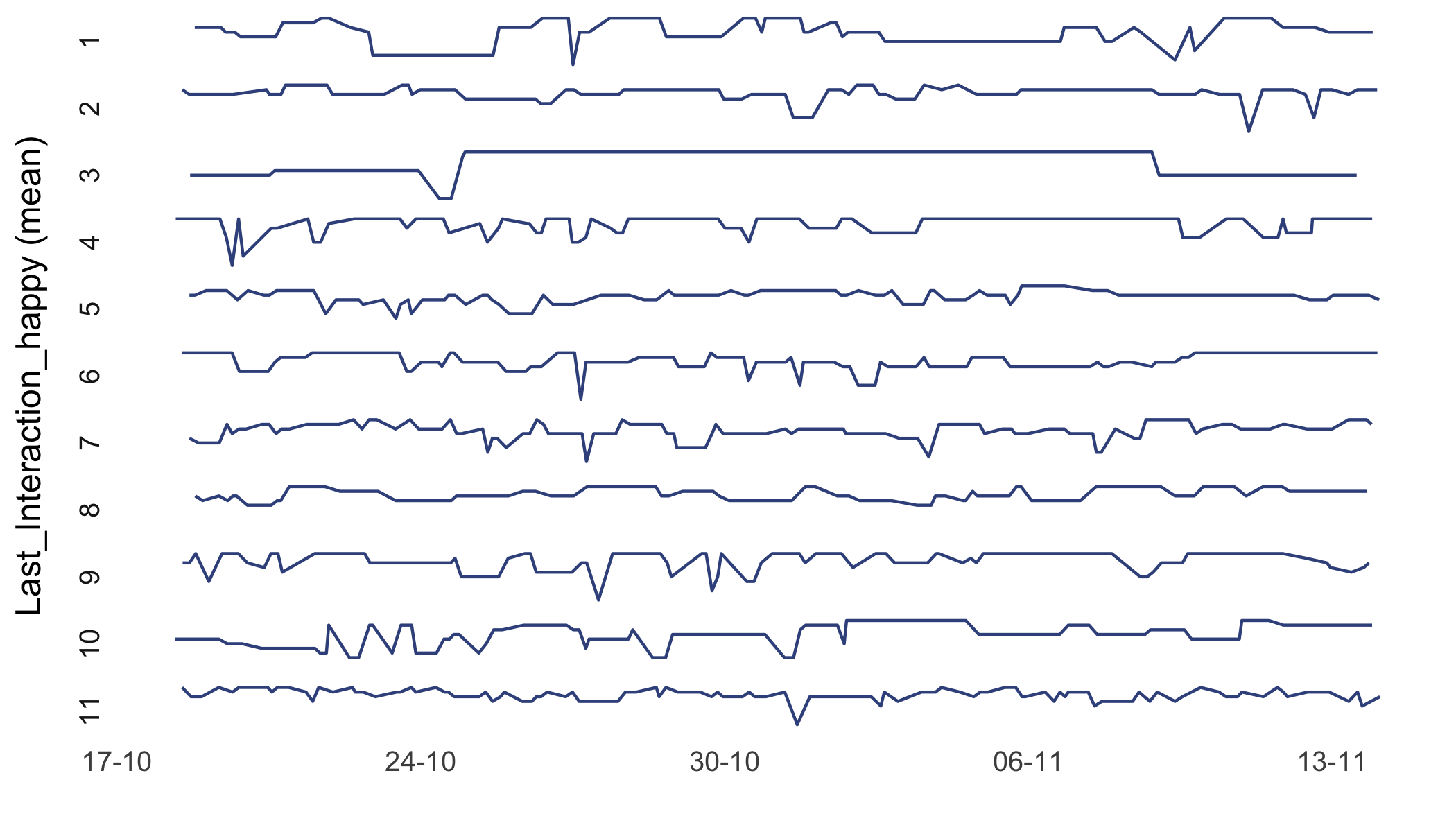


**Figure 41**

*Last Interaction Time* *(Mean)*


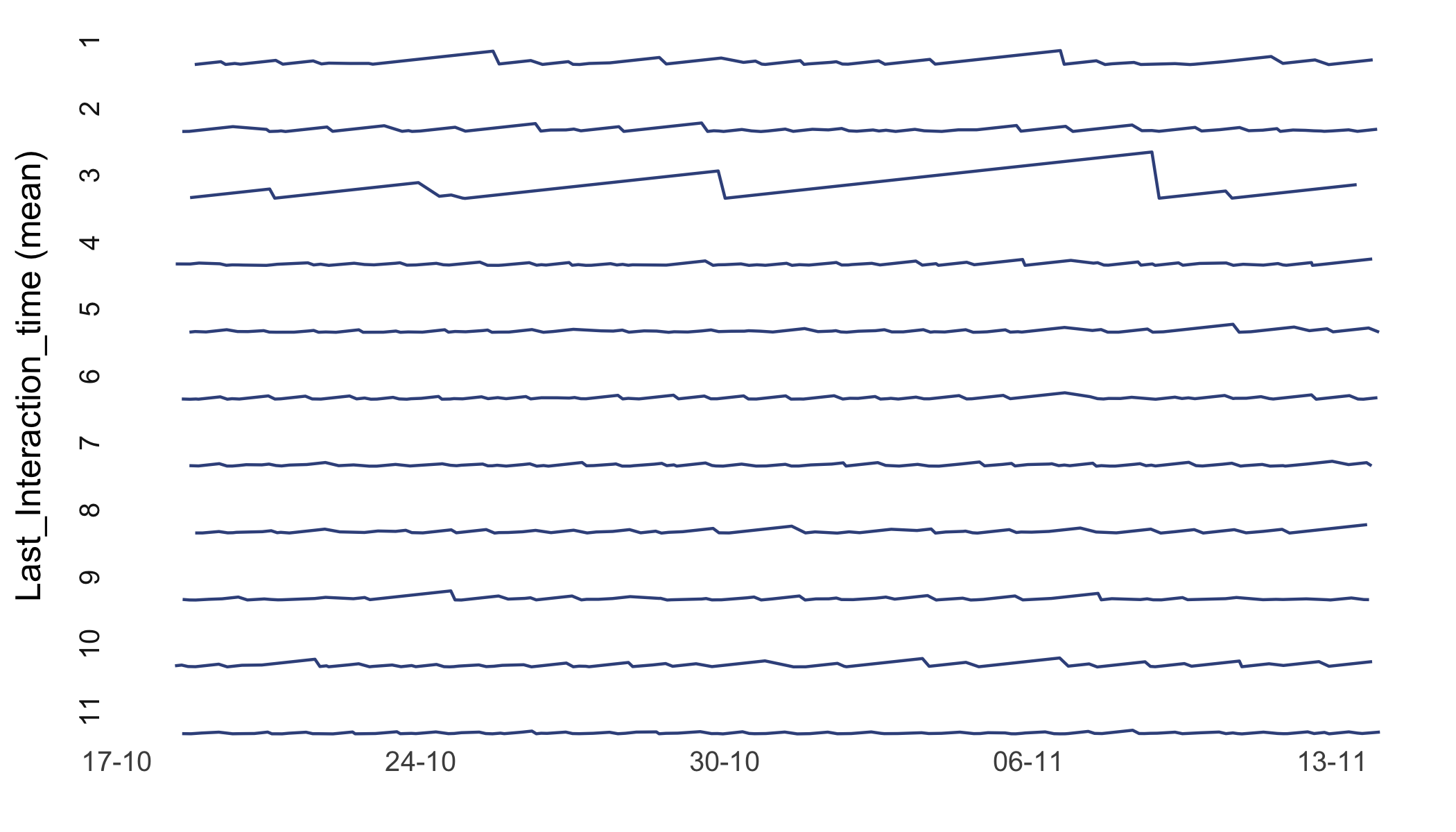


**Figure 42**

*Last Interaction Closeness to Interaction Partner* *(Mean)*


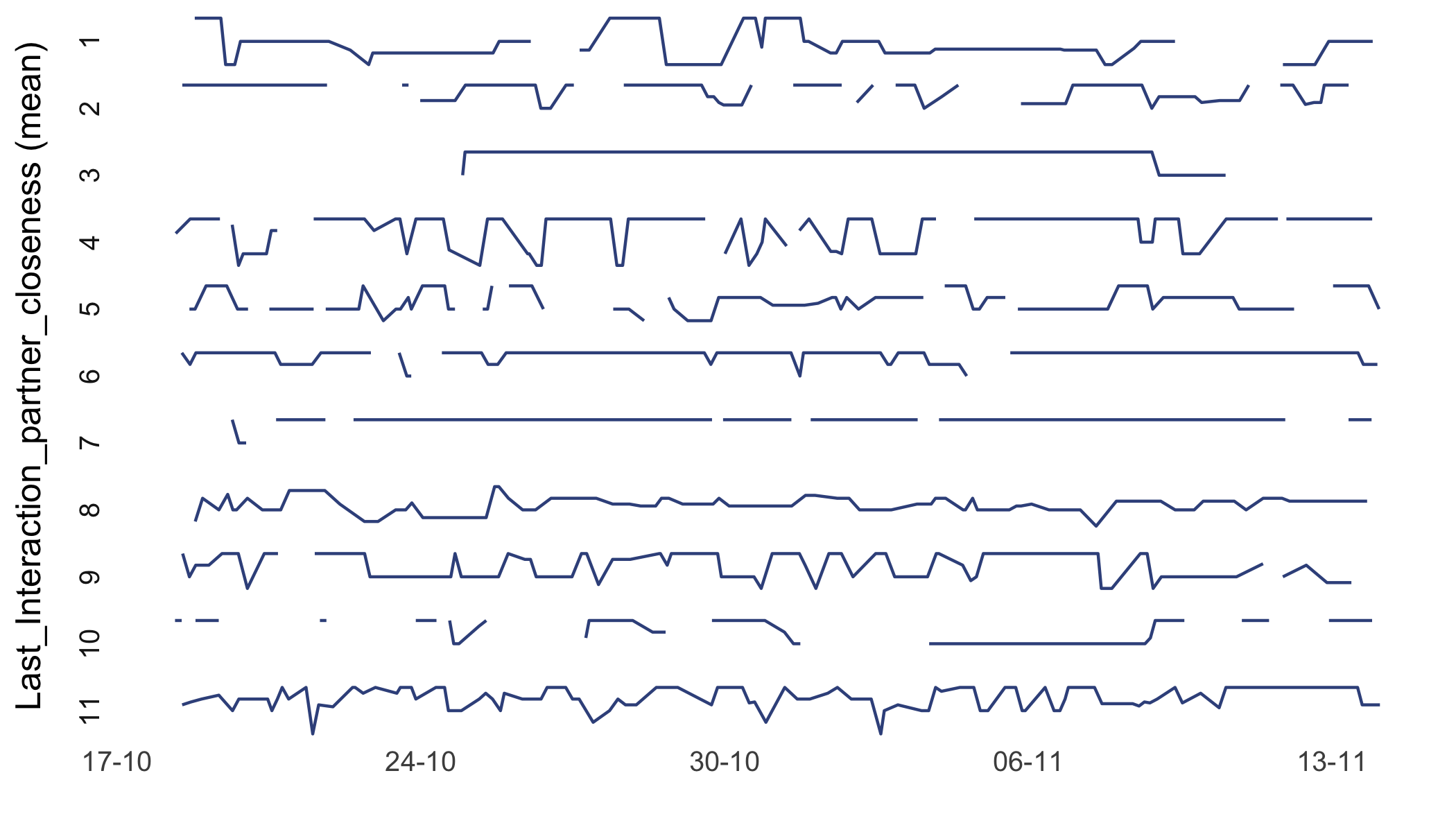


**Figure 43**

*Last Interaction, Interaction Partner* *Gives Energy* *(Mean)*


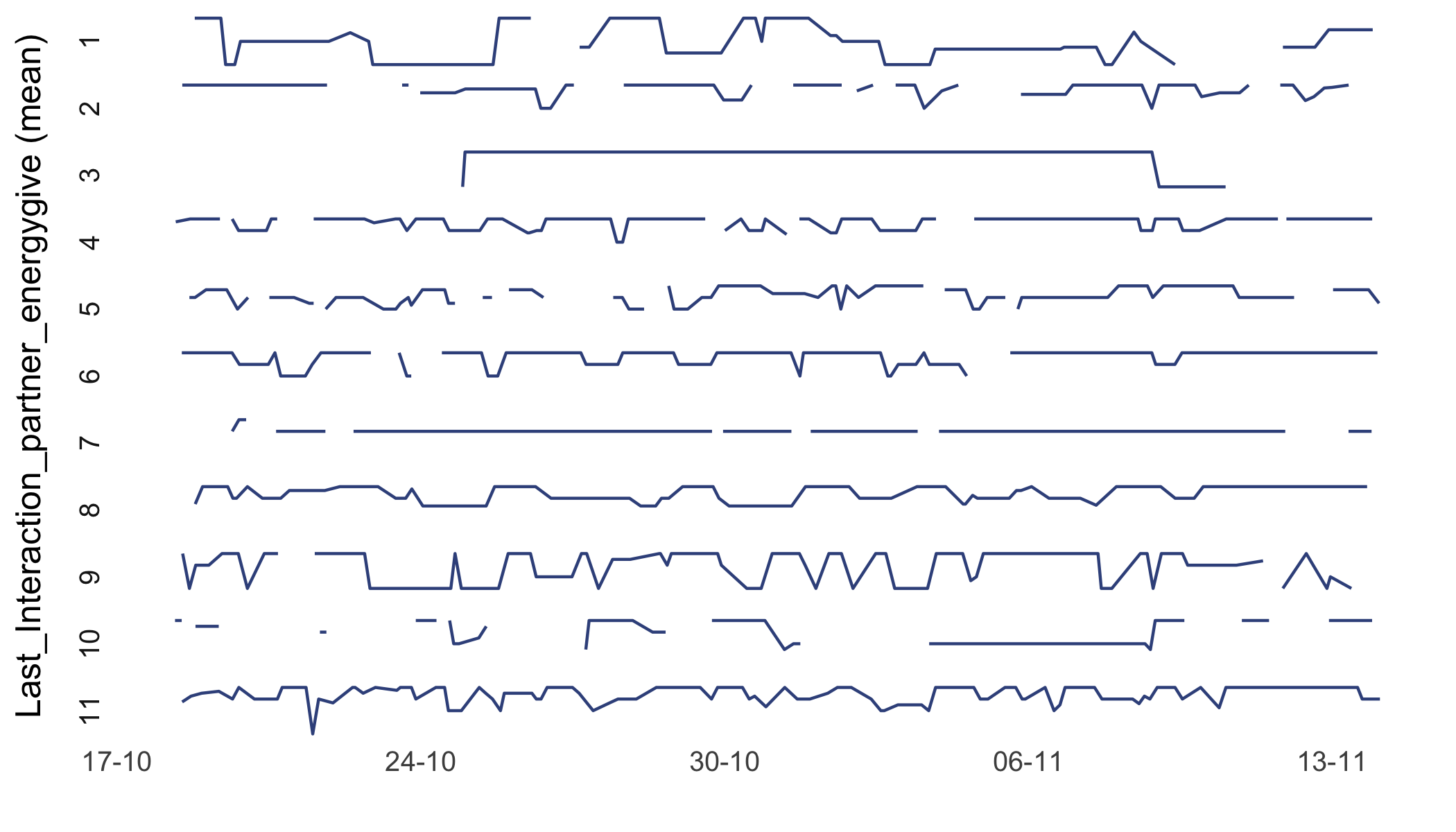


**Figure 44**

*Last Interaction, Interaction Partner* *Cost Energy* *(Mean)*


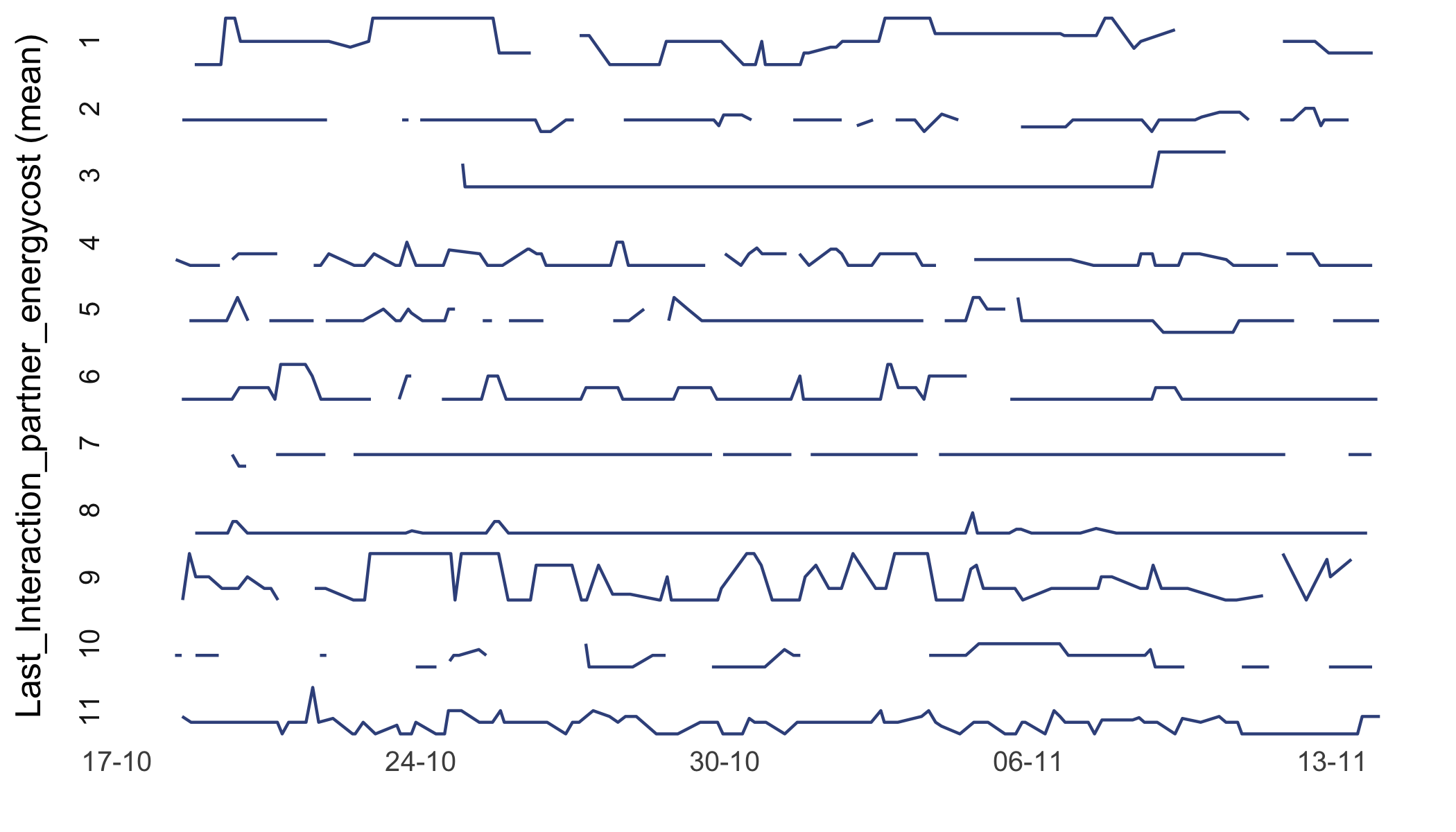


**Figure 45**

*Last Interaction, Interaction Partner* *Could Be Myself* *(Mean)*


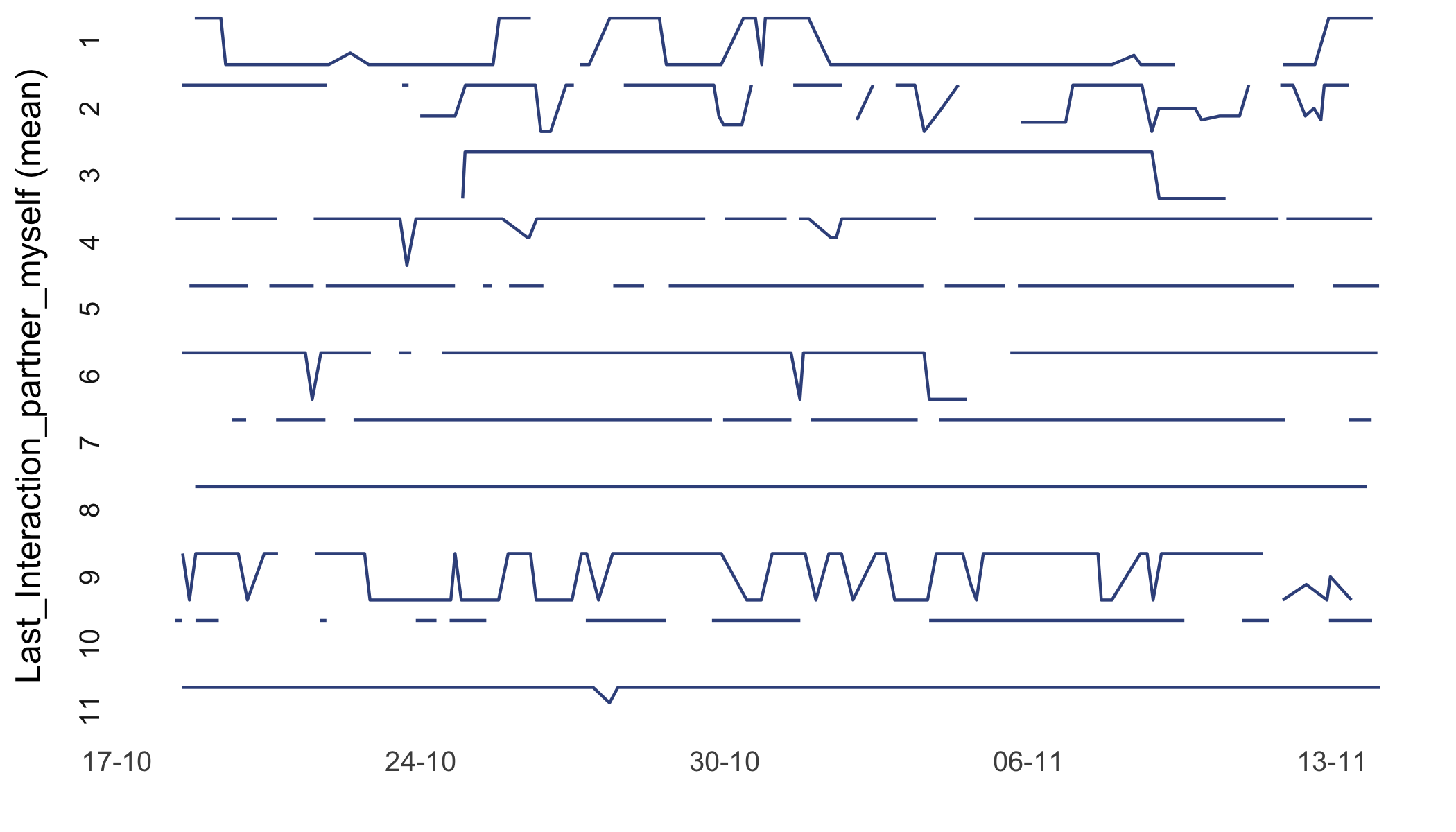


**Figure 46**

*Last Interaction, Interaction Partner* *Face-To-Face Contact Frequency* *(Mean)*


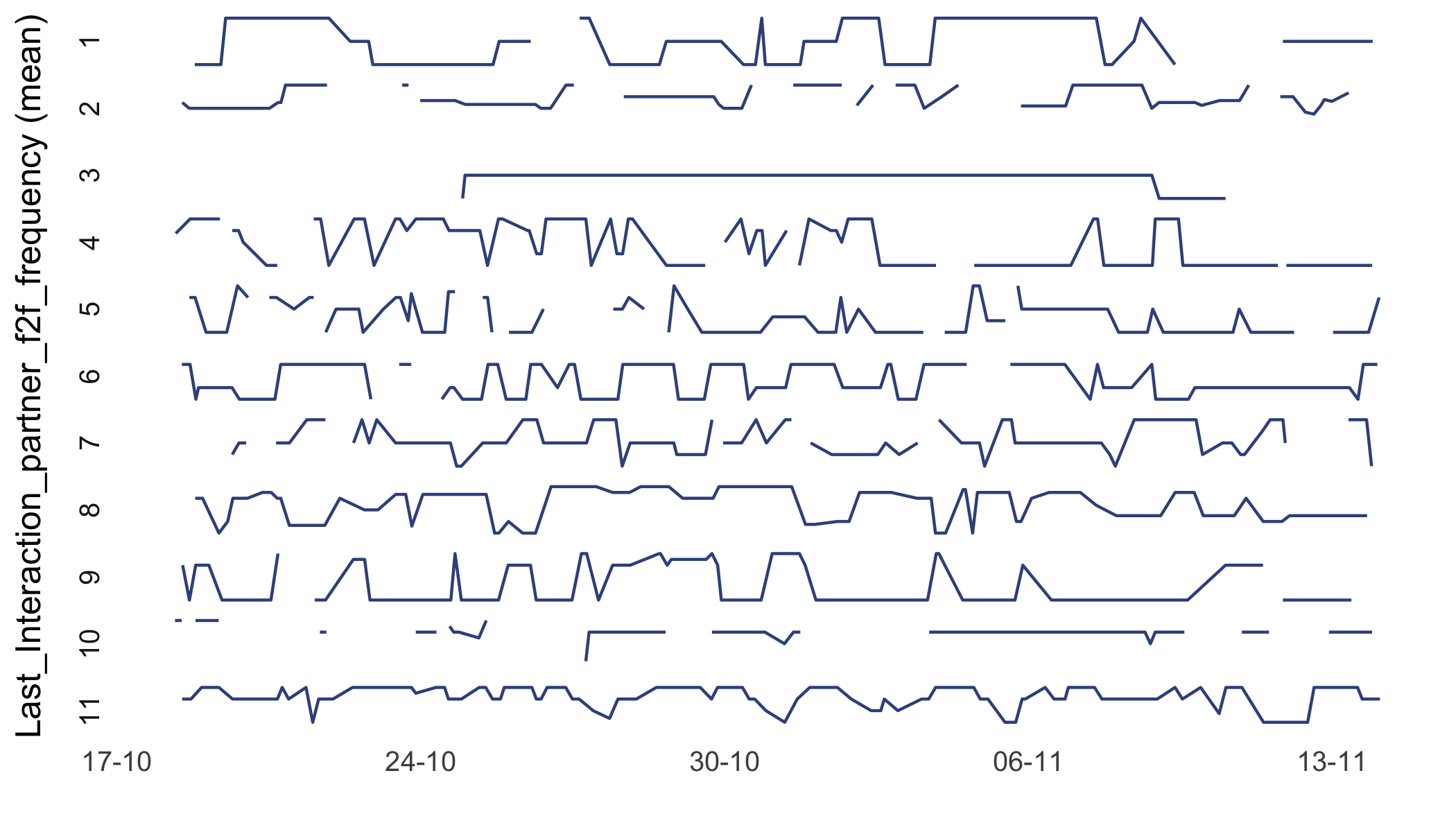


**Figure 47**

*Last Interaction, Interaction Partner* *Call Frequency* *(Mean)*


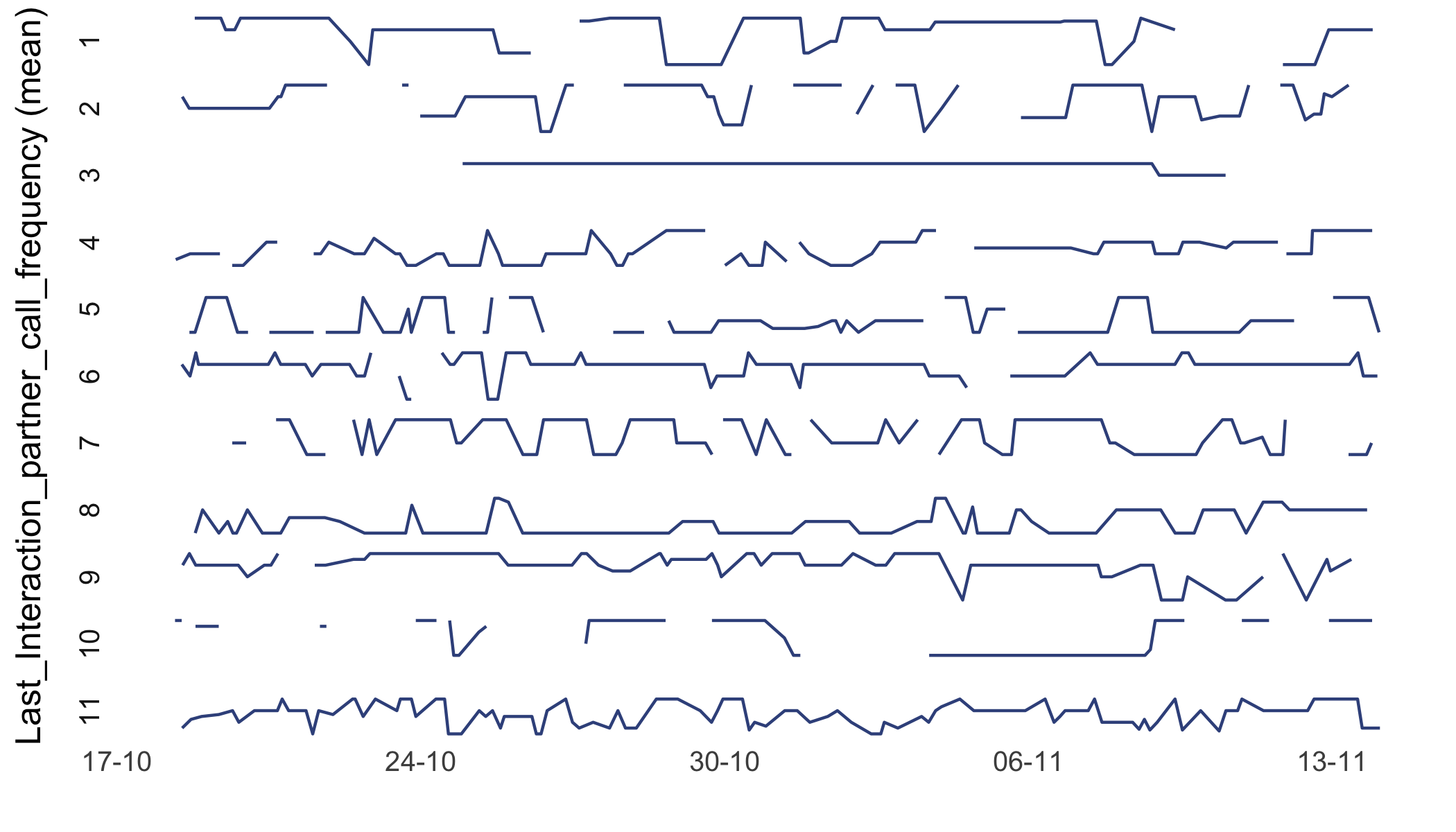


**Figure 48**

*Last Interaction, Interaction Partner* *Text Frequency* *(Mean)*
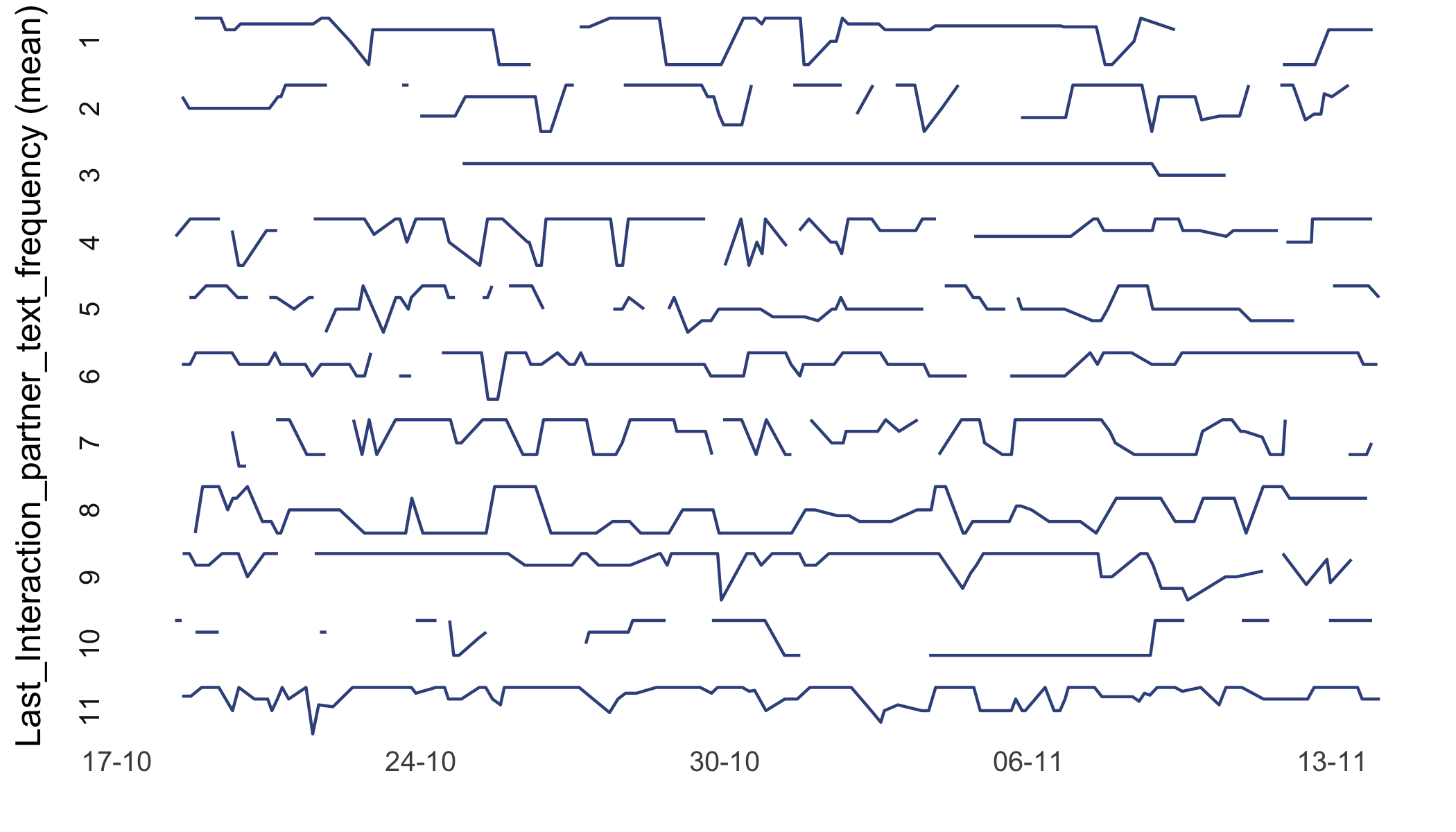


**Figure 49**

*Negative Affect* *(Mean)*


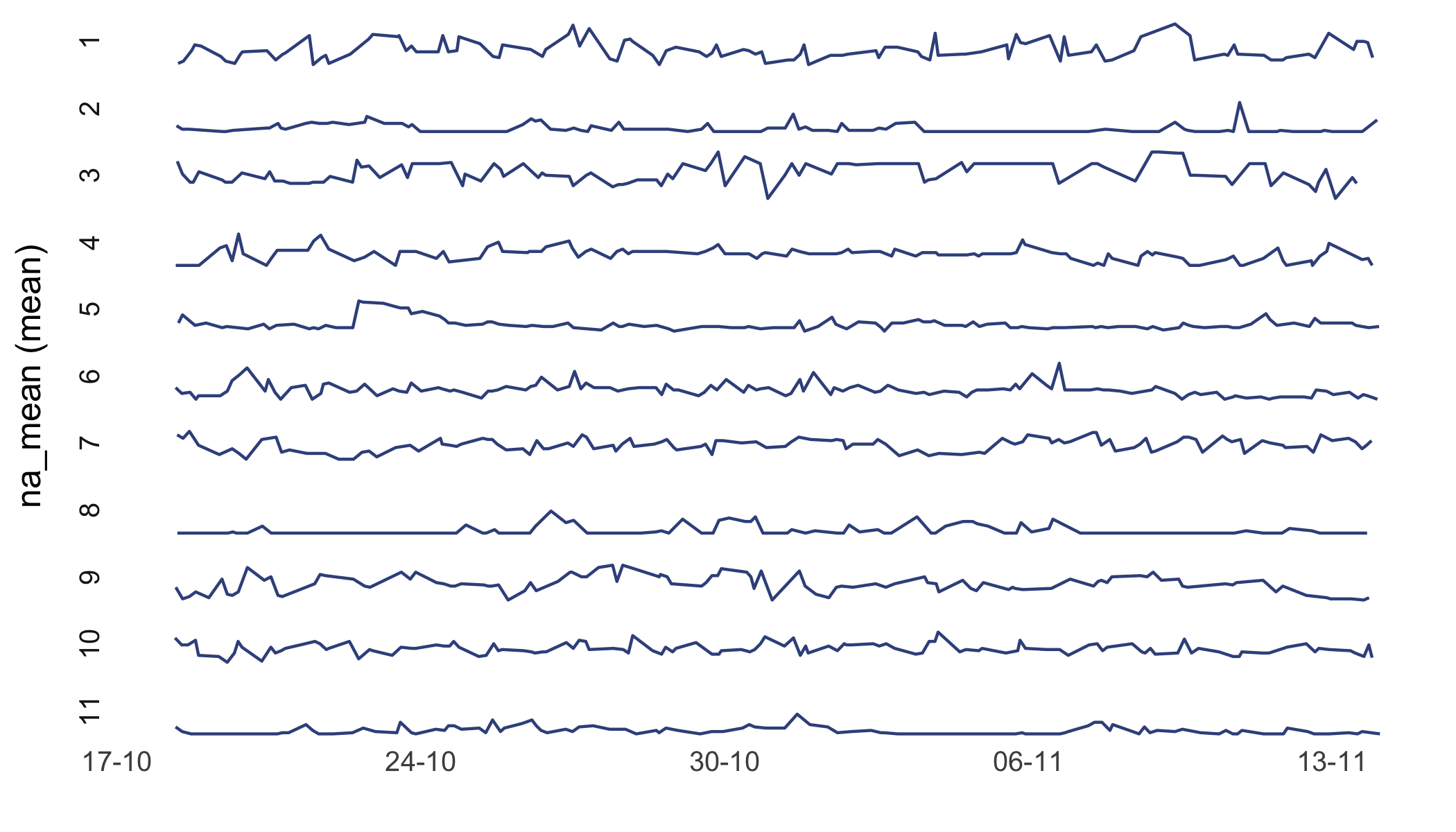


**Figure 50**

*Positive Affect* *(Mean)*


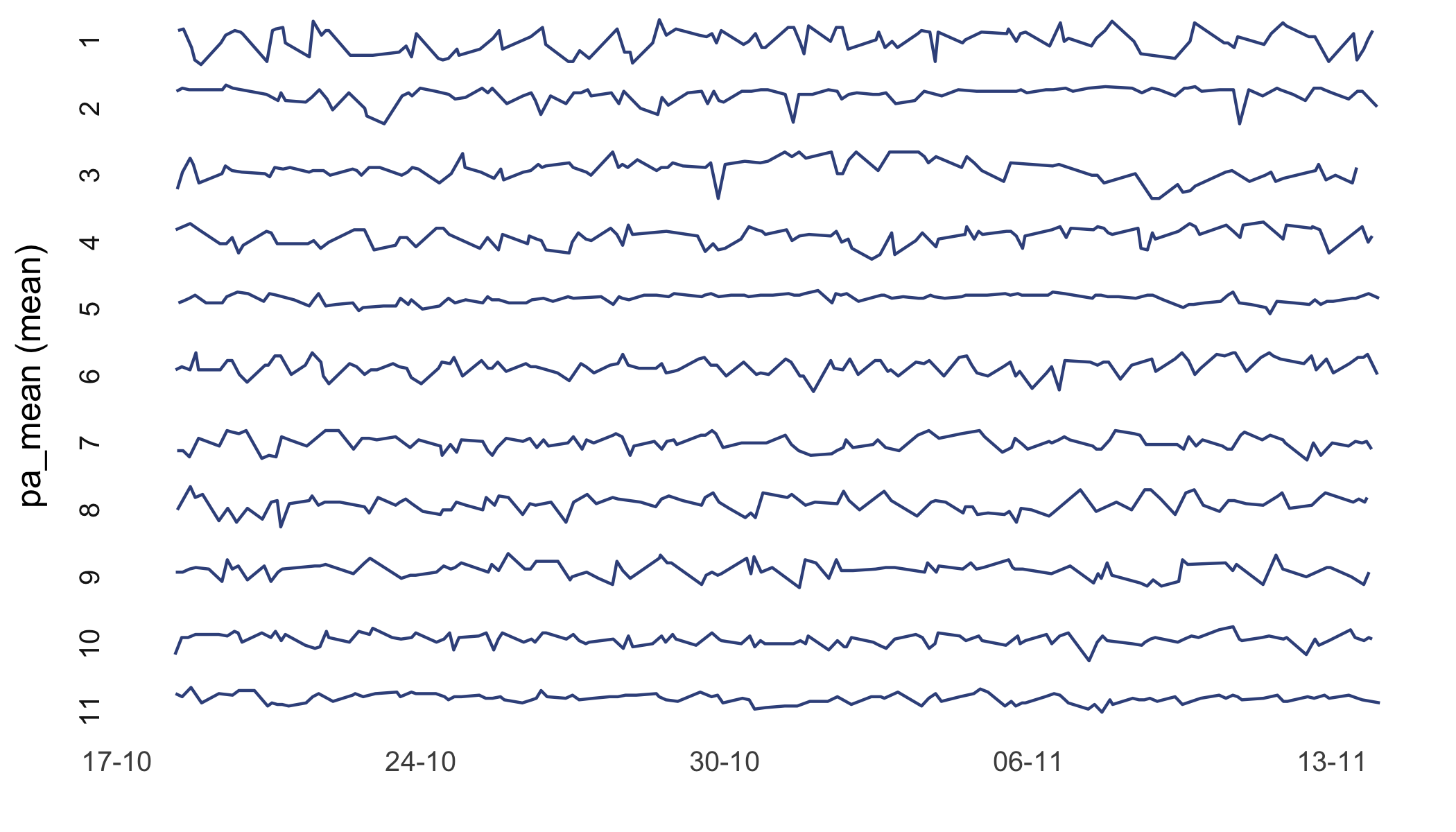


# Mean in the Training Set as Prediction

**Table 8**

*Results for Positive Affect When Using the Mean from the Test Set as Prediction, Including Standard Deviation and Minimum Value, Using Different Window Sizes and Levels of Aggregation*

|  | Predictors | Coefficient of Determination | Correlation | Mean Absolute Error | Mean Absolute Percentage Error | Window |
| --- | --- | --- | --- | --- | --- | --- |
| 1 | Training Set Mean | -0.03 (min = -0.06, SD = 0.01) | .14 (min = .08, SD = .03) | 2.03 (max = 2.07, SD = 0.06) | 50.87 (max = 51.26, SD = 2.73) | 20 |
| 2 | Training Set Mean | 0.03 (min = -0.03, SD = 0.04) | .24 (min = .16, SD = .04) | 1.19 (max = 1.25, SD = 0.07) | 17.91 (max = 20.51, SD = 2.05) | 30 |
| 3 | Training Set Mean | 0.04 (min = -0.09, SD = 0.07) | .32 (min = .04, SD = .15) | 1.56 (max = 1.8, SD = 0.12) | 44.57 (max = 54.67, SD = 5.24) | 15 |
| 4 | Training Set Mean | 0.01 (min = -0.04, SD = 0.03) | .2 (min = .16, SD = .02) | 1.49 (max = 1.49, SD = 0.01) | 25.22 (max = 25.62, SD = 0.29) | 30 |
| 5 | Training Set Mean | 0.17 (min = -0.11, SD = 0.15) | .46 (min = .19, SD = .14) | 0.67 (max = 0.71, SD = 0.02) | 8.9 (max = 9.03, SD = 0.11) | 15 |
| 6 | Training Set Mean | 0.02 (min = -0.06, SD = 0.04) | .2 (min = .09, SD = .05) | 1.35 (max = 1.41, SD = 0.03) | 19.72 (max = 21.02, SD = 0.67) | 20 |
| 7 | Training Set Mean | -0.08 (min = -0.18, SD = 0.05) | -.14 (min = -.31, SD = .08) | 1.18 (max = 1.24, SD = 0.03) | 22.69 (max = 23.11, SD = 0.32) | 20 |
| 8 | Training Set Mean | -0.09 (min = -0.15, SD = 0.03) | 0 (min = -.12, SD = .06) | 1.39 (max = 1.44, SD = 0.04) | 21.06 (max = 21.69, SD = 0.56) | 20 |
| 9 | Training Set Mean | -0.04 (min = -0.09, SD = 0.03) | -.11 (min = -.11, SD = .06) | 1.44 (max = 1.44, SD = 0.02) | 22.54 (max = 22.54, SD = 0.53) | 30 |
| 10 | Training Set Mean | 0.01 (min = -0.05, SD = 0.03) | .16 (min = .07, SD = .05) | 0.9 (max = 0.92, SD = 0.01) | 15.07 (max = 15.49, SD = 0.24) | 20 |
| 11 | Training Set Mean | -0.03 (min = -0.21, SD = 0.09) | .1 (min = -.03, SD = .07) | 0.72 (max = 0.82, SD = 0.05) | 9.02 (max = 9.95, SD = 0.46) | 30 |

**Table 9**

*Results for Negative Affect When Using the Mean from the Test Set as Prediction, Including Standard Deviation and Minimum Value, with Different Window Sizes and Levels of Aggregation*

|  | Predictors | Coefficient of Determination | Correlation | Mean Absolute Error | Mean Absolute Percentage Error | Window |
| --- | --- | --- | --- | --- | --- | --- |
| 1 | Training Set Mean | -0.09 (min = -0.12, SD = 0.01) | .04 (min = -.11, SD = .08) | 1.67 (max = 1.67, SD = 0) | 51.66 (max = 52.76, SD = 1.31) | 15 |
| 2 | Training Set Mean | -0.01 (min = -0.14, SD = 0.07) | .13 (min = 0, SD = .07) | 0.66 (max = 0.74, SD = 0.04) | 43.16 (max = 48.65, SD = 3.02) | 30 |
| 3 | Training Set Mean | -0.07 (min = -0.15, SD = 0.04) | .02 (min = -.03, SD = .06) | 1.89 (max = 1.98, SD = 0.06) | 38.26 (max = 43.89, SD = 3.92) | 30 |
| 4 | Training Set Mean | 0.14 (min = 0.07, SD = 0.04) | .39 (min = .33, SD = .03) | 0.81 (max = 0.85, SD = 0.02) | 37.89 (max = 42.82, SD = 2.48) | 20 |
| 5 | Training Set Mean | -0.13 (min = -1.05, SD = 0.52) | .2 (min = -.12, SD = .17) | 0.85 (max = 0.86, SD = 0.07) | 27.89 (max = 31.85, SD = 1.98) | 15 |
| 6 | Training Set Mean | 0.02 (min = -0.03, SD = 0.02) | .23 (min = .2, SD = .02) | 0.86 (max = 0.91, SD = 0.03) | 35.3 (max = 38.42, SD = 1.58) | 20 |
| 7 | Training Set Mean | -0.11 (min = -0.19, SD = 0.04) | -.01 (min = -.01, SD = .03) | 1.05 (max = 1.17, SD = 0.07) | 19.76 (max = 23.58, SD = 2.16) | 30 |
| 8 | Training Set Mean | 0 (min = -0.08, SD = 0.04) | .18 (min = .01, SD = .09) | 0.83 (max = 0.92, SD = 0.06) | 52.33 (max = 56.67, SD = 2.89) | 20 |
| 9 | Training Set Mean | -0.07 (min = -0.21, SD = 0.07) | .02 (min = 0, SD = .01) | 1.47 (max = 1.47, SD = 0.02) | 47.36 (max = 51.31, SD = 3.3) | 30 |
| 10 | Training Set Mean | 0 (min = -0.02, SD = 0.01) | .11 (min = .07, SD = .02) | 0.88 (max = 0.88, SD = 0.01) | 18.55 (max = 18.55, SD = 0.3) | 20 |
| 11 | Training Set Mean | -0.02 (min = -0.16, SD = 0.07) | .11 (min = 0, SD = .07) | 0.66 (max = 0.71, SD = 0.02) | 42.76 (max = 43.87, SD = 1.42) | 30 |

# Robustness Check Participant 5

**Figure 51**

*Robustness of* R^2^ *and Mean Average Percentage Error for Predicting Negative Affect*

*
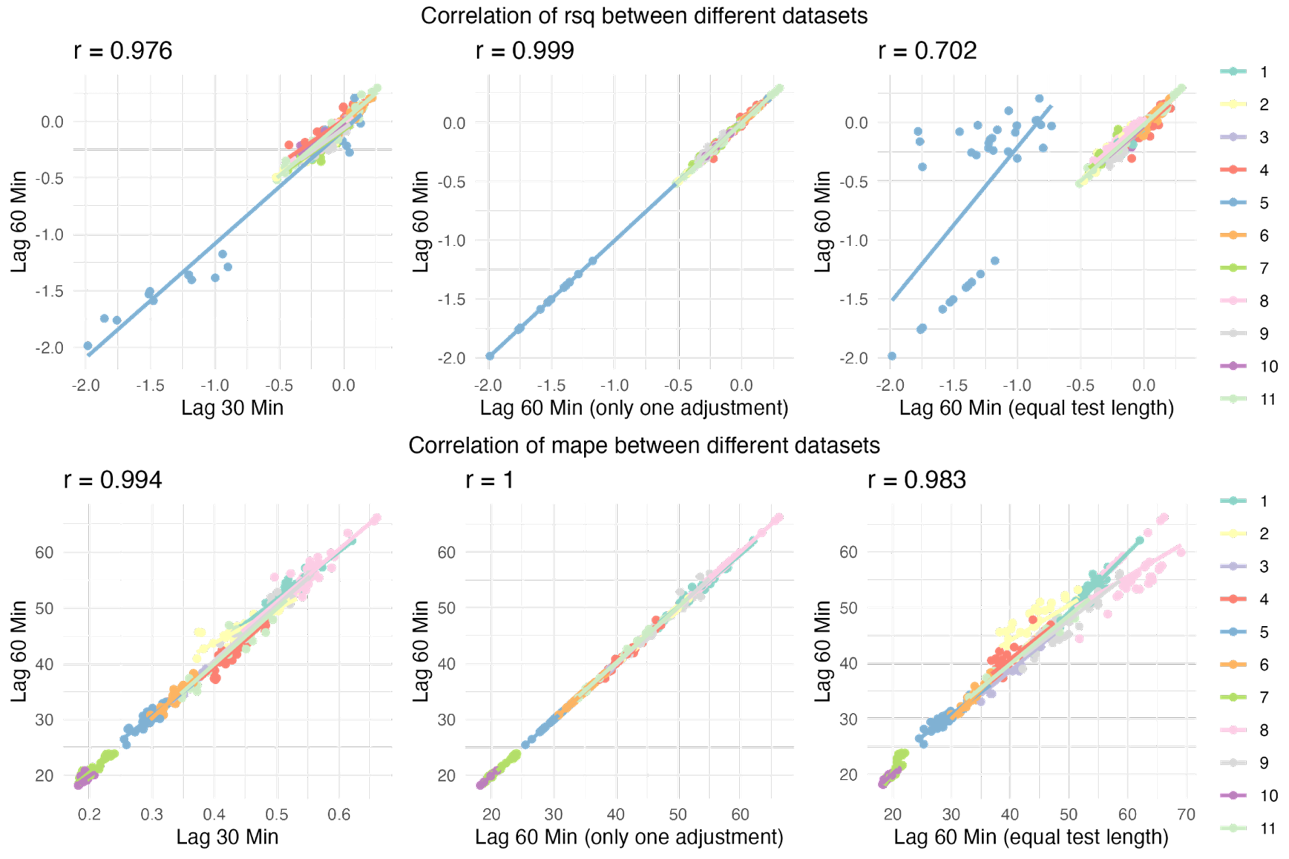
*

# Data Quality

This section provides a brief overview about the data quality. In the present study, the data from Behapp was not recorded properly for three out of fifteen participants, resulting in less than 75% of daily data being available for analysis. Consequently, these participants were excluded from our final analysis (as reported in the paper). For the remaining participants data coverage per hour was above 85% (see Table 10). We decided to mark app usage data, raw GPS data, Wi-Fi data and screen data as missing if no data was recorded from these sensors for 24 hours. In this study, this only resulted in missing data for WIFI (24 observations, < 0.01%).

**Table 10**

*Data Quality Metrics of Included Participants*

| Participant | Expected Hours of Data | Total Hours of Data | Coverage (Total Hours/Expected Hours) | % of days with location data | % of 10min-Gaps Location Data |
| --- | --- | --- | --- | --- | --- |
| 1 | 708 | 699 | 98% | 100% | 0.31% |
| 2 | 705 | 693 | 98% | 100% | 0.47% |
| 3 | 706 | 700 | 99% | 100% | 0.3% |
| 4 | 705 | 695 | 98% | 100% | 0.37% |
| 5 | 710 | 691 | 97% | 100% | 0.14% |
| 6 | 710 | 615 | 86% | 100% | 0.3% |
| 7 | 708 | 611 | 86% | 100% | 0.25% |
| 8 | 704 | 704 | 100% | 100% | 0.03% |
| 9 | 704 | 704 | 100% | 100% | 0.22% |
| 10 | 706 | 606 | 85% | 100% | 0.4% |
| 11 | 704 | 698 | 99% | 100% | 0.34% |
